# Limited clinical validity of univariate resting-state EEG markers for classifying seizure disorders

**DOI:** 10.1093/braincomms/fcad330

**Published:** 2023-11-30

**Authors:** Irene Faiman, Rachel Sparks, Joel S Winston, Franz Brunnhuber, Naima Ciulini, Allan H Young, Paul Shotbolt

**Affiliations:** Department of Psychological Medicine, King’s College London Institute of Psychiatry Psychology and Neuroscience, London SE5 8AB, UK; School of Biomedical Engineering & Imaging Sciences, King’s College London, London SE1 7EH, UK; Department of Basic and Clinical Neurosciences, Institute of Psychiatry, Psychology and Neuroscience, King’s College London, London SE5 8AB, UK; Department of Clinical Neurophysiology, King’s College Hospital NHS Foundation Trust, London SE5 9RS, UK; Department of Clinical Neurophysiology, King’s College Hospital NHS Foundation Trust, London SE5 9RS, UK; Department of Clinical Neurophysiology, King’s College Hospital NHS Foundation Trust, London SE5 9RS, UK; Department of Psychological Medicine, King’s College London Institute of Psychiatry Psychology and Neuroscience, London SE5 8AB, UK; South London and Maudsley NHS Foundation Trust, Bethlem Royal Hospital, Beckenham, Kent BR3 3BX, UK; Department of Psychological Medicine, King’s College London Institute of Psychiatry Psychology and Neuroscience, London SE5 8AB, UK

**Keywords:** epilepsy, psychogenic non-epileptic seizures, diagnostic accuracy, power, hctsa

## Abstract

Differentiating between epilepsy and psychogenic non-epileptic seizures presents a considerable challenge in clinical practice, resulting in frequent misdiagnosis, unnecessary treatment and long diagnostic delays. Quantitative markers extracted from resting-state EEG may reveal subtle neurophysiological differences that are diagnostically relevant. Two observational, retrospective diagnostic accuracy studies were performed to test the clinical validity of univariate resting-state EEG markers for the differential diagnosis of epilepsy and psychogenic non-epileptic seizures. Clinical EEG data were collected for 179 quasi-consecutive patients (age > 18) with a suspected diagnosis of epilepsy or psychogenic non-epileptic seizures who were medication-naïve at the time of EEG; 148 age- and gender-matched patients subsequently received a diagnosis from specialist clinicians and were included in the analyses. Study 1 is a hypothesis-driven study testing the ability of theta power and peak alpha frequency to classify people with epilepsy and people with psychogenic non-epileptic seizures, with an advanced machine learning pipeline. The next study (Study 2) is data-driven; a high number of quantitative EEG features are extracted and a similar machine learning approach as Study 1 assesses whether previously unexplored univariate EEG measures show promise as diagnostic markers. The results of Study 1 suggest that EEG markers that were previously identified as promising diagnostic indicators (i.e. theta power and peak alpha frequency) have limited clinical validity for the classification of epilepsy and psychogenic non-epileptic seizures (mean accuracy: 48%). The results of Study 2 indicate that identifying univariate markers that show good correlation with a categorical diagnostic label is challenging (mean accuracy: 45–60%). This is due to a considerable overlap in neurophysiological features between the diagnostic classes considered in this study, and to the presence of more dominant EEG dynamics such as alterations due to temporal proximity to epileptiform discharges. Markers that were identified in the context of previous epilepsy research using visually normal resting-state EEG were found to have limited clinical validity for the classification task of distinguishing between people with epilepsy and people with psychogenic non-epileptic seizures. A search for alternative diagnostic markers uncovered the challenges involved and generated recommendations for further research.

## Teaser

Faiman *et al*. explored whether resting-state EEG markers could differentiate between the diagnoses of epilepsy and psychogenic non-epileptic seizures. Two diagnostic accuracy studies (*n* = 148) tested several univariate markers using machine learning. Classification accuracy was low (45–60%), indicating challenges due to neurophysiological profiles overlap and stronger EEG dynamics in the data.

## Graphical Abstract

**Graphical Abstract:**
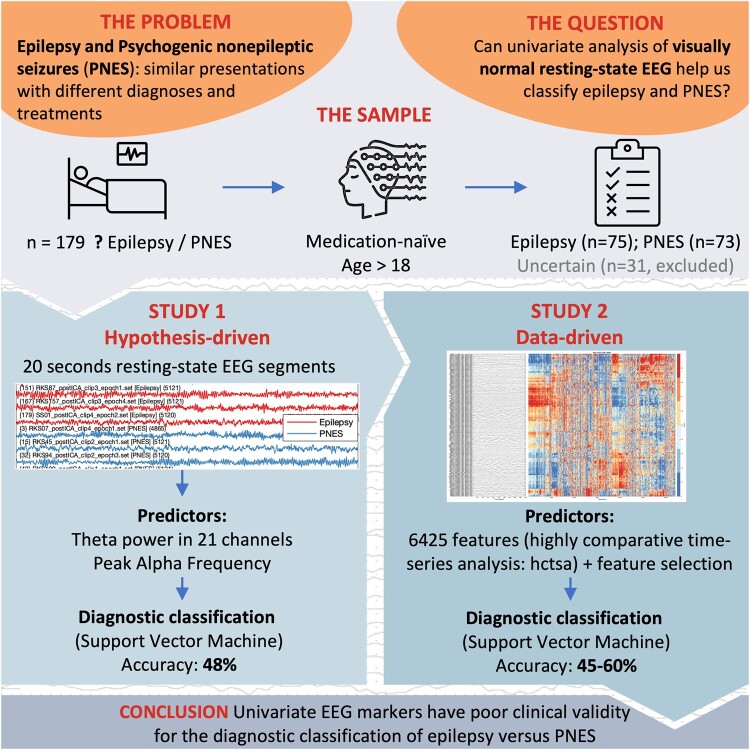

## Introduction

Considerable clinical challenges are involved in the diagnosis of seizure disorders; the most common problem involves differentiating epilepsy from epilepsy mimics such as psychogenic non-epileptic seizures (PNES) and syncope. Misdiagnosis rates for adults with epilepsy can be as high as 26% when the diagnosis is made by non-specialist medical professionals.^1^ Similarly, epilepsy mimics such as PNES are frequently misdiagnosed as epilepsy, and on average, people spend 7 years receiving inappropriate management and treatment before the diagnosis of PNES is reached.^2^

Considerable effort has been devoted to identifying measurable biological characteristics that could aid clinicians in the diagnosis of seizure presentations. Quantitative analysis of resting-state EEG signals has been implemented to understand whether epilepsy and PNES are associated with alterations in spontaneous electrophysiological activity. Our recent systematic review summarized 26 studies exploring group differences or diagnostic accuracy of markers extracted from interictal, visually normal EEG segments of adults with idiopathic epilepsy (i.e. non-lesional epilepsies of unknown or presumed genetic aetiology) or PNES.^3^ Results suggested that the resting-state EEG in idiopathic epilepsy is characterized by increased theta power as compared to controls, and has a pattern of EEG slowing as indicated by a shift of power and power peak towards lower frequencies. Conversely, no clear pattern could be identified from the few studies comparing PNES cohorts to controls. The latest evidence continues to support these findings.^4,5^ This encourages an exploration of these markers as potential discriminators between a diagnosis of epilepsy and one of PNES.

Notably, only two studies so far have directly compared cohorts with idiopathic epilepsy and PNES on resting-state EEG measures. Bernasconi *et al.*^6^ investigated delta (1–4 Hz) amplitude, reporting no group differences. Cao *et al.*^7^ explored group differences and diagnostic accuracy of correlation and coherence measures, reporting 55–70% classification accuracy for a set of selected markers. However, methodological considerations (e.g. lack of control for antiseizure medication effects and circadian variations) and analytical considerations (e.g. use of a case–control design, circular analysis and data leakage between training and test sets) limit the interpretability and generalizability of these findings. The question of whether people with epilepsy and PNES can be differentiated on resting-state EEG characteristics remains underexplored.

We conducted two sequential studies. In a first, theory-driven diagnostic accuracy study, we explored whether resting-state EEG markers identified through our systematic review (theta power and peak alpha frequency)^3^ have the potential to aid in the classification problem of distinguishing epilepsy and PNES. In a second study, a data-driven exploratory approach was implemented; a large number of features were computed using a recently developed feature extraction tool.^8^ No a priori hypotheses were made as to individual features’ relevance to the diagnostic classification. By working with a large pool of measures based on different theoretical frameworks, we aimed to identify markers that had good correlation with diagnostic labels in our cohort and might therefore be clinically meaningful.

Faiman *et al.*^3^ highlighted the need to control for sources of bias and address shortcomings in study designs to improve the analytical and clinical validity of diagnostic markers. Towards this end, this study implemented validated automated pipelines to maximize the generalizability and reproducibility of EEG analyses. Reporting complies with reproducibility, diagnostic prediction models and STROBE guidelines.^9,10^ Efforts were made to control for common sources of bias such as EEG artefacts, differences in demographic characteristics, medication effects, alertness, circadian variation and incorporation bias. The study design was optimized for assessing the clinical validity of diagnostic accuracy indices; all patients suspected of having epilepsy or PNES and meeting inclusion criteria were quasi-consecutively selected. EEG data extracted for analysis were performed from unmedicated patients prior to diagnosis. A diagnosis was subsequently reached by specialist clinicians for most patients. Unlike case–control designs, the diagnostic accuracy design implemented mimics the population in which EEG markers would be used to inform the diagnostic decision.^11^

## Materials and methods

### Participants

We retrospectively identified a quasi-consecutive cross-sectional sample of people presenting to King’s College Hospital specialist clinics with a suspected seizure disorder (see Supplementary Material Section 1 for patient identification strategy). Inclusion criteria were age over 18 and not taking any CNS agents at the time of EEG [including antiseizure medications (ASMs), antidepressants, anxiolytics, opioids, etc.]. Exclusion criteria were acute symptomatic seizures, abnormal CT/MRI, eventual diagnosis of concurrent epilepsy and PNES, relevant history of other neurological, neurodevelopmental or severe mental health disorders (details in Supplementary Material Section 1). Age and gender matching for people eventually diagnosed with epilepsy or PNES was achieved as described in Supplementary Material Section 1. An established diagnosis of epilepsy was a clinical diagnosis in accordance with operational clinical definitions.^12^ An established diagnosis of PNES was considered either a video-EEG supported diagnosis in accordance with ILAE definitions,^13^ or in the absence of video-EEG confirmation, a strong clinical indication of PNES following review by both a specialist neurologist and neuropsychiatrist. A minimum sample size of 130 participants was defined a priori.^14^ The study was approved by the London Queen Square Research Ethics Committee (REC: 20/LO/0784, IRAS ID: 265164, 28/09/2021). Patient consent for analysis of anonymized data was not required.

### EEG data acquisition

Clinical EEG data were acquired at King’s College Hospital Department of Neurophysiology in unshielded and artificially lit rooms. Twenty-one scalp electrodes (Hurev, silver disk, 1.5Ø DIN connector) were placed according to the Modified Maudsley Configuration.^15,16^ This is similar to the 10–20 system, with slightly different positioning (∼20 mm lower) of the temporal electrodes chain for optimal frontal and temporal coverage.^17^ Impedances were kept below 5 kΩ (or 10 kΩ under particular circumstances), checked at electrode placement and throughout the appointment. The reference channel was typically placed on the midline anterior to Pz. Single-channel ECG was simultaneously recorded. EEG data were acquired using either the NicoletOne EEG System v44 (Viasys Healthcare, with Viasys Healthcare NicoletOne M40 Amplifier, 40-channel, sampling rate 256 Hz and analogue filter bandwidth 0.053–500 Hz, with additional acquisition filters set at 0.5–70 Hz) or the Xltek EEG32U system (Natus, with Nicolet v32 Amplifier, 32-channel, sampling rate 500 Hz and analogue filter bandwidth 0.053–500 Hz).

### EEG data selection

The first available EEG recording during which the patient was aged over 18 and was not taking any CNS agents was reviewed for inclusion. Resting-state EEG data to be used for the analyses were inspected (consistently using common average and then longitudinal bipolar montage) and selected by a trained band 6 EEG technician at King’s College Hospital (N.C.) with 2.5 years of clinical experience who was blinded to diagnosis, referral question, and any patient details. Data segments of minimum 20 s were selected from the controlled baseline period (awake, eyes-closed, relaxed recordings in lying position) that were normal on visual inspection, i.e. did not include any slowing, interictal epileptiform discharges (IEDs), non-specific or dubious abnormalities, periods of drowsiness or sleep, or major artefacts.

### EEG data preprocessing

Data acquired with the Xltek EEG32U system were downsampled to 256 Hz, high-pass filtered at 0.5 Hz and down-pass filtered at 70 Hz (Hamming windowed sinc FIR filter) to match the sampling rate and acquisition filters of the NicoletOne EEG system. The PREP pipeline was implemented to remove externally generated experimental artefacts (line noise and bad channels), to interpolate bad channels (spherical interpolation: EEGLAB ‘eeg_interp()’ function; mean 1.9 channels (SD 1.5) interpolated per segment)^18^ and to calculate a reliable and robust average reference.^19^ Independent Component Analysis (ICA) was run using the extended Infomax algorithm.^20^ The number of ICs generated was rank-adjusted (18 ICs generated on average)^21^ and IClabel^22^ was then implemented to remove ICs that had ≥80% probability of being generated by muscles, eyes, heart, line noise or channel noise (1.9 ICs removed on average).^23,24^ All data were visually inspected and then segmented into 20 s non-overlapping segments to maintain consistency with previous studies.^3^ One 20 s segment per participant was selected at random for analyses.

### EEG features extraction

#### Study 1

The 20 s segments were segmented in 2 s epochs. Spectral information was obtained for each of the 21 EEG channels by means of Fast Fourier Transformation using the FieldTrip toolbox for EEG analysis (http://fieldtriptoolbox.org)^25^ in MATLAB (R2021a, Natick, MA: The MathWorks Inc.), implementing a multi-taper method with a single (Hanning) taper window to reduce spectral leakage.^26^ A sliding window was set to 50 ms. The frequency resolution was 0.5 Hz. Power values were averaged over epochs to obtain a single power density spectrum (μV^2^) for each 20 s segment.^27^ Each value was converted from μV^2^ to decibels (dB): y dB = 10 ∗ log10(y). The logarithmic transformation creates a close-to-normal distribution that is otherwise positively skewed due to the 1/f scaling of the power spectrum.^28^

Mean power was computed for each channel for each of the following frequency bands: delta (1–3.5 Hz), theta (4–7.5 Hz), alpha (8–12.5 Hz) and beta (13–30 Hz).^9^

The peak alpha frequency (PAF) is the frequency of the highest point in the spectral peak occurring within the alpha bandwidth.^29^ A global PAF index was computed for each 20 s segment by means of the MATLAB (R2021a) restingIAF open-source package v1.0.3 (https://github.com/corcorana/restingIAF).^30^ To derive the power spectrum (1–45 Hz), this implements pwelch, normalization and Savitzky–Golay filter smoothing (11 bins, polynomial degree of 5). PAF was identified using 21 EEG channels, with a minimum of three valid channels (i.e. with reliable peak, independently of spatial location) needed to support the global PAF estimation, and an alpha peak search window between 7 and 13 Hz, as per guidelines.^30^

To ensure that the EEG signal used for statistical analyses contained oscillatory activity over and above the aperiodic 1/f activity, EEG segments without a detectable alpha peak on a minimum of three channels (as detected by the restingIAF function) were excluded from further analyses.^31^ All patients had at least one usable 20 s segment.

#### Study 2

**Figure 1:**
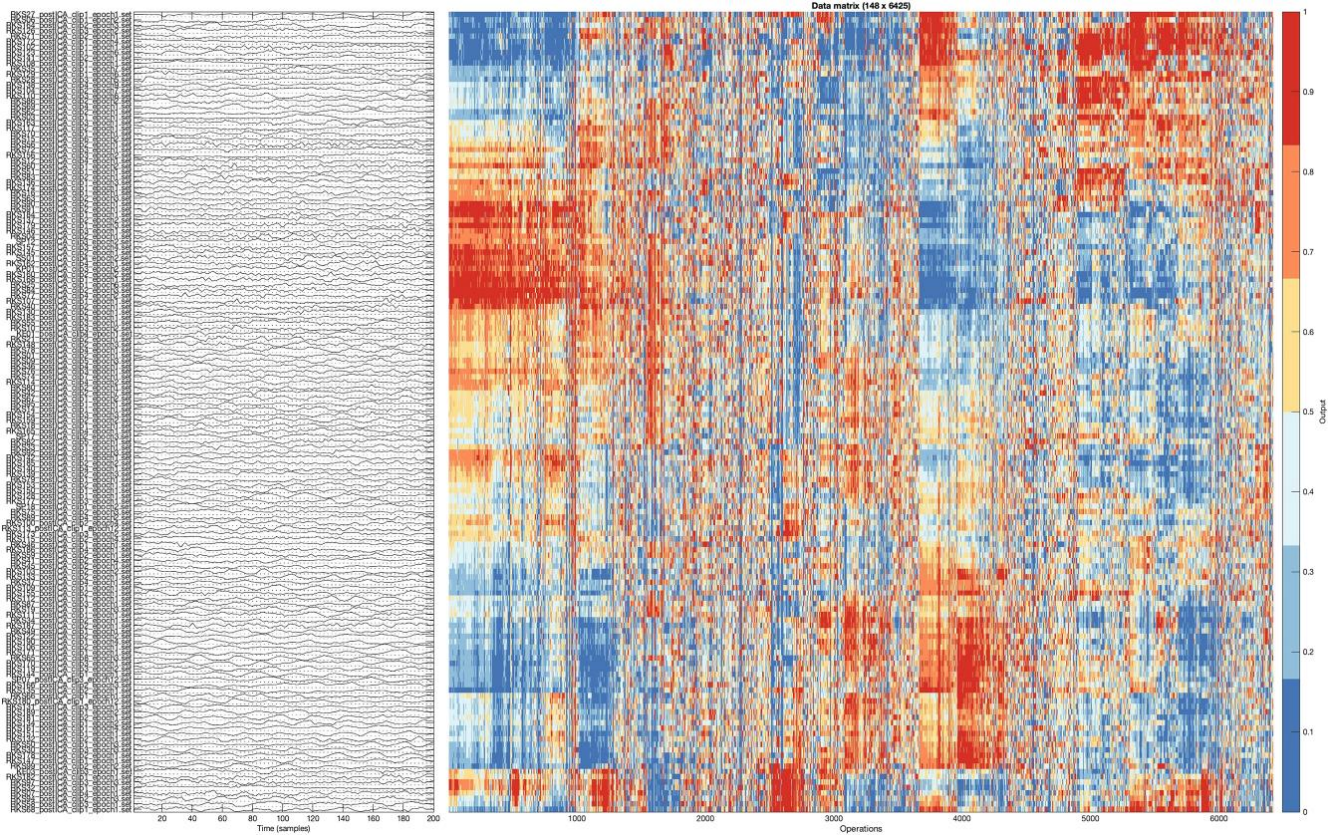
**Visual representation of hctsa feature extraction from the EEG signal (Study 2) for a sample channel (FP1).** For each patient (*y*-axis), the left subplot displays the first second of the 20-s EEG data segment analysed. The right subplot displays the normalized values for each of 6425 features (operations) computed from each segment, on a colour scale ranging between zero (blue) and one (red). Rows and columns are ordered by feature clusters for visualization purposes.

The highly comparative time series analysis (hctsa) software tool^8,32^ was implemented in MATLAB R2021a and used to compute a high number of features (i.e. measures to be used as predictor variables, *n* = 7729) from time-series (EEG) data. Hctsa is a univariate method, so the full set of 7729 features was extracted for each patient, and separately for each of the 21 EEG channels. Any features that had <100% valid values were removed, resulting in a total of 6425 valid features (Fig. 1). Further details on hctsa and the retained features are in Supplementary Material Section 3.

### Model fitting

The objective of model fitting was to determine whether the features extracted could discriminate between the diagnostic classes (epilepsy/PNES). The key steps of the model fitting process are outlined below; in-depth technical details are in Supplementary Material Sections 2 and 3.

#### Study 1

**Figure 2:**
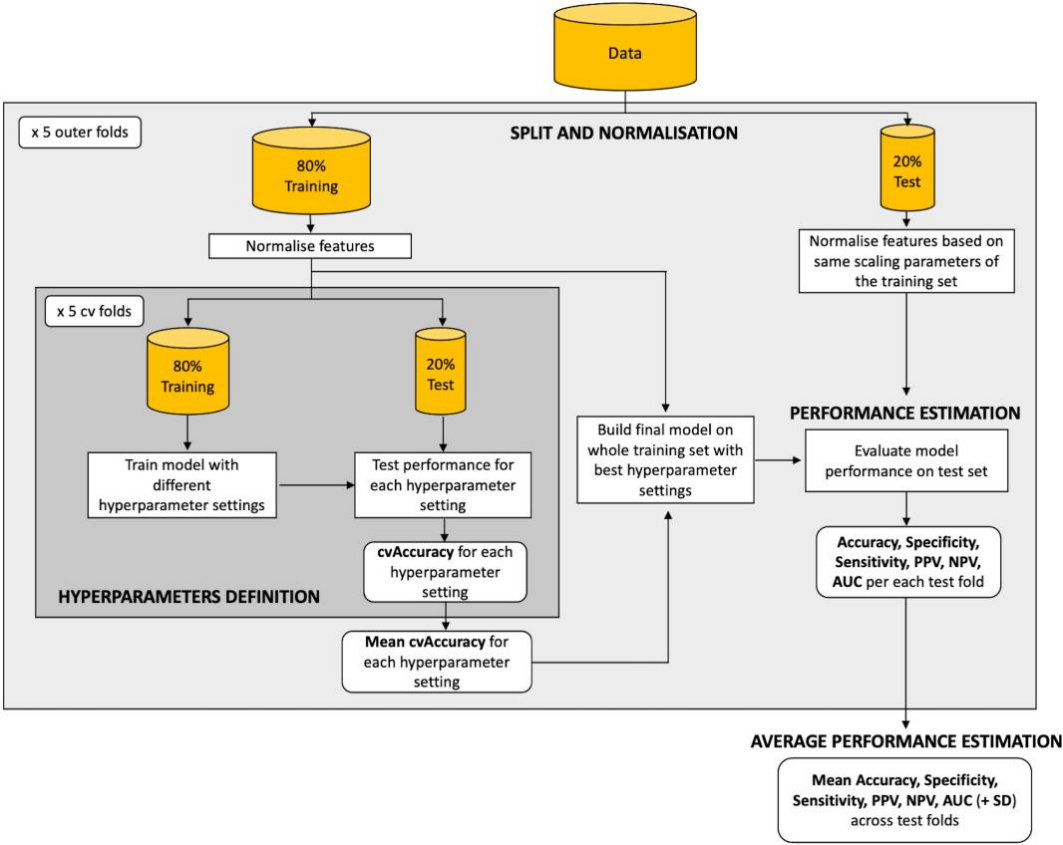
**Support vector machine pipeline for Study 1.** cv, cross-validation; PPV, positive predictive value; NPV, negative predictive value; AUC, area under the curve; SD, standard deviation.

To test whether theta power (in 21 channels) and global PAF could predict the binary diagnostic class, a Support Vector Machine (SVM) model was fit to the data^33^ using the scikit-learn software in Python v3.10.4.^34^ The whole dataset was divided at the patient level into a training set (80%) and a test set (20%), stratifying by diagnosis (Fig. 2). Features were normalized, and a SVM model with radial basis function^35^ was implemented on the training set. We optimized hyperparameters via grid search, followed by a 5-fold cross-validation to identify the optimal kernel parameter combination. The final model was built and assessed on the test set. This process was iterated five times on different training and test sets. Average model performance is reported.

#### Study 2

We evaluated whether the 6425 features extracted could predict diagnostic class y. The same analysis pipeline as the one described for Study 1 was followed, with the addition of a feature selection step on the training set (Supplementary Fig. 11). This step involved using a filter-based feature selection method, the minimum Redundancy—Maximum Relevance algorithm (mRMR),^36^ to identify informative features. These were normalized and retained for evaluation on the test set. The rest of the analysis pipeline is identical to Study 1. For Study 2, the whole procedure was repeated independently for each of the 21 EEG channels. Features that were repeatedly selected as informative are reported (Supplementary Fig. 11).

### Subgroup analyses

To explore whether the model achieved a higher accuracy for certain patient subgroups, a series of analyses were run for both studies measuring performance for patients grouped by:

1) different method of confirmation of diagnosis (video-EEG confirmation / clinical diagnosis only);
2) different epilepsy types (focal / generalized / unclassified); and
3) different overall outcome for EEG examination studied (normal / abnormal with non-specific findings / abnormal with epileptiform features).

Accuracy indices for each subgroup were calculated based on the observed and predicted diagnosis for each patient.

### Control and *post hoc* analyses

For both studies, control analyses were performed on different split ratios (70:30 and 90:10) as training set size can influence machine learning algorithm accuracy.^37^ To ensure results are not dependent on EEG segment choice, analyses were repeated for two different randomly selected 20 s segments per participant (hereafter named random segments 2 and 3). For the minority of participants with fewer than three 20 s segments (*n* = 28 with two; *n* = 10 with one), random segments 1 or 2 were reused as control segments (Supplementary Material Section 4).

#### Study 1

In a further control analysis, the performance of a model built solely on theta power measures, and one built solely on PAF was reported. Multiple independent samples t-tests were performed *post hoc* to report on group differences for each predictor. As there have been variable reports of EEG power being higher in people with epilepsy for other frequency bands (delta, alpha, beta),^3^ we also reported prediction accuracy of power measures for these frequency ranges.

#### Study 2

A series of *post hoc* analyses were conducted to aid interpretation of results, including synthetic dataset code validation, testing alternative feature selection methods, selecting different number of features, assessing intra-patient feature stability, running control analyses to rule out overfitting and testing the appropriateness of the classifier to represent the feature space and low-dimensional visualizations. Detailed methodology can be found in Supplementary Material Section 3.

### Population descriptive statistics

To estimate the significance of group differences for descriptive categorical variables, a χ^2^ test^38^ was used, or Fisher’s exact test^39^ in the presence of low cell count (*n* < 5).^40^ The Kolmogorov–Smirnov test was implemented to assess the normalcy of distributions.^41^ To estimate the significance of group differences for continuous variables, independent samples *t*-test were used in the presence of normal distributions, and a Mann–Whitney U test was used for other distributions.^42^

To control for circadian effects, the time of the day at which the EEG recording was taken was converted to radians (as per e.g. Abela *et al.*^43^) using the hms2rad function in the R package ‘astroFns’. The Watson-Williams test was used to compare group means of circular data (radians).^44^

Due to the retrospective nature of the study, some descriptive variables had missing data; these were handled by listwise case exclusion in descriptive statistics; the resulting sample size is reported.

### Data availability

Anonymized data are available upon request for collaborative purposes. The code used for the main analyses can be found in Supplementary Material Sections 4 and 5.

## Results

### Population characteristics

A total of 148 people (epilepsy *n* = 75; PNES *n* = 73) received a diagnosis by the time of data analysis and were included in the study; people with uncertain diagnosis (*n* = 29) were excluded (Supplementary Figs 1 and 2). A significantly higher number of people with PNES were left-handed or ambidextrous (Table 1). All patients were diagnosed by a consultant neurologist specialized in epilepsy and/or by a consultant neuropsychiatrist specialized in PNES. The diagnosis was supported by video-EEG in 80% of people with PNES (typical event captured whilst recording normal EEG) and in 57% of people with epilepsy (epilepsy-specific abnormalities captured) (Table 1).

**Table 1:** Demographic, clinical and EEG characteristics of the included population, with statistical group comparison

	Epilepsy (*n* = 75)	PNES (*n* = 73)	Statistic	*P*-value
Demographics				
Female gender, *n* (%)	47 (63)	47 (64)	χ^2^(1148) = 0.002	0.96
Age at time of EEG (years), mean (SD)	31.9 (12.4) Range 18–63	31.4 (11.3) Range 18–61	*t*(146) = 0.27	0.79
Right handedness, *n* (%)	71 (94.6) *n* = 75	54 (73.9) *n* = 70	Fisher’s exact	0.003
Substance use habits				
Smoking (nr cigarettes), median (IQR)	0 (2.0) *n* = 69	0 (2.25) *n* = 62	U = 2134.0	0.98
Alcohol (units/week), median (IQR)	1 (3.12) *n* = 70	0 (1.00) *n* = 64	U = 2698.0	0.02
Occasional recreational drugs use, *n* (%)	7 (9.3)	3 (4.1)	Fisher’s exact	0.32
Diagnosis				
vEEG confirmation, *n* (%)	43 (57.3)	58 (79.4)	χ^2^(1148) = 7.36	0.006
Years from first seizure to diagnosis, median (IQR)	2 (3.0)	3 (6.0)	U = 2376.5	0.16
Disorder characteristics				
Age at first seizure, mean (SD)	28.2 (13.3)	26.1 (10.4)	*t*(146) = 1.09	0.27
Disorder duration at EEG (years), median (IQR)	1 (3.0)	3 (5.0)	U = 2266.5	0.07
Monthly seizure frequency in 6 months prior to EEG, median (IQR)	0.33 (0.6) *n* = 72	4.25 (19.0) *n* = 72	U = 909.0	<0.001
Family history of epilepsy, *n* (%)	20 (27) *n* = 74	21 (29) *n* = 71	χ^2^(1145) = 0.02	0.87
Characteristics of the EEG recordings used for analyses				
Time recorded, am (versus pm), *n* (%)	33 (44.0)	40 (54.8)	χ^2^(1148) = 1.32	0.25
Time recorded (radians), mean (SD)	3.28 (0.7)	3.38 (0.6)	*F*(1148) = 1.73	0.19
Location of recordings				
Routine, *n* (%)	42 (56.0)	31 (42.5)	χ^2^(1, 148) = 2.19	0.13
Sleep, *n* (%)	31 (41.3)	17 (23.3)	χ^2^(1, 148) = 4.70	0.03
Activation, *n* (%)	1 (1.3)	22 (30.1)	Fisher’s exact	<0.001
HVT baseline, *n* (%)	1 (1.3)	3 (4.1)	Fisher’s exact	0.36
Acquisition System				
Xltek (versus Nicolet), *n* (%)	37 (49.3)	31 (42.5)	χ^2^(1, 148) = 0.45	0.50
EEG investigation overall outcome				
Normal, *n* (%)	22 (29.3)	57 (78.1)	χ^2^(1, 148) = 33.39	<0.001
Abnormal non-specific^a^, *n* (%)	25 (33.3)	14 (23.3)	χ^2^(1, 148) = 3.12	0.08
Slowing, *n* (%)	22 (29.3)	8 (10.9)	χ^2^(1, 148) = 6.63	0.01
Other, *n* (%)	26 (34.6)	11 (15.1)	χ^2^(1, 148) = 6.57	0.01
Abnormal epilepsy-specific^b^, *n* (%)	28 (37.3)	2 (2.7)	Fisher’s exact	<0.001
Seizures recorded^c^, *n* (%)	3 (4.0)	30 (41.1)	Fisher’s exact	<0.001

^a^Non-specific transient abnormalities refer to slowing or other non-specific transients (sharpened waves).

^b^Epilepsy-specific transient abnormalities refer to spikes, sharp waves, generalized spike and wave discharges, generalized polyspike-waves, generalized 3–4 Hz spikes.

^c^Seizures refer to epileptic seizures for the epilepsy group and non-epileptic seizures for the PNES group.

Relevant disorder characteristics were similar between groups, except for a higher monthly seizure frequency in PNES (Table 1). The epilepsy aetiology was unknown for the whole epilepsy sample. Epilepsy type was recorded as focal in 44% (*n* = 33), generalized in 27% (*n* = 20) and unknown/undefined in 29% (*n* = 22) of the sample.

The EEG recordings used were performed between April 2007 and August 2021 in different clinical EEG settings; the acquisition systems used were balanced across groups (Table 1). Groups did not differ in the time of the day during which recordings were taken, making confounding effects due to circadian rhythms unlikely (Table 1). Patients did not report any epileptic seizures in the 24 hours prior to EEG recordings (excluding PNES *n* = 32 and epilepsy *n* = 9 with unspecified latency from last seizure). At the time of EEG, the median days since last event were 60 (IQR: 75.25) for people with epilepsy and 7 (IQR: 20.0) for people with PNES (excluding missing). For 78% of people with PNES and 29% of people with epilepsy, the whole EEG examination was overall normal; for the remaining patients, some EEG abnormalities were recorded elsewhere in the same recording session (Table 1); EEG segments analysed contained no abnormalities.

### SVM results

#### Study 1

The SVM classifier was built to optimize accuracy for the two groups based on theta power in 21 channels and a global PAF index from one randomly selected EEG segment per patient. This resulted in a sensitivity of 57%, a specificity of 38% and an accuracy of 48%, indicating that the model predicts patients’ diagnosis no better than chance (Table 2).

**Table 2:** Classification performance for theta power in 21 channels and peak alpha frequency (PAF) for one randomly sampled 20 s EEG segment

Predictors	Accuracy	Sensitivity	Specificity	PPV	NPV	AUC
Theta (21 channels) and PAF	0.48 (0.05)	0.57 (0.22)	0.38 (0.21)	0.49 (0.05)	0.37 (0.19)	0.48 (0.05)

Reported are mean (SD) across 5 cross-validation folds (80:20 proportion for training and test sets).

PPV, positive predictive value; NPV, negative predictive value; AUC, area under the curve.

#### Study 2

For each channel, a SVM classifier was built to test the ability of selected feature sets to predict binary diagnostic class. Results indicated that the models had a poor prediction ability in all channels (Table 3). A number of features tended to be repeatedly selected across different split folds and across different channels; the most consistently selected was the *P*-value of the *z*-test applied to the time series (Supplementary Table 12).

**Table 3:** Classification performance for selected features in 21 channels for one randomly sampled EEG segment

Channel	Accuracy	Sensitivity	Specificity	PPV	NPV	AUC
FP1	0.52 (0.05)	0.52 (0.11)	0.52 (0.16)	0.53 (0.04)	0.50 (0.07)	0.52 (0.05)
FP2	0.55 (0.1)	0.55 (0.16)	0.55 (0.09)	0.55 (0.08)	0.55 (0.11)	0.55 (0.09)
F3	0.59 (0.06)	0.61 (0.14)	0.58 (0.07)	0.59 (0.05)	0.60 (0.08)	0.59 (0.06)
F4	0.55 (0.11)	0.65 (0.19)	0.44 (0.11)	0.54 (0.09)	0.58 (0.18)	0.55 (0.11)
C3	0.60 (0.08)	0.65 (0.1)	0.55 (0.2)	0.62 (0.11)	0.60 (0.06)	0.60 (0.08)
C4	0.54 (0.1)	0.56 (0.14)	0.52 (0.12)	0.54 (0.1)	0.54 (0.1)	0.54 (0.1)
P3	0.56 (0.11)	0.67 (0.17)	0.45 (0.15)	0.56 (0.1)	0.59 (0.16)	0.56 (0.11)
P4	0.50 (0.06)	0.51 (0.13)	0.49 (0.09)	0.50 (0.06)	0.50 (0.07)	0.50 (0.06)
O1	0.53 (0.08)	0.53 (0.08)	0.53 (0.17)	0.55 (0.08)	0.51 (0.1)	0.53 (0.08)
O2	0.56 (0.05)	0.56 (0.14)	0.56 (0.08)	0.57 (0.04)	0.57 (0.08)	0.56 (0.05)
F7	0.54 (0.06)	0.57 (0.07)	0.51 (0.09)	0.55 (0.06)	0.53 (0.06)	0.54 (0.06)
F8	0.57 (0.04)	0.64 (0.15)	0.51 (0.09)	0.57 (0.03)	0.59 (0.07)	0.57 (0.04)
T3	0.50 (0.14)	0.49 (0.15)	0.51 (0.21)	0.52 (0.14)	0.48 (0.16)	0.50 (0.14)
T4	0.55 (0.06)	0.61 (0.08)	0.48 (0.1)	0.55 (0.06)	0.55 (0.07)	0.55 (0.06)
T5	0.54 (0.07)	0.56 (0.16)	0.52 (0.07)	0.53 (0.07)	0.55 (0.07)	0.54 (0.06)
T6	0.46 (0.03)	0.49 (0.1)	0.42 (0.05)	0.46 (0.03)	0.45 (0.03)	0.46 (0.03)
A1	0.60 (0.11)	0.63 (0.15)	0.58 (0.09)	0.60 (0.1)	0.61 (0.13)	0.60 (0.11)
A2	0.56 (0.06)	0.57 (0.21)	0.55 (0.12)	0.55 (0.06)	0.58 (0.08)	0.56 (0.05)
FZ	0.45 (0.1)	0.53 (0.09)	0.36 (0.2)	0.47 (0.08)	0.40 (0.15)	0.45 (0.11)
CZ	0.58 (0.06)	0.61 (0.15)	0.55 (0.1)	0.58 (0.05)	0.59 (0.08)	0.58 (0.06)
PZ	0.49 (0.04)	0.59 (0.14)	0.40 (0.07)	0.50 (0.03)	0.50 (0.08)	0.49 (0.04)

Reported are mean (SD) across 5 cross-validation folds (80:20 proportion for training and test sets).

PPV, positive predictive value; NPV, negative predictive value; AUC, area under the curve.

### Control and *post hoc* analyses results

#### Study 1

Control analyses showed that poor classification performance was not dependent on the proportion of data included in the training and test sets, nor on the specific 20 s segment selected, nor on specific feature sets (Supplementary Tables 1–3). *Post hoc* independent *t*-test analyses revealed no statistically significant differences between groups in PAF or theta power at any electrode location (Supplementary Fig. 3; Supplementary Table 4). Classification accuracy was poor also for delta, alpha and beta power (Supplementary Table 5), with only a trend towards significant differences on *post hoc t*-test in some channels along alpha and beta power (Supplementary Figs 4–6; Supplementary Table 6).

#### Study 2

Poor classification performance was not dependent on the proportion of training data nor on the 20 s segment selected (Supplementary Tables 13–16). The features that were most commonly selected (>6 times) across folds and across channels were highly variable across different random segments, with only two features being selected commonly across multiple segments (HT_HypothesisTest_ztest and SB_BinaryStats_iqr.meanstretchdiff; Supplementary Tables 17 and 18). *Post hoc* analyses confirmed that no performance improvement was possible by implementing alternative feature selection methods, alternative classifiers, by simplifying the feature set or the model implemented (Supplementary Section 3 and Supplementary Tables 19–25). Results of the intra-patient feature stability analysis indicated that informative features were moderately stable over the course of the same EEG recording session. In half of the predictions tested, features identified as informative in one 20 s segment remained predictive in the subgroup of patients with detected epileptiform abnormalities (67–83% accuracy) when performance was measured in two different 20 s segments; however, diagnostic class prediction was poor at the whole group level (49–57% accuracy; Supplementary Table 23).

### Subgroup analyses results

#### Study 1

Classification performance was comparable for people with and without diagnostic video-EEG confirmation, and for people with different epilepsy types (Table 4). Classification performance was poor both for those with an overall normal EEG examination outcome and for those with an EEG classified as abnormal with non-specific findings. However, 70% accuracy was achieved for the subgroup of people whose visually normal EEG segment was sampled from recordings that subsequently contained epilepsy-specific abnormalities (71% sensitivity; 50% specificity; Table 4).

**Table 4:** Results of subgroup analyses

	Accuracy	Sensitivity	Specificity	PPV	NPV	AUC
vEEG confirmation						
Yes (*n* = 101)	0.45	0.60	0.34	0.41	0.54	0.47
No (*n* = 47)	0.53	0.53	0.53	0.70	0.35	0.53
Epilepsy type						
Focal (*n* = 33)	0.44	0.57	0.38	0.29	0.66	0.47
Generalized (*n* = 20)	0.43	0.60	0.38	0.21	0.77	0.49
Unclassified (*n* = 22)	0.42	0.54	0.38	0.21	0.73	0.46
Overall EEG outcome						
Normal (*n* = 79)	0.40	0.36	0.42	0.19	0.63	0.39
Abnormal, non-specific (*n* = 39)	0.46	0.60	0.21	0.58	0.23	0.40
Abnormal, epilepsy-specific (*n* = 30)	0.70	0.71	0.50	0.95	0.11	0.61

Displayed are classification indices for different subgroups of patients based on the true and predicted scores across five cross-validation test sets.

vEEG, video-EEG; PPV, positive predictive value; NPV, negative predictive value; AUC, area under the curve.

To further explore this finding, independent *t*-test analyses were performed *post hoc* comparing people with epilepsy for whom epilepsy-specific abnormalities were captured (total *n* = 28; focal *n* = 16; generalized *n* = 10; unclassified *n* = 2) and people with PNES with a normal EEG examination (*n* = 57). This mimics a case–control sampling strategy whereby people with an established diagnosis are compared to a control group with normal EEG, which is known to exaggerate differences.^45-47^ When these groups were considered, a trend towards significantly higher theta power was observed in the epilepsy group over most of the channels (Supplementary Table 7, Supplementary Fig. 7).

Subgroup analyses for other frequency bands are in Supplementary Tables 8–11 and Supplementary Figs 8–10; for the alpha band, these show similar findings to the theta band findings described above.

#### Study 2

In five of the channels (Pz, Cz, F8, P3, T4), 67% to 77% accuracy was achieved for the subgroup of people whose visually normal EEG segment was sampled from recordings that subsequently contained epilepsy-specific abnormalities (Supplementary Table 26). 69% accuracy was achieved in C3 for the subgroup of people whose visually normal EEG segment was sampled from recordings that subsequently contained non-specific abnormalities; this included a mixture of people with epilepsy and people with PNES. Classification performance was poor for all other subgroups tested across all channels (Supplementary Table 26). Subgroup analyses for random segments 2 and 3 showed similar results (random segment 2: 67–73% accuracy in Cz, Fp2 and C4 for the subgroup with epilepsy-specific abnormalities captured, 72–74% accuracy in T6 and P4 for the subgroup with non-specific abnormalities captured; random segment 3: 69–74% accuracy in F7, Fp2 and A1 for the subgroup with epilepsy-specific abnormalities captured; 67–70% accuracy in A2 and C3 for the subgroup with non-specific abnormalities captured).

## Discussion

Two original diagnostic accuracy studies were conducted that sought to determine whether resting-state EEG markers identified through our recent systematic review (theta power and PAF)^3^ have the potential to aid in the classification problem of distinguishing epilepsy and PNES (Study 1), and whether any other markers showing good correlation with the diagnostic labels could be identified in a data-driven fashion from a large, multidisciplinary feature set (Study 2). The visually normal segments of resting-state EEGs of 148 medication-naïve people were analysed; groups were matched by age and gender.

### Study 1

Results of the first study indicated that measures of theta power and PAF predict patients’ diagnoses no better than chance (mean accuracy: 48%) and have therefore poor clinical validity for the diagnostic classification of PNES and epilepsy. Control analyses demonstrated that results were generalizable across different EEG segments and training-test set proportions.

To demonstrate applicability of our model’s results to different patient subgroups, subgroup analyses were performed. Prediction accuracy was relatively higher (70%) for the subgroup of patients who had epilepsy-specific abnormalities captured elsewhere in the course of the EEG recording. A possible explanation is that, for this patient subgroup, our model may be picking up features associated with IEDs that are not detectable on visual inspection. This would be in line with previous evidence showing that the magnitude of resting-state EEG alterations is dependent on the temporal proximity to epileptiform abnormalities.^48^

An ability to predict these cases would offer limited advantage to the diagnostic process. Implementing such a model in clinical practice could, however, bring some benefit to ensure that IEDs are not missed during an EEG recording session (e.g. by extending the session if a patient is flagged as likely to show abnormalities), therefore minimizing any diagnostic delays. Concurrent implementation of clinical tools such as meticulous history taking, seizure semiology and pre-test probability would remain essential for the diagnostic decision.

A number of factors can account for the discrepancy between our findings and those reported by previous studies, including differences in sampling strategies, the specific population under study, and the influence of CNS agents such as ASMs.

Regarding differences in sampling strategies, previous studies all implemented a case–control design whereby a group of patients with known diagnosis is compared to a control group without the condition (typically healthy controls).^3^ This sampling method is known to lead to exaggerated estimates of diagnostic accuracy, especially when cases and controls are sampled from different source populations, and does not correspond to clinical practice where the diagnostic question is between two alternative clinical diagnoses.^45-47^ The present study consecutively enrolled all patients suspected of having non-lesional epilepsy or PNES over a specific time period. Only a small subset (24 patients) had to be identified outside the sequential recruitment approach to achieve age and gender matching. Such a selection more closely reflects the population in whom the marker under study would be used to inform the diagnostic decision-making, therefore mitigating sampling bias.

In an attempt to mimic a case–control design and explore the effect of sampling on results, only the ‘more extreme’ cases on our patient spectrum were selected in *post hoc* analyses: people with PNES who had a completely normal EEG examination, and people with epilepsy with epilepsy-specific abnormalities captured elsewhere in the EEG session. Upon comparison, we replicated or approximated to the finding of increased power in epilepsy, in line with previous case–control studies.^3^ However, our results suggest that the measures studied would have poor applicability in clinical practice as they are not discriminatory in the context of a richer variability of data and clinical presentations.

A second factor relates to the specific population under study. Whilst previous research has largely implemented control groups composed of healthy volunteers, this is one of the first studies directly comparing non-lesional epilepsy and PNES cohorts. Since publication of our systematic review,^3^ one additional study reported increased low frequency power in PNES as compared to healthy controls^49^ in line with findings from a previous study,^50^ whilst a third study reported no differences.^51^ It is possible that low frequency power is elevated in PNES, similarly to epilepsy, and this complicates the classification problem.

A third relevant factor is the effect of ASMs. Consumption of certain ASMs is associated with an increase in delta and theta power and/or to a decline in the dominant (alpha) rhythm frequency^52-56-57^. The EEG data analysed in this study were recorded whilst patients were not taking any CNS agent, whilst almost all previous studies reporting alterations in epilepsy included patients who were already on ASM therapy.^3^ Two exceptions were Schmidt *et al*.^58^ and Clemens^59^ who compared unmedicated epilepsy patients to healthy controls. In accordance with our findings, Schmidt *et al*.^58^ showed very poor discriminative ability for a measure of alpha power peak. Clemens^59^ still reported increased power in epilepsy. Future studies are needed to disentangle the relative contribution of ASMs and genetic factors in modulating EEG alterations in non-lesional epilepsy of unknown aetiology.

### Study 2

Results of the second, data-driven study indicated that identifying resting-state EEG markers that show good correlation with the diagnostic label is challenging. A series of features were consistently flagged as informative by multiple feature selection algorithms and under different analytical circumstances. In some EEG channels, a SVM classifier built on selected features predicted the diagnostic label of novel observations with 67–77% accuracy, but only if patients had epilepsy-specific abnormalities captured elsewhere during the EEG recording. The classifier’s ability to predict the diagnosis at the level of the whole sample (including people without epileptiform abnormalities captured) was poor (45–60% accuracy).

Control analyses excluded the possibility that poor performance was due to overfitting, as simplifying the feature set or the model implemented did not improve results. Poor performance was not due to a failure of the SVM classifier to appropriately represent the feature space, as implementing a different supervised classification approach (Random Forest) did not improve results. Subgroup analyses excluded the possibility that other factors might account for the results, such as the absence of video-EEG diagnostic confirmation for some patients, or the inclusion of patients with different epilepsy types. Analyses of two different resting-state EEG segments sampled at a different time during the recording session produced similar results.

Overall, results of Study 2 suggest that the features were selected based on information that was predominantly present in the EEG of people that were in closer proximity to epileptiform abnormalities. The models learnt to utilise this information to solve the classification problem. When presented with unseen data, the classification accuracy was good for the specific subgroup of people with detected epileptiform abnormalities, but poor for the rest of the sample.

Results of the intra-patient feature stability analysis indicated that in half of the cases, features selected at one time remained predictive when applied to segments taken at different times, but there was very low consistency between the exact features selected across different segments. The EEG channels that were most predictive also varied across time. This suggests that informative features were likely related to transient and spatially evolving EEG dynamics, rather than to spatially and temporally stable traits. The most consistently selected feature was the *P*-value of the *z*-test applied to time series. The *z*-test tests the null hypothesis that the data come from a normal distribution with 0 mean and unit variance. *P*-values represent the deviation between the mean of the time series and a zero mean: the greater the deviation, the lower the *P*-value. In time series, such a measure of deviation from normality may provide information on the degree of ‘anomalies’ in the data. It is possible that this, in conjunction with other metrics, was relevant for classifying the subgroup of people that had visibly detectable epileptiform abnormalities elsewhere in the recording session.

The inability of the present study to identify a feature set that is useful to predict the trait ‘diagnostic class’—rather than conceivably transient EEG states dependent on the proximity to epileptiform abnormalities—does not imply that such a feature set does not exist. However, results of this study highlight that features capturing relevant yet transient dynamics from certain patient subgroups might be ‘stronger’ (i.e. contribute to the variance in y to a greater extent, and are therefore more easily detectable) than features containing information on what we think of as stable traits, such as a categorical diagnostic label—and especially in patients where epilepsy has just begun to manifest with seizures.

It is possible that a mismatch exists between the rigid diagnostic constructs which we seek to define due to their practical utility, and the rich reality of neurophysiological presentations, which might lead to an overlap between diagnostic classes due to shared genetic or environmental factors. For example, ∼30% of the sample had a family history of epilepsy in both the epilepsy and PNES cohorts. The possibility of an overlap between diagnostic classifications is supported by the low-dimensional feature visualizations implementing t-Distributed Stochastic Neighbour Embedding (t-SNE; Supplementary Fig. 12). This suggests that the feature space constitutes a single continuum of observations, without noticeable clusters representing distinct diagnostic categories.

### Limitations and applicability

Limitations include the possibility of misdiagnosis for some cases, as epilepsy-specific abnormalities or a typical episode were never captured on video-EEG in 43% of our epilepsy sample and 21% of our PNES sample respectively. However, the PNES patients were reviewed by a specialist consultant neuropsychiatrist who assigned a diagnosis of PNES due to strong clinical features, and further reviewed by the study Principal Investigator (P.S.). Cohorts without video-EEG diagnostic support did not have lower model accuracy in subgroup analyses, suggesting that diagnostic errors, if present, are not noticeably biasing the model. It cannot be excluded that some patients might have concurrent epilepsy and PNES that went undetected.

Due to analytical requirements, people with unconfirmed diagnosis at the time of data analyses were excluded. A proportion of these were ‘difficult-to-diagnose’ cases and it is expected that their inclusion would further complicate the classification task.^60^ Applicability of the findings is also limited to people without major neurological and psychiatric comorbidities. Patient sampling was performed retrospectively; however, this is not associated with an inaccurate estimation of accuracy indices as compared to prospective diagnostic accuracy studies.^45,46^

EEG data were acquired in clinic and analysed in sensor space. Measures of power and peak frequency can be confounded by the intercept or slope of the aperiodic component of the power spectra; analytical solutions have recently emerged to disentangle their contribution.^61^

For Study 2, analyses performed using a synthetic dataset indicated that the ability of feature selection methods to identify informative features decreases as a function of dataset complexity. With small sample size and high feature set size, feature selection methods struggle to reliably find a feature set with low error from which a good classifier can be designed.^62,63^ We acknowledge the possibility that some of the identified features might be related to the outcome variable spuriously. Increasing the sample size would improve the reliability of feature selection algorithms.

### Future directions

Expanding the length of the EEG segments analysed (e.g. to 5–10 minutes) might decrease the influence of transient dynamics on the features extracted; this could improve their temporal stability (which in the present study was only observed for the subgroup of patients with detected epileptiform abnormalities) and maximize the chances of extrapolating trait-related information. If diagnosis-related information is present in the resting-state EEG of undiagnosed cohorts, this is likely to be more subtle than information related to proximity to epileptiform abnormalities. Analyses could be repeated excluding people that had epileptiform abnormalities detected elsewhere during the recording session. However, increasing the size of study samples should be attempted to account for these exclusions and to address the large variability of neurophysiological presentations observed across the diagnostic classes. This study explored the predictive potential of univariate features, i.e. features derived from individual channels. It is possible that diagnostically relevant information is contained in multivariate features representing the relationship between channels. Relevant information might also be present in recordings taken during other states such as sleep or during task execution and these should be explored in future.

## Conclusions

Two diagnostic accuracy studies were run on visually normal segments of resting-state EEG recordings.

Study 1 investigated the ability of pre-selected markers to distinguish between a diagnosis of epilepsy and a diagnosis of PNES. Contrary to expectations, results indicate that measures of theta power and peak alpha frequency have limited clinical validity as they predict patients’ diagnoses at a level no better than chance (mean accuracy: 48%). Factors that could account for the discrepancy with results of previous studies include sampling strategies, the specific population under study, and the influence of ASMs.

In Study 2, a data-driven exploration was carried out on a large number of features. It was not possible to identify a feature set that predicted people’s diagnosis better than chance. Different feature sets were identified that were predictive of the diagnosis for a subgroup of people who had epileptiform abnormalities detected elsewhere in the course of the EEG recording session. This suggests that EEG markers containing information on the proximity to epileptiform abnormalities contribute to the variance in the outcome variable (diagnosis) to a greater extent than any markers associated to diagnostic class itself. It remains possible that a good feature set exists that is related to diagnostic class. Investments in sample size and innovation in study design are promising determinants of advances in the field.

## Supplementary Material

fcad330_Supplementary_Data
