# Supplementary material for: Limited clinical validity of univariate resting-state EEG markers for classifying seizure disorders
Source: Brain Commun. 2023 Nov 30;5(6):fcad330. doi: 10.1093/braincomms/fcad330 (PMC10724050; doi:10.1093/braincomms/fcad330)
Supplement: fcad330_Supplementary_Data [file fcad330_supplementary_data.pdf]

***Limited clinical validity of univariate resting-state EEG markers for classifying seizure disorders – Brain Communications* manuscript Supplementary Material**

Table of Contents

Section 1: Patient identification strategy and patient characteristics......................................5

Supplementary Figure 1. Retrospective patient identification flowchart....................................6

Supplementary Figure 2. Age distribution for the 5-year range matched cohorts 7

Section 2: Supplementary Material for Study 1 8

Supplementary Table 1. Control analyses on different proportions of training and test data......8

Supplementary Table 2. Control analyses on two different randomly sampled segments (80:20 proportion)....8

Supplementary Table 3. Control analysis on different feature sets. 9

Supplementary Figure 3. Groupwise distribution of log-power or frequency for each predictor variable 9

Supplementary Table 4. Post-hoc independent sample t-test for PAF and theta power at each electrode location....9

Supplementary Table 5. Classification performance for delta, alpha and beta power in 21 channels for one randomly sampled EEG segment. 10

Supplementary Figure 4. Group differences in delta power for the whole sample of people with epilepsy and people with PNES...10

Supplementary Figure 5. Group differences in alpha power for the whole sample of people with epilepsy and people with PNES...11

Supplementary Figure 6. Group differences in beta power for the whole sample of people with epilepsy and people with PNES.. 11

Supplementary Table 6. Post-hoc independent sample t-test for power at each electrode location along the delta, alpha and beta frequency bands. 12

Supplementary Figure 7. Group differences in PAF and theta power between people with PNES with normal EEG (n=57) and people with epilepsy with observed epileptiform EEG abnormalities later in the recordings (n=28). 13

Supplementary Table 7. Post-hoc independent sample t-test results for PAF and theta power, when comparing people with PNES with normal EEG (n=57) and people with epilepsy with observed epileptiform EEG abnormalities (n=28). 14

Supplementary Table 8. Results of subset analyses for Delta power. 15

Supplementary Table 9. Results of subset analyses for Alpha power. 15

Supplementary Table 10. Results of subset analyses for Beta power. 16

Supplementary Table 11. Post-hoc independent sample t-test comparing people with PNES with normal EEG and people with epilepsy with detected epileptiform abnormalities. 16

Supplementary Figure 8. Group differences in Delta power between people with PNES with normal EEG (n=57) and people with epilepsy with detected epileptiform abnormalities (n=28) 18

Supplementary Figure 9. Group differences in Alpha power between people with PNES with normal EEG (n=57) and people with epilepsy with detected epileptiform abnormalities (n=28) 18

Supplementary Figure 10. Group differences in Beta power between people with PNES with normal EEG (n=57) and people with epilepsy with detected epileptiform abnormalities (n=28) 19

Section 3: Supplementary Material for Study 2 20

Supplementary Figure 11. Machine Learning pipeline implemented. 21

Supplementary Table 12. Most commonly selected features in random segment 1. 25

Supplementary Table 13. Control analysis with 70:30 split proportion 27

Supplementary Table 14. Control analysis with 90:10 split proportion. 27

Supplementary Table 15. Control analyses on random segment 2. 28

Supplementary Table 16. Control analyses on random segment 3. 30

Supplementary Table 17. Control analyses on random segment 2; most commonly selected features across different channels and different folds. 30

Supplementary Table 18. Control analyses on random segment 3; most commonly selected features across different channels and different folds. 31

Supplementary Table 19. Post-hoc analyses on toy dataset to ensure the correctness of the code and to explore the classification performance of different feature selection methods with increasing dataset complexity. 32

Supplementary Table 20. Post-hoc analyses implementing Recursive Feature Elimination as a feature selection method (instead of mRMR) on the first random segment. 34

Supplementary Table 21. Post-hoc analysis with reduced feature number on the first random segment. 35

Supplementary Table 22. Number of selected features as a function of training set size. 36

Supplementary Table 23. Post-hoc analysis to assess intra-patient feature stability across different random segments. 37

Supplementary Table 24. Post-hoc Linear Discriminant Analysis on the first random segment. 38

Supplementary Table 25. Post-hoc Random Forest analysis on the first random segment. 39

Supplementary Figure 12. Two-dimensional t-SNE visualisations of the feature space structure for three sample channels. 40

Supplementary Table 26. Subset analyses results for selected features in 21 channels for the first randomly sampled EEG segment... 41

Section 4: SVM code for Study 1 47

Section 5: SVM code for Study 2 55

Section 6: Supplementary Material References 63

Section 1: Patient identification strategy and patient characteristics

The retrospective patient identification flowchart is presented in Supplementary Figure 1.

To identify eligible patients, the following patient lists were retrospectively and consecutively screened: the Activation Clinic list between March 2012 and July 2021, the First Fit nurse-led clinic list between December 2020 and August 2021, and the General Neurology "Hot" Clinic between 2019 and 2020. The General Neurology clinic list between 2015 and 2018 was also partially screened at random. For each patient on these specialist clinic lists, clinical notes relevant to their first presentation with suspected seizure disorder were retrieved and assessed against inclusion/exclusion criteria; for some patients, these might have been as old as, e.g., 2007 (year of the oldest EEG examination utilised for this study). For other patients, these might have been recent notes for a recent seizure onset.

After the first 161 eligible patients were identified, an inspection for matching was carried out. 53 patients with an established diagnosis of epilepsy naturally matched 53 patients with a diagnosis of PNES by gender and age at the time of EEG (with a 5 Year Range age matching rule). 18 patients with PNES of female gender and 6 patients with epilepsy of male gender remained unmatched. 31 patients had uncertain diagnosis and were left unmatched at this stage.

To achieve age and gender matching for the 18 females with PNES and 6 males with epilepsy, we performed a targeted search using Cogstack, an information retrieval and text extraction platform from clinical notes at King's College Hospital. A Cogstack search was run to generate a list of female patients who had an EEG examination at KCH and were aged between 18 and 45 years at the time of EEG, and a list of male patients who were aged between 18 and 63 years at the time of EEG. To facilitate the identification of cases, we further instructed the search to only return results if the EEG letter included the keyword "seizure" (for females) or "non-epileptic seizures" or synonyms (for males) and if the word "medications" was in close proximity to the word "nil". Records were screened consecutively from 2020 backwards up to 2018. 18 eligible females with a diagnosis of idiopathic epilepsy and 6 males with a diagnosis of PNES were therefore identified to match the existing patients by age and gender (with a 5 Year Range matching rule).

After targeted matching and exclusion of people for whom the EEG data could not be retrieved, a total of 146 eligible patients with medication-free EEG and ascertained diagnosis were

included; these were 73 epilepsy patients and 73 PNES patients, matched by age and gender. By the time of data analysis, two additional patients from the uncertain diagnosis group eventually received a diagnosis of epilepsy and were therefore included in the analyses in the absence of a PNES matched counterpart. Age distribution and gender proportion for the two cohorts are depicted in Supplementary Figure 2.

Supplementary Figure 1. Retrospective patient identification flowchart

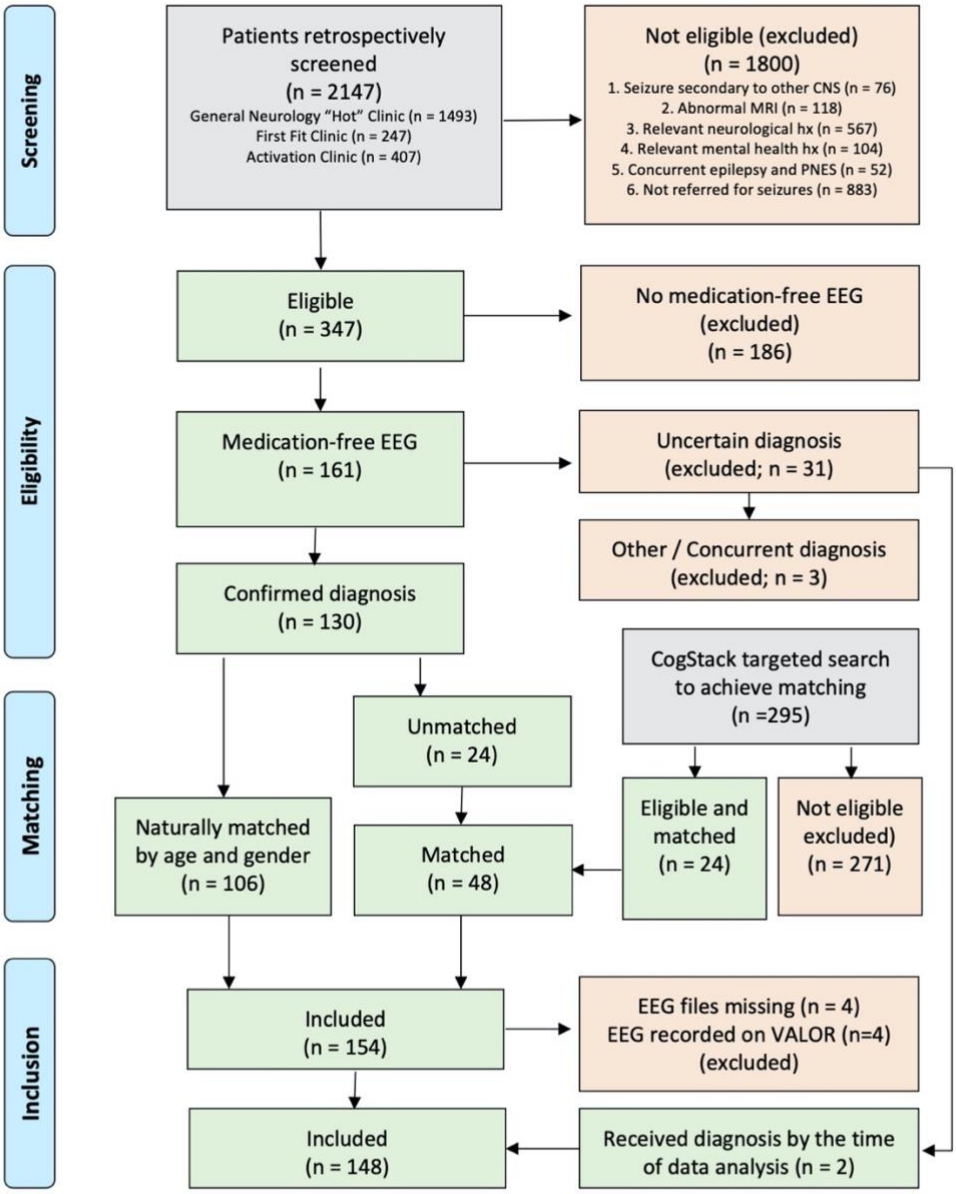

Supplementary Figure 2. Age distribution for the 5-year range matched cohorts

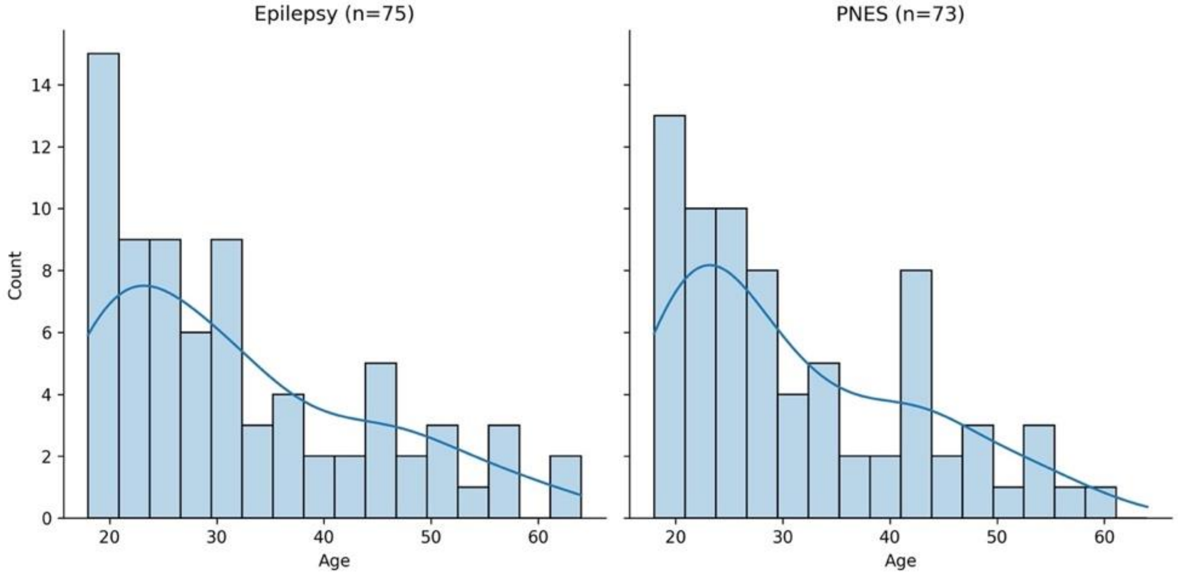

Details on inclusion and exclusion criteria: Exclusion criteria were acute symptomatic seizures, abnormal CT/MRI, eventual diagnosis of concurrent epilepsy and PNES, relevant history of other neurological, neurodevelopmental, or severe mental health disorders.

With regards to relevant history of other neurological disorders, this refers to disorders such as brain injury, ischaemic events, brain tumours, encephalitis, idiopathic intracranial hypertension, neural malformations, moderate to severe small vessel disease, cerebral hypoxia, neurodegenerative disorders, cerebral palsy, severe sleep disorders, brain surgery. We also excluded people with concurrent atrial fibrillation.

With regards to neurodevelopmental disorders, we excluded people with concurrent moderate to severe autism spectrum disorder, ADHD, or learning disabilities.

With regards to severe mental health disorders, we excluded people with active and severe mental health disorder such as major depressive disorder, bipolar disorder, active suicidal ideation, psychosis, schizophrenia, obsessive-compulsive disorder, addiction, multiple personality disorders, PTSD.

Examples of concurrent disorders that did not lead to participant exclusion are migraines, headaches, mild to moderate anxiety or depression, borderline personality disorder, mental health illness considered treated or resolved (e.g., past PTSD, depression, or self-harm), mild autism spectrum disorder, mild ADHD, dyslexia.

Section 2: Supplementary Material for Study 1

Model fitting methods: technical details

To test whether the extracted features (theta power in 21 channels and global PAF) could predict the binary diagnostic class (epilepsy / PNES), a Support Vector Machine (SVM) model was fit to the data[1] using the scikit-learn software in Python v3.10.4.[2] The whole dataset was split (at the patient level) into a training set (80% of the patients) and a test set (20% of the patients), stratifying by diagnosis (Figure 2). Features in the training set were normalised to zero mean and unit variance. Features in the test set were transformed using the same normalisation parameters as the training set. A radial basis function (RBF) was implemented.[3] To find the optimal hyperparameters values (C and γ), a model was built and evaluated for a grid search over a range of plausible parameters pre-specified as follows: C: [0.01, 0.1, 1, 10, 100, 1000, 10000], and γ: [1, 0.1, 0.01, 0.001, 0.0001, 0.0001, 0.00001]. The decision threshold for test positivity was set to 0.5. The optimal kernel parameter combination was identified through a 5-fold cross-validation procedure, and a final model was fit on the whole training set using the optimal parameters. Performance of the final model was measured on the test set. This process was repeated five times by implementing a 5-fold cross validation procedure on the training and test set. Classification performance for each test set was then averaged over the five outer folds to obtain an estimate of the average performance for the models. This is reported in results tables.

Control and post-hoc analyses results

Supplementary Table 1. Control analyses on different proportions of training and test data. Reported are mean (SD) across 5 cross validation folds.

Proportion	Accuracy	Sensitivity	Specificity	PPV	NPV	AUC
90:10	0.460 (0.112)	0.550 (0.234)	0.362 (0.225)	0.467 (0.107)	0.396 (0.188)	0.456 (0.112)
70:30	0.467 (0.046)	0.600 (0.234)	0.338 (0.182)	0.473 (0.040)	0.366 (0.192)	0.469 (0.047)
Abbreviations: PPV = Positive Predictive Value; NPV = Negative Predictive Value; AUC = Area Under the Curve.						

Supplementary Table 2. Control analyses on two different randomly sampled segments (80:20 proportion). Reported are mean (SD) across 5 cross validation folds.

20s EEG Segment	Accuracy	Sensitivity	Specificity	PPV	NPV	AUC

Random segment 2	0.541 (0.088)	0.573 (0.108)	0.505 (0.171)	0.553 (0.087)	0.527 (0.097)	0.539 (0.087)
Random segment 3	0.521 (0.082)	0.613 (0.136)	0.426 (0.151)	0.524 (0.066)	0.508 (0.132)	0.520 (0.081)
Abbreviations: PPV = Positive Predictive Value; NPV = Negative Predictive Value; AUC = Area Under the Curve.						

Supplementary Table 3. Control analysis on different feature sets. Reported is the classification performance separately for theta power in 21 channels and for Peak Alpha Frequency (PAF). Reported are mean (SD) across 5 cross validation folds (80:20 proportion for training and test sets).

Predictors	Accuracy	Sensitivity	Specificity	PPV	NPV	AUC
Theta (21 channels) only	0.487 (0.082)	0.493 (0.116)	0.478 (0.102)	0.490 (0.083)	0.479 (0.089)	0.486 (0.082)
PAF only	0.528 (0.054)	0.787 (0.129)	0.263 (0.136)	0.523 (0.041)	0.443 (0.239)	0.525 (0.050)
Abbreviations: PPV = Positive Predictive Value; NPV = Negative Predictive Value; AUC = Area Under the Curve.						

Supplementary Figure 3. Groupwise distribution of log-power or frequency for each predictor variable. See Supplementary Table 4 for corresponding numerical values.

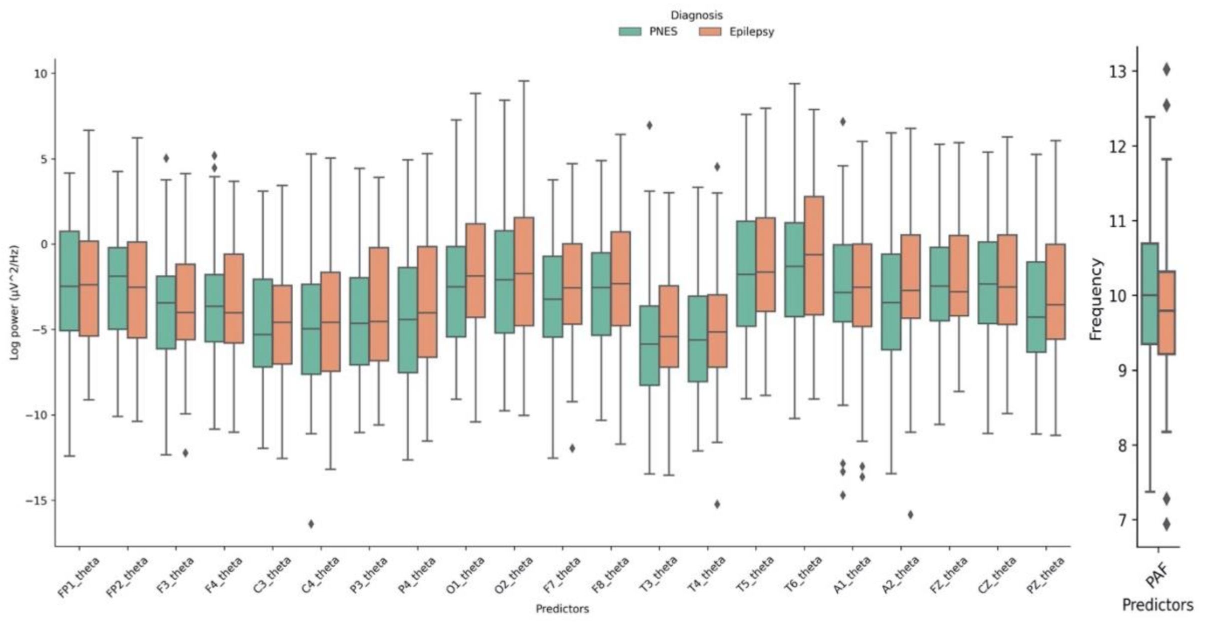

Supplementary Table 4. Post-hoc independent sample t-test for PAF and theta power at each electrode location. P-values are uncorrected for multiple comparisons. To apply Bonferroni correction, compare against α = 0.002.

Predictor	Epilepsy mean (SD)	PNES mean (SD)	t statistic	p-value

FP1	-2.48 (3.69)	-2.8 (3.83)	0.52	0.602
FP2	-2.55 (3.64)	-2.56 (3.5)	0.03	0.977
F3	-3.44 (3.37)	-3.79 (3.57)	0.63	0.531
F4	-3.35 (3.2)	-3.5 (3.44)	0.27	0.785
C3	-4.48 (3.57)	-4.65 (3.61)	0.29	0.77
C4	-4.52 (4.02)	-4.64 (3.9)	0.19	0.853
P3	-3.75 (3.81)	-4.51 (3.83)	1.21	0.229
P4	-3.68 (3.92)	-4.2 (4.38)	0.76	0.45
O1	-1.39 (3.96)	-2.23 (3.89)	1.3	0.195
O2	-1.33 (4.02)	-2.01 (4.19)	1.02	0.309
F7	-2.61 (3.76)	-3.24 (3.81)	1.02	0.311
F8	-2.34 (3.73)	-2.73 (3.33)	0.66	0.512
T3	-4.91 (3.54)	-5.53 (3.8)	1.03	0.306
T4	-4.88 (3.54)	-5.45 (3.5)	0.99	0.326
T5	-1.1 (3.97)	-1.4 (4.32)	0.45	0.656
T6	-0.69 (4.31)	-1.23 (4.83)	0.72	0.471
A1	-2.72 (4.31)	-2.77 (4.12)	0.07	0.945
A2	-2.12 (4.18)	-3.33 (4.34)	1.73	0.086
FZ	-2.13 (3.23)	-2.16 (3.37)	0.06	0.955
CZ	-2.25 (3.86)	-2.38 (3.6)	0.21	0.831
PZ	-2.98 (4.09)	-3.83 (4.12)	1.26	0.209
PAF	9.83 (1.07)	9.98 (0.98)	-0.86	0.391

Supplementary Table 5. Classification performance for delta, alpha and beta power in 21 channels for one randomly sampled EEG segment. Reported are mean (SD) across 5 cross validation folds (80:20 proportion for training and test sets).

Predictor (21 channels)	Accuracy	Sensitivity	Specificity	PPV	NPV	AUC
Delta power	0.466 (0.06)	0.600 (0.36)	0.339 (0.32)	0.461 (0.10)	0.448 (0.32)	0.470 (0.06)
Alpha power	0.533 (0.11)	0.707 (0.20)	0.354 (0.11)	0.520 (0.09)	0.574 (0.18)	0.530 (0.12)
Beta power	0.534 (0.08)	0.587 (0.13)	0.477 (0.10)	0.532 (0.08)	0.531 (0.09)	0.532 (0.08)

Supplementary Figure 4. Group differences in delta power for the whole sample of people with epilepsy and people with PNES. See Supplementary Table 6 for corresponding numerical values.

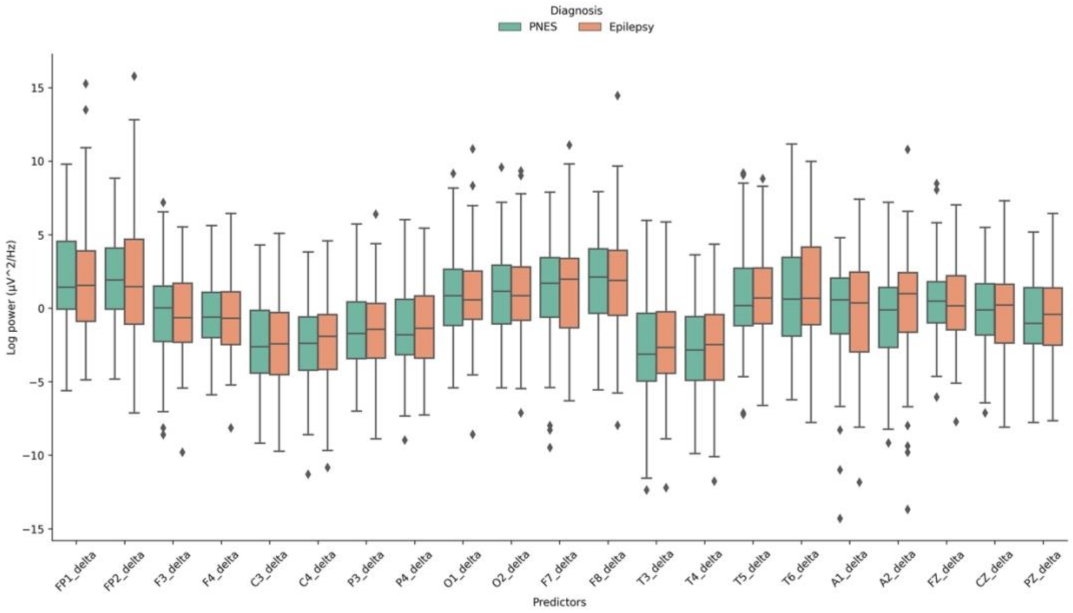

Supplementary Figure 5. Group differences in alpha power for the whole sample of people with epilepsy and people with PNES. See Supplementary Table 6 for corresponding numerical values.

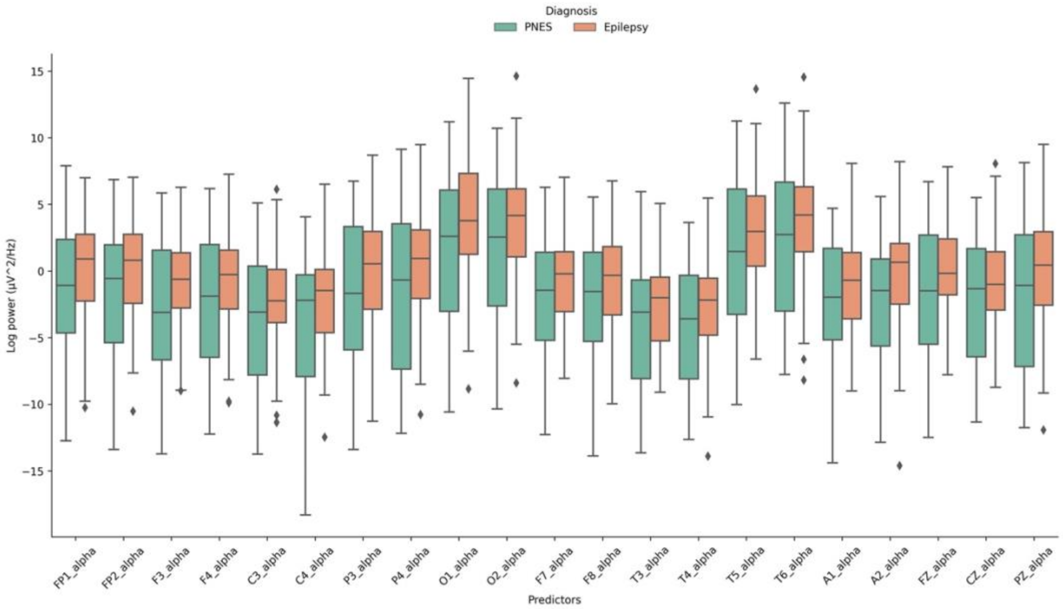

Supplementary Figure 6. Group differences in beta power for the whole sample of people with epilepsy and people with PNES. See Supplementary Table 6 for corresponding numerical values.

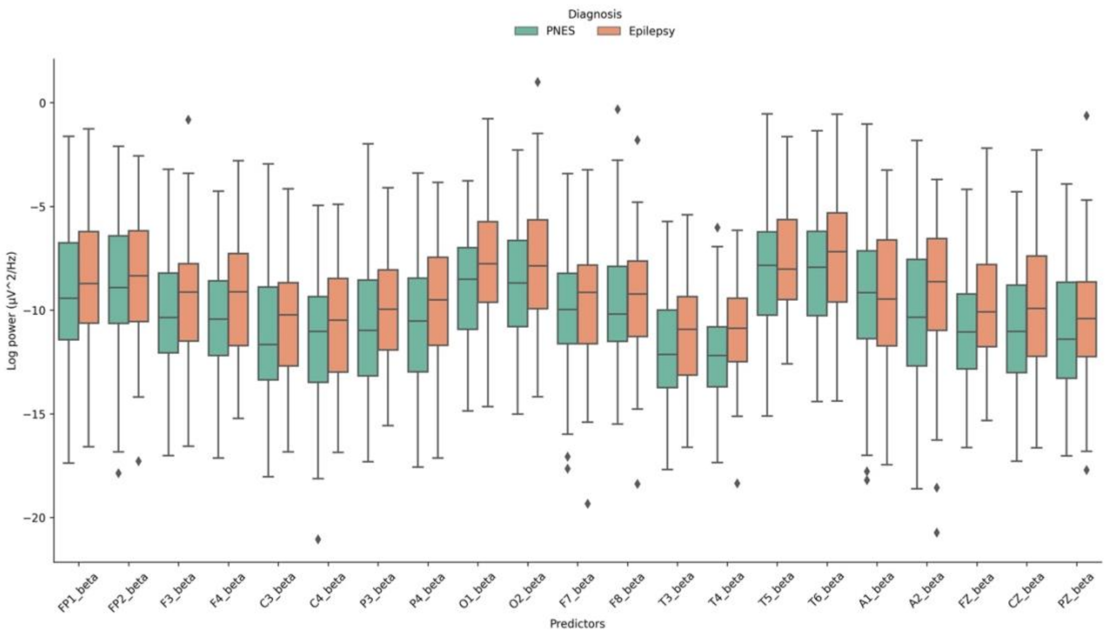

Supplementary Table 6. Post-hoc independent sample t-test for power at each electrode location along the delta, alpha and beta frequency bands. p-values are uncorrected for multiple comparisons. To apply Bonferroni correction, compare against α = 0.002.

	**Delta power**				**Alpha power**				**Beta power**			
	**Epilepsy mean (SD)**	**PNES mean (SD)**	**t**	**p**	**Epilepsy mean (SD)**	**PNES mean (SD)**	**t**	**p**	**Epilepsy mean (SD)**	**PNES mean (SD)**	**t**	**p**
**FP1**	1.93 (3.99)	1.91 (3.52)	0.02	0.98	-0.02 (3.75)	-1.57 (4.82)	2.19	0.03	-8.44 (3.23)	-9.34 (3.38)	1.66	0.099
**FP2**	1.78 (4.21)	1.94 (2.95)	-0.27	0.785	-0.12 (3.87)	-1.43 (4.8)	1.84	0.068	-8.46 (3.16)	-8.97 (3.23)	0.98	0.328
**F3**	-0.52 (2.9)	-0.45 (3.07)	-0.15	0.885	-0.85 (3.41)	-2.41 (4.7)	2.31	0.022	-9.52 (2.89)	-10.39 (3.0)	1.8	0.074
**F4**	-0.6 (2.5)	-0.46 (2.59)	-0.32	0.75	-0.84 (3.68)	-2.3 (4.82)	2.06	0.041	-9.34 (2.86)	-10.24 (2.79)	1.94	0.055
**C3**	-2.44 (2.79)	-2.52 (2.77)	0.17	0.865	-2.24 (3.65)	-3.57 (4.87)	1.88	0.063	-10.66 (2.93)	-11.07 (3.26)	0.8	0.423
**C4**	-2.4 (3.02)	-2.43 (2.93)	0.06	0.955	-2.25 (3.82)	-3.63 (4.91)	1.92	0.057	-10.65 (2.86)	-11.35 (3.37)	1.36	0.176
**P3**	-1.34 (2.79)	-1.35 (2.97)	0.03	0.979	0.17 (4.39)	-1.61 (5.54)	2.18	0.031	-9.87 (2.78)	-10.71 (3.15)	1.73	0.085
**P4**	-1.07 (2.95)	-1.31 (3.27)	0.48	0.635	0.16 (4.27)	-1.39 (5.81)	1.85	0.067	-9.82 (2.89)	-10.74 (3.23)	1.82	0.071

**O1**	0.96 (3.1)	0.94 (3.11)	0.04	0.971	3.78 (4.5)	1.74 (5.41)	2.5	0.014	-7.69 (2.96)	-8.78 (2.83)	2.28	0.024
**O2**	1.17 (3.07)	1.0 (3.03)	0.33	0.744	3.68 (4.37)	2.05 (5.34)	2.04	0.043	-7.78 (3.16)	-8.71 (2.98)	1.85	0.066
**F7**	1.47 (3.59)	1.09 (3.5)	0.66	0.513	-0.74 (3.41)	-2.42 (4.8)	2.45	0.015	-9.59 (2.91)	-10.26 (2.79)	1.44	0.152
**F8**	1.72 (3.9)	1.64 (3.29)	0.14	0.891	-0.77 (3.73)	-2.17 (4.37)	2.11	0.037	-9.44 (2.7)	-9.78 (3.05)	0.71	0.477
**T3**	-2.54 (3.23)	-2.81 (3.25)	0.5	0.618	-2.52 (3.32)	-4.21 (4.62)	2.55	0.012	-11.06 (2.45)	-11.95 (2.68)	2.1	0.037
**T4**	-2.7 (3.13)	-2.88 (3.04)	0.34	0.733	-2.61 (3.49)	-4.2 (4.24)	2.49	0.014	-11.02 (2.23)	-12.11 (2.37)	2.9	0.004
**T5**	1.01 (3.24)	0.86 (3.55)	0.28	0.782	2.87 (4.19)	1.48 (5.3)	1.77	0.079	-7.63 (2.54)	-8.2 (2.87)	1.27	0.206
**T6**	1.3 (3.6)	1.16 (4.03)	0.22	0.823	3.64 (4.46)	2.18 (5.56)	1.76	0.08	-7.4 (3.06)	-8.11 (3.18)	1.4	0.164
**A1**	-0.38 (3.69)	-0.29 (3.64)	-0.15	0.884	-0.79 (3.77)	-2.13 (4.44)	1.98	0.05	-9.38 (3.6)	-9.47 (3.61)	0.15	0.879
**A2**	0.12 (3.87)	-0.92 (3.48)	1.72	0.087	-0.2 (3.99)	-2.35 (4.66)	3.02	0.003	-9.23 (3.43)	-10.31 (3.74)	1.83	0.069
**FZ**	0.21 (2.79)	0.5 (2.75)	-0.64	0.524	0.01 (3.55)	-1.53 (4.73)	2.24	0.027	-9.85 (2.6)	-10.91 (2.7)	2.45	0.016
**CZ**	-0.25 (3.02)	-0.18 (2.83)	-0.15	0.881	-0.94 (3.64)	-2.32 (4.75)	1.99	0.048	-9.81 (3.28)	-10.91 (3.03)	2.12	0.036
**PZ**	-0.43 (2.84)	-0.57 (2.86)	0.29	0.773	-0.11 (4.22)	-1.74 (5.52)	2.02	0.046	-10.33 (3.08)	-11.16 (3.1)	1.62	0.107

Subset analyses results for theta

Supplementary Figure 7. Group differences in PAF and theta power between people with PNES with normal EEG (n=57) and people with epilepsy with observed epileptiform EEG abnormalities later in the recordings (n=28). Notes: *p<0.05; **p<0.01; ns = not significant. P-values presented here are uncorrected for multiple comparisons. See Supplementary Table 7 for corresponding numerical values.

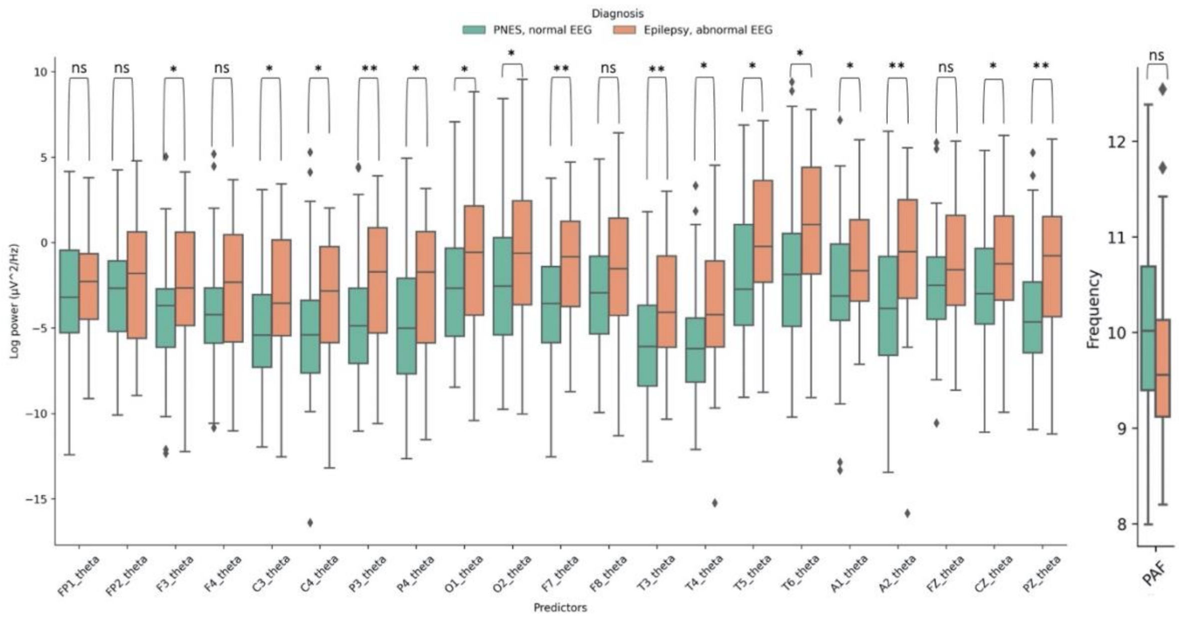

Supplementary Table 7. Post-hoc independent sample t-test results for PAF and theta power, when comparing people with PNES with normal EEG (n=57) and people with epilepsy with observed epileptiform EEG abnormalities (n=28). P-values are uncorrected for multiple comparisons. To apply Bonferroni correction, compare against α = 0.002.

Predictor	Epilepsy mean (SD)	PNES mean (SD)	t statistic	p-value
FP1	-2.28 (3.33)	-3.24 (3.86)	1.13	0.262
FP2	-2.35 (3.79)	-2.98 (3.49)	0.77	0.446
F3	-2.29 (3.55)	-4.23 (3.34)	2.46	0.016
F4	-2.57 (3.77)	-3.91 (3.19)	1.71	0.09
C3	-2.93 (3.87)	-5.06 (3.33)	2.63	0.01
C4	-3.18 (4.05)	-5.07 (3.71)	2.15	0.035
P3	-2.34 (4.18)	-4.91 (3.55)	2.96	0.004
P4	-2.45 (4.08)	-4.71 (4.04)	2.43	0.017
O1	-0.7 (4.5)	-2.58 (3.76)	2.03	0.046
O2	-0.32 (4.58)	-2.43 (4.06)	2.16	0.033
F7	-1.47 (3.66)	-3.74 (3.66)	2.69	0.009
F8	-1.77 (4.34)	-2.85 (3.18)	1.3	0.196
T3	-3.89 (3.81)	-5.96 (3.19)	2.64	0.01
T4	-3.98 (4.35)	-5.92 (3.42)	2.24	0.028
T5	0.15 (4.37)	-1.87 (3.96)	2.14	0.035

T6	0.84 (4.77)	-1.73 (4.68)	2.36	0.021
A1	-1.01 (3.46)	-2.89 (3.87)	2.17	0.033
A2	-0.64 (4.49)	-3.72 (4.39)	3.01	0.003
FZ	-1.24 (3.43)	-2.58 (3.07)	1.83	0.071
CZ	-1.05 (3.97)	-2.73 (3.36)	2.05	0.044
PZ	-1.56 (4.46)	-4.22 (3.88)	2.82	0.006
PAF	9.76 (1.01)	10.05 (0.93)	-1.33	0.188

Subset analyses results for other frequency bands (delta, alpha, beta)

Similarly to what observed for the theta band, the model's prediction based on alpha power was more accurate (70%) for the subset of patients that had epilepsy-specific abnormalities captured later during the EEG recording session (sensitivity 75%; specificity 0%; PPV 91%; Supplementary Table 9). For power along the delta and beta frequency ranges, no strong subset effects were noticed (Supplementary Tables 8 and 10).

Supplementary Table 8. Results of subset analyses for Delta power. Displayed are classification indices for different subsets of patients based on the true and predicted scores across five cross validation test sets.

	Accuracy	Sensitivity	Specificity	PPV	NPV	AUC
**vEEG confirmation**						
Yes (n=101)	0.46	0.60	0.36	0.41	0.55	0.48
No (n=47)	0.47	0.59	0.2	0.61	0.19	0.40
**Epilepsy type**						
Focal (n=33)	0.42	0.64	0.32	0.3	0.66	0.48
Generalised (n=20)	0.38	0.55	0.32	0.18	0.72	0.43
Unclassified (n=22)	0.39	0.59	0.32	0.21	0.72	0.45
**Overall EEG outcome**						
Normal (n=79)	0.40	0.5	0.37	0.23	0.66	0.43
Abnormal, non-specific (n=39)	0.54	0.72	0.21	0.62	0.3	0.47
Abnormal, epilepsy-specific (n=30)	0.53	0.57	0.0	0.89	0.0	0.28

Supplementary Table 9. Results of subset analyses for Alpha power. Displayed are classification indices for different subsets of patients based on the true and predicted scores across five cross validation test sets.

	Accuracy	Sensitivity	Specificity	PPV	NPV	AUC
**vEEG confirmation**						
Yes (n=101)	0.49	0.72	0.33	0.44	0.61	0.52

No (n=47)	0.62	0.69	0.46	0.73	0.41	0.57
**Epilepsy type**						
Focal (n=33)	0.45	0.67	0.35	0.32	0.70	0.51
Generalised (n=20)	0.46	0.85	0.36	0.26	0.90	0.60
Unclassified (n=22)	0.42	0.63	0.35	0.23	0.76	0.50
**Overall EEG outcome**						
Normal (n=79)	0.49	0.73	0.40	0.32	0.79	0.56
Abnormal, non-specific (n=39)	0.49	0.64	0.21	0.59	0.25	0.43
Abnormal, epilepsy-specific (n=30)	0.70	0.75	0.0	0.91	0.0	0.37

Supplementary Table 10. Results of subset analyses for Beta power. Displayed are classification indices for different subsets of patients based on the true and predicted scores across five cross validation test sets.

	Accuracy	Sensitivity	Specificity	PPV	NPV	AUC
**vEEG confirmation**						
Yes (n=101)	0.51	0.56	0.48	0.44	0.59	0.52
No (n=47)	0.57	0.62	0.46	0.71	0.37	0.54
**Epilepsy type**						
Focal (n=33)	0.49	0.51	0.48	0.31	0.69	0.50
Generalised (n=20)	0.50	0.60	0.47	0.24	0.81	0.54
Unclassified (n=22)	0.53	0.68	0.48	0.28	0.83	0.58
**Overall EEG outcome**						
Normal (n=79)	0.47	0.50	0.45	0.26	0.70	0.47
Abnormal, non-specific (n=39)	0.64	0.68	0.57	0.74	0.50	0.62
Abnormal, epilepsy-specific (n=30)	0.56	0.57	0.5	0.94	0.08	0.54

Similarly to what observed for theta power, a sampling effect was noted for alpha power; maximising differences between groups by only considering people with epilepsy with observed epileptiform EEG abnormalities and people with PNES with a completely normal EEG examination resulted in a significant difference in the right mid-temporal electrode (A2), with a strong trend towards significance in all other electrodes (Supplementary Table 11, Supplementary Figure 9). For beta power, a trend towards significance remained for six electrodes (Supplementary Table 11, Supplementary Figure 10). For delta power, no significant group differences were observed at any electrode location when differences between groups were maximised (Supplementary Table 11, Supplementary Figure 8).

Supplementary Table 11. Post-hoc independent sample t-test comparing people with PNES with normal EEG and people with epilepsy with detected epileptiform abnormalities. p-values are uncorrected for multiple comparisons. To apply Bonferroni correction, compare against α = 0.002.

	Delta power				Alpha power				Beta power			
	**Epilepsy mean (SD)**	**PNES mean (SD)**	**t**	**p**	**Epilepsy mean (SD)**	**PNES mean (SD)**	**t**	**p**	**Epilepsy mean (SD)**	**PNES mean (SD)**	**t**	**p**
FP1	1.56 (3.15)	1.59 (3.6)	-0.04	0.966	0.68 (3.7)	-1.87 (5.03)	2.37	0.02	-8.74 (2.99)	-9.39 (3.54)	0.84	0.404
FP2	1.47 (3.96)	1.7 (3.17)	-0.29	0.769	0.54 (4.19)	-1.75 (4.97)	2.09	0.04	-8.91 (3.25)	-9.12 (3.33)	0.27	0.786
F3	0.22 (3.21)	-0.57 (3.19)	1.07	0.289	0.26 (3.67)	-2.65 (4.77)	2.84	0.006	-9.19 (2.5)	-10.5 (2.97)	2.01	0.048
F4	-0.01 (2.87)	-0.58 (2.53)	0.93	0.354	0.26 (4.21)	-2.61 (4.83)	2.68	0.009	-9.13 (2.99)	-10.31 (2.84)	1.77	0.08
C3	-1.81 (3.16)	-2.66 (2.66)	1.31	0.194	-1.01 (4.05)	-3.91 (4.87)	2.72	0.008	-10.22 (2.88)	-11.13 (3.21)	1.27	0.209
C4	-1.67 (3.17)	-2.63 (2.83)	1.4	0.164	-1.25 (4.1)	-3.96 (4.92)	2.51	0.014	-10.17 (2.72)	-11.39 (3.35)	1.67	0.098
P3	-0.35 (3.15)	-1.6 (2.84)	1.85	0.068	1.66 (4.81)	-2.06 (5.57)	3.02	0.003	-9.41 (3.11)	-10.77 (3.15)	1.88	0.064
P4	-0.27 (2.92)	-1.64 (3.13)	1.94	0.056	1.05 (4.39)	-1.78 (5.79)	2.27	0.026	-9.32 (3.01)	-10.83 (3.13)	2.11	0.038
O1	1.39 (3.65)	0.95 (3.2)	0.56	0.575	4.77 (4.87)	1.46 (5.63)	2.66	0.009	-7.62 (3.62)	-8.77 (2.85)	1.59	0.115
O2	1.73 (3.6)	0.96 (3.07)	1.03	0.308	4.56 (4.91)	1.78 (5.52)	2.26	0.026	-7.59 (3.69)	-8.72 (2.85)	1.56	0.122
F7	2.21 (3.4)	0.75 (3.53)	1.81	0.073	0.19 (3.48)	-2.77 (4.97)	2.83	0.006	-9.47 (2.68)	-10.42 (2.63)	1.56	0.123
F8	1.69 (4.04)	1.6 (3.44)	0.11	0.917	-0.04 (4.25)	-2.3 (4.41)	2.25	0.027	-9.61 (2.96)	-9.67 (2.86)	0.08	0.938
T3	-1.94 (3.64)	-2.94 (2.94)	1.36	0.178	-1.82 (3.57)	-4.51 (4.56)	2.73	0.008	-10.81 (2.52)	-11.94 (2.37)	2.04	0.045
T4	-2.43 (3.26)	-3.14 (3.08)	0.98	0.329	-1.98 (4.25)	-4.55 (4.24)	2.63	0.01	-10.91 (2.43)	-12.12 (2.29)	2.24	0.028
T5	1.94 (3.35)	0.58 (3.29)	1.78	0.079	4.0 (4.62)	1.16 (5.39)	2.38	0.019	-7.42 (2.81)	-8.15 (2.9)	1.11	0.269
T6	2.08 (4.05)	0.95 (3.91)	1.23	0.221	4.6 (5.1)	1.88 (5.65)	2.16	0.034	-7.03 (3.42)	-8.05 (3.08)	1.39	0.169
A1	1.09 (2.56)	-0.18 (3.36)	1.77	0.08	0.52 (3.32)	-2.03 (4.53)	2.65	0.01	-8.06 (3.01)	-9.09 (3.6)	1.31	0.193

A2	0.94 (3.9)	-1.04 (3.56)	2.33	0.022	0.94 (4.41)	-2.61 (4.69)	3.34	**0.001***	-8.63 (3.44)	-10.34 (3.74)	2.03	0.045
FZ	1.06 (3.14)	0.37 (2.54)	1.1	0.276	1.25 (3.93)	-1.79 (4.76)	2.92	0.005	-9.5 (2.57)	-10.92 (2.67)	2.34	0.022
CZ	0.51 (3.26)	-0.29 (2.74)	1.19	0.238	0.31 (4.09)	-2.59 (4.68)	2.79	0.006	-9.46 (3.35)	-10.87 (3.04)	1.94	0.055
PZ	0.45 (3.21)	-0.76 (2.84)	1.77	0.081	1.08 (4.71)	-2.05 (5.53)	2.57	0.012	-9.96 (3.38)	-11.25 (3.08)	1.76	0.083
* Significant difference between groups following Bonferroni correction.												

Supplementary Figure 8. Group differences in Delta power between people with PNES with normal EEG (n=57) and people with epilepsy with detected epileptiform abnormalities (n=28). See Supplementary Table 11 for corresponding numerical values.

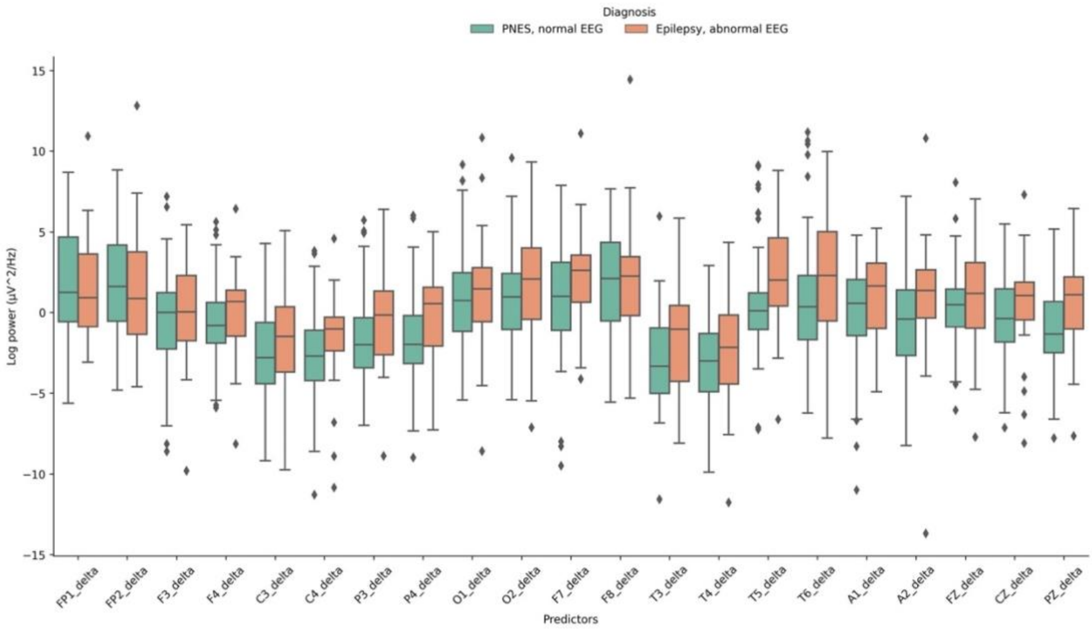

Supplementary Figure 9. Group differences in Alpha power between people with PNES with normal EEG (n=57) and people with epilepsy with detected epileptiform abnormalities (n=28). See

Supplementary Table 11 for corresponding numerical values.

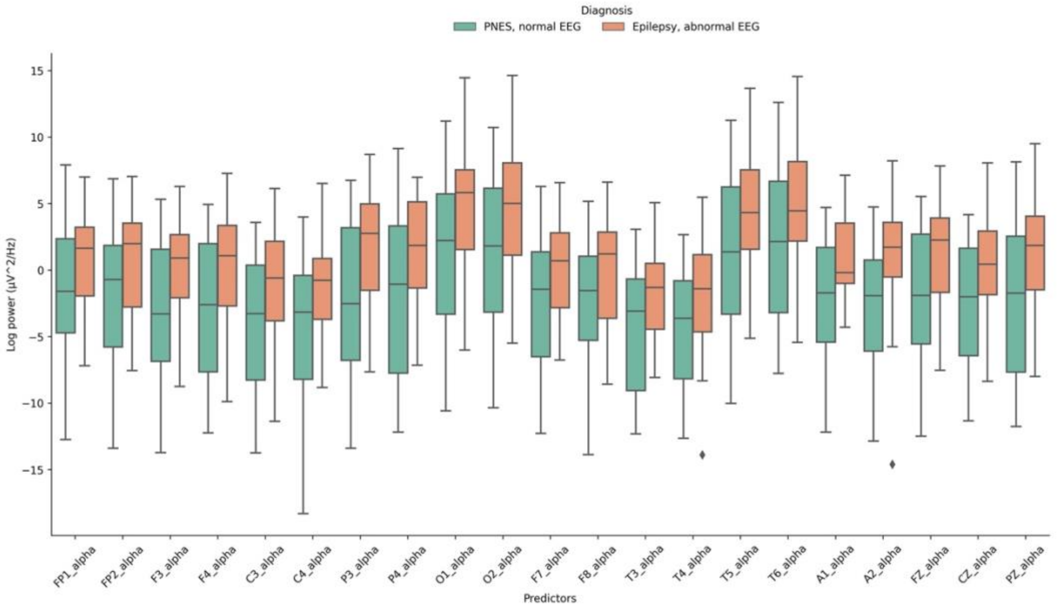

Supplementary Figure 10. Group differences in Beta power between people with PNES with normal EEG (n=57) and people with epilepsy with detected epileptiform abnormalities (n=28). See Supplementary Table 11 for corresponding numerical values.

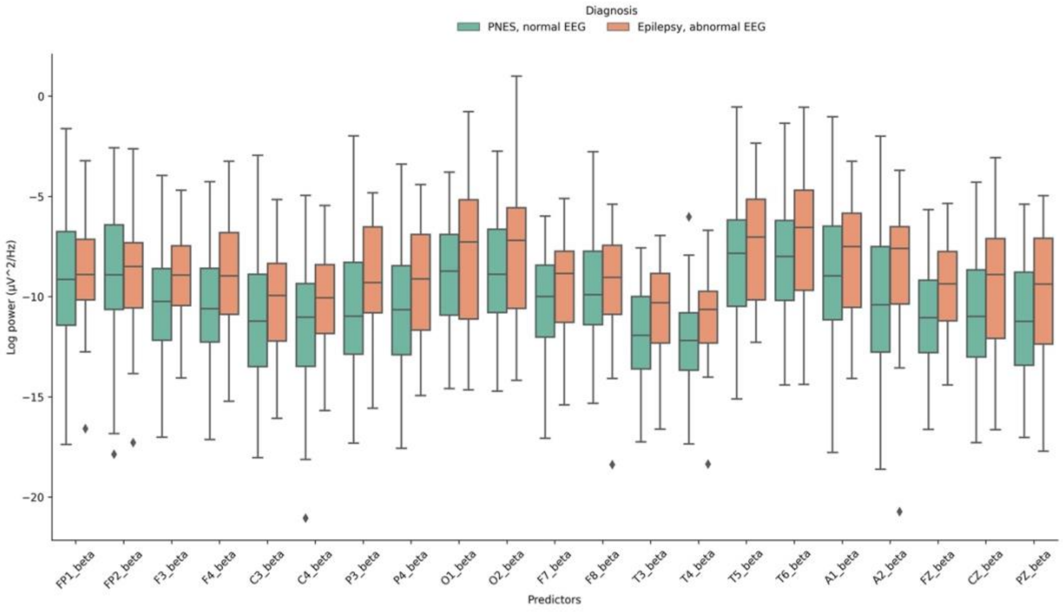

Section 3: Supplementary Material for Study 2

EEG features extraction (hctsa)

The highly comparative time series analysis (hctsa) software tool[4,5] allows computation of a high number of features (n= 7729) from time-series data. The hctsa tool includes a very large interdisciplinary library of features, such as measures summarising properties of the data distribution (e.g., mean, mode, spread, skewness), correlation through time (e.g., linear and nonlinear autocorrelation, time reversal statistics), and different measures of stationarity. It includes measures derived from information theory (e.g., Approximate Entropy, Sample Entropy, Multiscale Entropy), chaos theory (e.g., Lyapunov exponent, correlation dimension), fractal theory (e.g., fractal dimension, fluctuation analysis indices), as well as Fourier- and wavelet-transform based measures (e.g., power spectrum, wavelet decomposition).

Implementing the hctsa tool, the full set of 7729 features was extracted for each patient, and separately for each of the 21 EEG channels (feature set hash: 'dc47057e56d91670d86c8a656e05596353d291b8'). Quality of the computation was inspected and any features that had less than 100% valid values were removed. These included features that are not appropriate to fit the data, such as are those attempting to fit a Chi Square or a Rayleigh distribution to a time series including negative values, or features that require longer data segments to execute correctly. These also included a few nonlinear time series analysis features from the Tisean package [6] due to system incompatibility with part of this software (hctsa feature codes that were always excluded: NL_TISEAN_c1, NL_TISEAN_d2_1 and NL_TISEAN_d2_ac). Features with near-constant outputs and features with zero variance within each diagnostic group (epilepsy and PNES) were also removed, as these are not useful for classification problems. Features that are dependent on the exact length of the time series were also excluded, as these are non-informative of dynamical properties of interest and would have poor generalisability and applicability.

There were slight differences in the excluded features between the 21 EEG datasets. Therefore, any features that produced non-valid values in any of the datasets were removed from all 21 datasets to enable combination of results (n= 1304 features excluded). A total of 6425 valid features that were common to all datasets were further considered for the classification problem.

As part of our control analyses, analyses were repeated for two different 20s EEG segments selected at random per each participant (random segment 2 and 3). For random segment 2, a total of 6435 valid features that were common to all 21 datasets were considered for the classification problem. For random segment 3, a total of 6448 valid features were considered which were common to all 21 datasets.

Model fitting methods: technical details

We evaluated whether the 6425 features extracted could predict diagnostic class *y* (epilepsy / PNES). The same analysis pipeline as the one described for Study 1 was followed, with the addition of a feature selection step on the training set following the patient-level stratified 80:20 dataset split (Supplementary Fig. 11). In datasets including one to two hundred observations, filter-based feature selection methods are recommended.[7] The minimum Redundancy - Maximum Relevance algorithm (mRMR)[8] was used for feature selection with the mrmr_classif implementation in Python v3.10.4 to identify a subset of features that, collectively, are most informative of the diagnostic class y and the least redundant with each other. Starting from the first feature in the rank, as many features as needed to explain 75% of the cumulative variance in *y* were selected (18 features retained on average). R squared (R^2) was used as an index of explained variance.[9] Within the training set, the reduced feature set was normalised to zero mean and unit variance. The same subset of features was then retained in the test set and normalised using the same parameters as the training set. The rest of the analysis pipeline is identical to Study 1. For Study 2, the whole procedure was repeated independently for each of the 21 EEG channels. Features that were selected by multiple training folds within each channel and features that were repeatedly selected across folds and across channels are reported (Supplementary Fig. 11).

Supplementary Figure 11. Machine Learning pipeline implemented. For each of 21 EEG channels, data are split in 80:20 training-test proportion. Training data are used for feature (f) selection, normalisation parameters, and for identifying the best Radial Basis Function kernel hyperparameters through 5-fold cross validation (x 5 cv folds). The final model with the reduced features is evaluated on the test set. The procedure is repeated five times for non-overlapping test sets (x 5 outer folds) and mean performance indices are calculated. Features that are common across folds and across channels are identified.

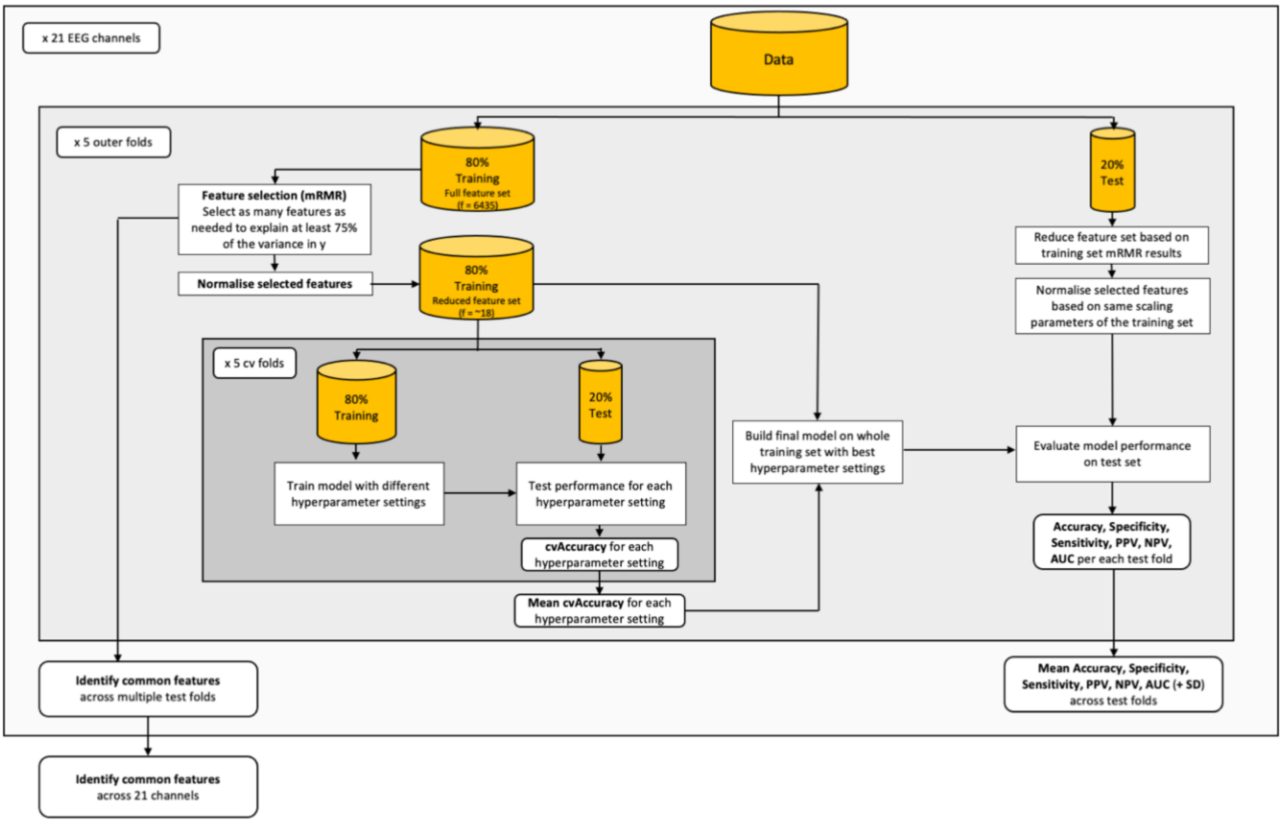

Post-hoc analyses methods

A series of post-hoc analyses were conducted to aid interpretation of the results.

I. Toy dataset code validation

In order to rule out human error, the analysis code was validated with a "toy dataset". This is a dataset artificially created to test the reliability of a classifier in predicting different classes based on informative features. The toy dataset was created to include 150 subjects from two balanced classes, a number of features equal to 100, 1000 or 6500 and a number of informative features equal to 10, 30 or 60. Performance was measured for each combination. For the most complex toy dataset (150 subjects, 6500 features, 60 of which informative), we also explored the effect of increasing the sample size to n=500.

II. Testing the appropriateness of the feature selection method

In order to ensure that the feature selection method implemented was appropriate for the problem at hand, analyses were repeated using two different methods: Mutual Information (MI)[10] and Recursive Feature Elimination (RFE).[11]

Similarly to mRMR, Mutual Information is a filter-based feature selection method whereby features are filtered based on a specified strategy. Whilst in mRMR features are filtered based on their F-statistic relationship with the outcome variable, and their correlational relationship with other features, MI filters features based on entropy. Specifically, it measures the amount of knowledge on the outcome variable (y) provided by each feature (X) and vice versa and quantifies the reduction of the uncertainty of y when X is known; it is able to measure nonlinear relationships between X and y.[10]

On the other hand, Recursive Feature Elimination is a wrapper-based method, which adopts a search (rather than a filtering) strategy for feature selection. Logistic regression coefficients are estimated for the initial feature set, and the least important features are eliminated. This is iteratively repeated on smaller and smaller feature sets, until the desired number of features is identified.[11]

MI and RFE were first tested on the toy dataset. The best performing of the two methods (RFE) was then also tested on the study dataset, and performance was measured.

III. Testing the appropriateness of the number of features selected

The number of features to be included in the model following mRMR selection was reduced to 5, as opposed to selecting as many features as needed to explain 75% of the variance. Performance was measured. This was done to ensure that the model was not confounded by an overcomplicated feature set due to the inclusion of too many "noise" features unrelated to the outcome variable. "Noise" features might affect the learning process leading to the creation of inaccurate models and to overfitting to the training set. This can happen if the model is created based on the noise in the data (e.g., due to the presence of spurious relationships) rather than relevant underlying patterns.

IV. Inspecting feature variance and feature number at increased training set size

The feature variance and the number of features selected was inspected across splits with different proportions to assess the effect of increased training set size on feature selection. If mRMR was selecting noise features unrelated to the outcome variable, a high degree of variation between the features selected would be expected across different training set sizes. This is because if features were selected only because they happened to be related to the outcome variable by chance, different data subsets would, by chance, be related to different

sets of noise features. If instead, consistency was noticed in the features selected across different subsets, this would indicate a higher chance that these are actually related to the outcome variable.

Additionally, if mRMR was selecting noise features unrelated to the outcome variable, it would be expected that the number of features needed to reach the 75% variance threshold would greatly increase at increased training set size. This is because as sample size increases, noise features that happen to be related to the outcome variable by chance would explain less and less variance in y, therefore requiring to recruit more features to reach the 75% threshold.[12]

V. Assessing intra-patient feature stability

Intra-patient feature stability was inspected across different 20 second EEG segments. This was to ensure that the features selected by mRMR are not related to transient artifacts in the data but are instead stable over the course of the same EEG recording session. To this end, mRMR was run on the whole patient cohort in one random segment; the features identified as informative were then used to predict diagnosis in two different random segments. Performance was measured. This process whereby one segment was used for feature selection and the other two for measuring performance was repeated six times (one for each combination of three random segments), and individually for each of the 21 channels. Features selected by each of the random segments were qualitatively compared, and features selected by more than one segments were reported.

VI. Ruling out overfitting

Control analyses were run to rule out the possibility that poor test set performance was due to overfitting to training data. It is expected that increased model complexity translates to better performance (lower bias) as the model is able to explain more complex relationships in the data and can have more variance in its predictions. However, as complexity increases, the model is more likely to suffer from excessive variance and overfit to the training data.

If the SVM model was overfitting to the training data, it would be expected that a simpler model with fewer parameters would improve performance, as a greater simplicity would lead to better generalisation to unseen data. In order to test whether the model was overfitting, performance was therefore compared to that of a simpler classifier with fewer parameters, namely Linear Discriminant Analysis (LDA).[13] LDA is a linear classification machine learning algorithm. It assumes that features are normally distributed and have equal variance; in this

study data, this is achieved through prior normalisation. Contrary to SVM, it focuses on group-based statistics considering the whole sample, rather than finding the boundaries for separation based on a few observations (support vectors). Its main advantages are its simplicity and reducing overfitting.

VII. Testing the appropriateness of the classifier to represent the feature space

To test the appropriateness of the classifier (SVM) to represent the feature space at hand, the analyses were repeated also implementing a different classifier. Both Linear Discriminant Analysis and SVM are projection methods, which project a high dimensional space to make the observations linearly separable. Different supervised machine learning approaches exist to represent the feature space. Here, Random Forest was implemented as an alternative method.[14] This was chosen as it has high prediction accuracy for many different types of data, is nonparametric, efficient and easily interpretable; it has been widely implemented in biological research.[15]

VIII. Low dimensional visualisation

In order to visualise whether relevant patterns exist in the data, a low dimensional visualisation technique was implemented. T-Distributed Stochastic Neighbour Embedding (t-SNE) is a dimensionality reduction technique that convert high-dimensional datasets to low-dimensional ones (2D or 3D) to allow visualisation in a scatterplot.[16] t-SNE is a probabilistic method that aims to represent the data using fewer dimensions whilst preserving as much of the high-dimensional data structure as possible. It works by converting the high-dimensional geometrical distance between observations into conditional probabilities and creating a probability distribution of neighbours (similar points) around each observation. It seeks to minimise the differences between the probability distribution built in the high- and in the low-dimensional spaces; the original high-dimensional similarities between observations are therefore reproduced in the low-dimensional space. t-SNE allows visualisation of local similarities (neighbours) and clusters as it maximises the distance between different clusters and minimises the distance between points within a single cluster.

Support Vector Machine results

Supplementary Table 12. Most commonly selected features in random segment 1. Reported are the number of times each feature was selected by mRMR across channels and across folds (t), the feature names

(bold), their description (in parenthesis), and a heat map depicting locations where features were more strongly selected, and in how many folds (0-5) these were selected within each channel.

t = 24	t = 10	t = 9	t = 8
**HT_HypothesisTest_ztest**	**SB_MotifTwo_median.u**	**IN_AutoMutualInfoStats_diff_20_gaussian.pcrossq10**	**FC_LoopLocalSimple_mean.sws_fexp_adjr2**
(Statistical hypothesis test (z-test) applied to a time series)	(Local motifs in a binary symbolization of the time series)	(Statistics on automutual information function of a time seiries)	(How simple local forecasting depends on window length)

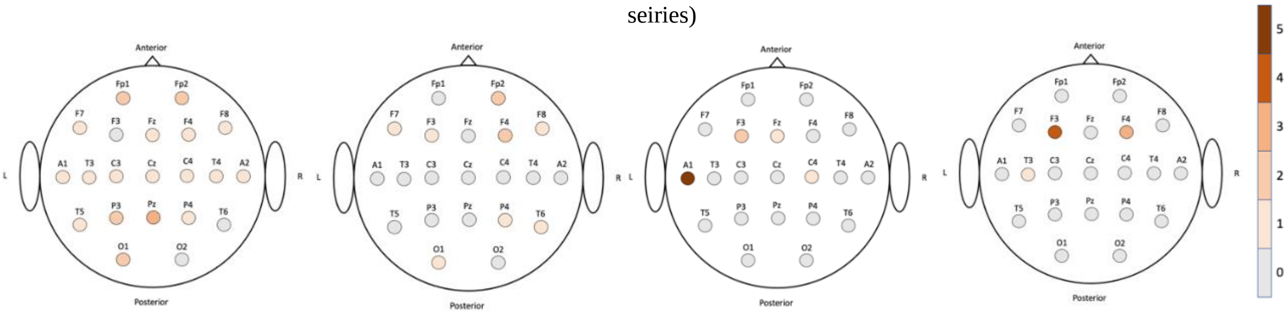

t = 7	t = 6	t = 6	t = 6
**FC_Surprise_T2_50_tau_m2quad_500.max**	**EN_Randomize_dyndist.ac4hp**	**MF_StateSpace_n4sid_3_05_1.k_3**	**NL_TSTL_ReturnTime_005_1_005_n1_1_3.pzeroscgdist**
(How surprised you would be of the next data point given recent memory)	(How time-series properties change with increasing randomization)	(State space time-series model fitting)	(Time taken for the time series to return to a similar location in phase space for a given reference point)

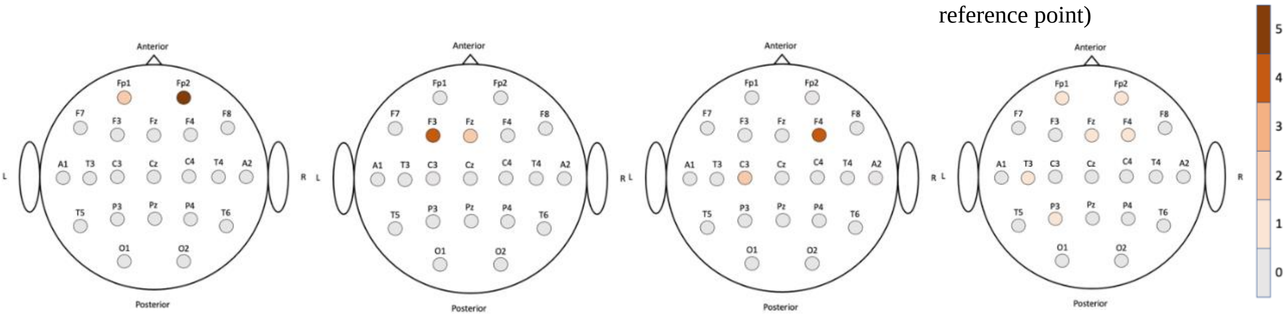

t = 6	t = 6	t = 6	t = 6
**FC_Surprise_dist_50_3_udq_500.mean**	**SD_TSTL_surrogates_1_100_3_trev.meansurr**	**SB_TransitionMatrix_21.stddiag**	**PP_Compare_medianf10.swss2_2**
(How surprised you would be of the next data point given recent memory)	(Surrogate time-series analysis)	(Transition probabilities between time-series states)	(Compare how time-series properties change after pre-processing)

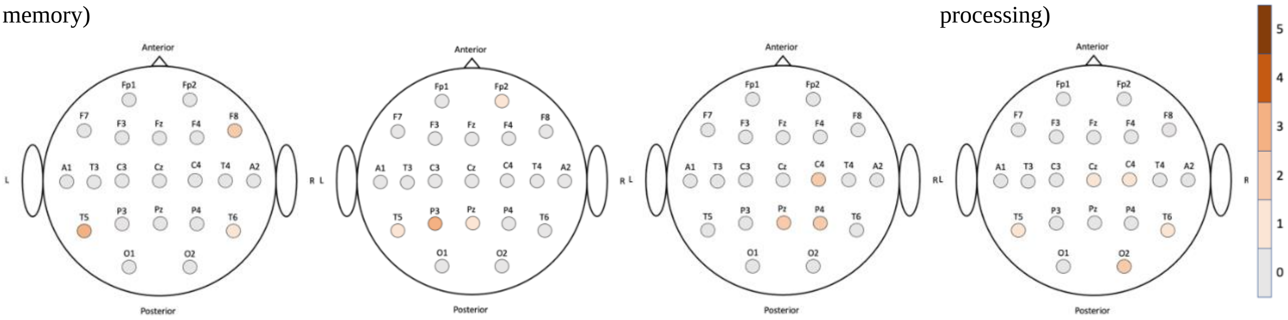

Control and post-hoc analyses results

Control analyses on different split proportions and different random segments:

Supplementary Table 13. Control analysis with 70:30 split proportion. Reported is the classification performance for selected features in 21 channels for the first randomly sampled EEG segment. Means and SD are taken across 5 cross validation folds.

Channel	Accuracy mean (SD)	Sensitivity mean (SD)	Specificity mean (SD)	PPV mean (SD)	NPV mean (SD)	AUC mean (SD)
FP1	0.53 (0.07)	0.61 (0.08)	0.44 (0.13)	0.53 (0.05)	0.52 (0.11)	0.53 (0.07)
FP2	0.48 (0.07)	0.54 (0.11)	0.41 (0.09)	0.48 (0.06)	0.47 (0.09)	0.47 (0.08)
F3	0.6 (0.06)	0.68 (0.08)	0.52 (0.15)	0.6 (0.08)	0.61 (0.05)	0.6 (0.06)
F4	0.5 (0.05)	0.51 (0.05)	0.5 (0.08)	0.51 (0.04)	0.49 (0.07)	0.5 (0.05)
C3	0.57 (0.04)	0.63 (0.06)	0.51 (0.11)	0.57 (0.04)	0.57 (0.04)	0.57 (0.04)
C4	0.52 (0.05)	0.6 (0.08)	0.44 (0.12)	0.53 (0.05)	0.52 (0.07)	0.52 (0.05)
P3	0.56 (0.07)	0.64 (0.13)	0.48 (0.15)	0.57 (0.06)	0.57 (0.1)	0.56 (0.07)
P4	0.52 (0.04)	0.55 (0.08)	0.5 (0.08)	0.53 (0.04)	0.52 (0.04)	0.53 (0.03)
O1	0.52 (0.07)	0.54 (0.07)	0.49 (0.08)	0.52 (0.06)	0.51 (0.09)	0.52 (0.07)
O2	0.52 (0.07)	0.56 (0.09)	0.48 (0.1)	0.53 (0.06)	0.51 (0.08)	0.52 (0.07)
F7	0.53 (0.05)	0.52 (0.05)	0.54 (0.08)	0.54 (0.07)	0.52 (0.05)	0.53 (0.05)
F8	0.54 (0.11)	0.58 (0.1)	0.49 (0.15)	0.55 (0.11)	0.53 (0.12)	0.54 (0.11)
T3	0.48 (0.04)	0.55 (0.08)	0.42 (0.11)	0.49 (0.03)	0.47 (0.05)	0.48 (0.04)
T4	0.48 (0.04)	0.54 (0.11)	0.43 (0.09)	0.49 (0.02)	0.48 (0.06)	0.48 (0.04)
T5	0.52 (0.06)	0.56 (0.13)	0.48 (0.06)	0.52 (0.05)	0.53 (0.08)	0.52 (0.06)
T6	0.49 (0.08)	0.52 (0.12)	0.47 (0.09)	0.5 (0.08)	0.49 (0.09)	0.49 (0.08)
A1	0.61 (0.03)	0.63 (0.14)	0.59 (0.1)	0.61 (0.01)	0.62 (0.08)	0.61 (0.03)
A2	0.5 (0.09)	0.53 (0.1)	0.47 (0.09)	0.51 (0.07)	0.5 (0.11)	0.5 (0.09)
FZ	0.53 (0.08)	0.55 (0.13)	0.5 (0.13)	0.54 (0.08)	0.52 (0.1)	0.53 (0.08)
CZ	0.46 (0.04)	0.53 (0.06)	0.39 (0.05)	0.47 (0.03)	0.45 (0.05)	0.46 (0.04)
PZ	0.49 (0.06)	0.53 (0.09)	0.46 (0.13)	0.5 (0.07)	0.48 (0.05)	0.49 (0.06)

Supplementary Table 14. Control analysis with 90:10 split proportion. Reported is the classification performance for selected features in 21 channels for the first randomly sampled EEG segment. Means and SD are taken across 5 cross validation folds.

Channel	Accuracy mean (SD)	Sensitivity mean (SD)	Specificity mean (SD)	PPV mean (SD)	NPV mean (SD)	AUC mean (SD)
FP1	0.49 (0.09)	0.52 (0.21)	0.47 (0.21)	0.5 (0.08)	0.49 (0.13)	0.49 (0.09)
FP2	0.44 (0.15)	0.45 (0.21)	0.44 (0.18)	0.44 (0.19)	0.44 (0.13)	0.44 (0.15)
F3	0.63 (0.17)	0.74 (0.21)	0.52 (0.25)	0.62 (0.17)	0.63 (0.29)	0.63 (0.17)
F4	0.6 (0.11)	0.62 (0.15)	0.58 (0.18)	0.61 (0.12)	0.6 (0.12)	0.6 (0.11)
C3	0.53 (0.09)	0.54 (0.16)	0.52 (0.12)	0.53 (0.1)	0.54 (0.12)	0.53 (0.09)
C4	0.47 (0.13)	0.54 (0.17)	0.4 (0.16)	0.48 (0.12)	0.46 (0.14)	0.47 (0.13)
P3	0.54 (0.07)	0.67 (0.15)	0.41 (0.17)	0.54 (0.07)	0.58 (0.16)	0.54 (0.07)
P4	0.52 (0.11)	0.56 (0.24)	0.48 (0.1)	0.5 (0.12)	0.56 (0.18)	0.52 (0.11)
O1	0.48 (0.12)	0.6 (0.18)	0.36 (0.16)	0.49 (0.11)	0.48 (0.18)	0.48 (0.13)
O2	0.54 (0.14)	0.61 (0.14)	0.48 (0.23)	0.57 (0.15)	0.53 (0.15)	0.55 (0.14)
F7	0.6 (0.12)	0.6 (0.2)	0.61 (0.15)	0.6 (0.14)	0.61 (0.12)	0.61 (0.11)
F8	0.58 (0.14)	0.64 (0.16)	0.52 (0.19)	0.58 (0.15)	0.57 (0.16)	0.58 (0.14)
T3	0.54 (0.15)	0.6 (0.24)	0.48 (0.22)	0.55 (0.12)	0.56 (0.24)	0.54 (0.15)
T4	0.55 (0.08)	0.57 (0.17)	0.54 (0.12)	0.55 (0.08)	0.56 (0.11)	0.56 (0.08)
T5	0.5 (0.16)	0.51 (0.26)	0.48 (0.19)	0.48 (0.22)	0.51 (0.2)	0.5 (0.17)
T6	0.48 (0.15)	0.59 (0.27)	0.38 (0.24)	0.47 (0.2)	0.42 (0.22)	0.49 (0.15)
A1	0.62 (0.07)	0.56 (0.17)	0.69 (0.14)	0.66 (0.14)	0.62 (0.08)	0.62 (0.07)
A2	0.49 (0.1)	0.55 (0.15)	0.43 (0.23)	0.51 (0.13)	0.46 (0.1)	0.49 (0.1)
FZ	0.47 (0.07)	0.55 (0.15)	0.4 (0.18)	0.49 (0.07)	0.45 (0.1)	0.48 (0.07)
CZ	0.55 (0.1)	0.55 (0.18)	0.57 (0.23)	0.58 (0.13)	0.55 (0.08)	0.56 (0.1)
PZ	0.47 (0.11)	0.5 (0.21)	0.46 (0.2)	0.48 (0.1)	0.47 (0.15)	0.48 (0.11)

Supplementary Table 15. Control analyses on random segment 2. Classification performance for selected features in 21 channels. Reported are mean (SD) across 5 cross validation folds (80:20 proportion for training and test sets).

Channel	Accuracy mean (SD)	Sensitivity mean (SD)	Specificity mean (SD)	PPV mean (SD)	NPV mean (SD)	AUC mean (SD)
FP1	0.55 (0.04)	0.53 (0.07)	0.58 (0.09)	0.57 (0.04)	0.54 (0.04)	0.55 (0.04)
FP2	0.57 (0.06)	0.6 (0.14)	0.53 (0.06)	0.56 (0.06)	0.57 (0.07)	0.57 (0.06)
F3	0.53 (0.09)	0.55 (0.08)	0.52 (0.1)	0.54 (0.08)	0.53 (0.09)	0.53 (0.09)
F4	0.43 (0.07)	0.53 (0.12)	0.32 (0.13)	0.44 (0.06)	0.38 (0.13)	0.43 (0.07)
C3	0.57 (0.04)	0.55 (0.05)	0.59 (0.12)	0.59 (0.07)	0.56 (0.03)	0.57 (0.04)

C4	0.55 (0.08)	0.55 (0.15)	0.55 (0.09)	0.55 (0.07)	0.55 (0.08)	0.55 (0.08)
P3	0.57 (0.08)	0.59 (0.17)	0.55 (0.15)	0.57 (0.08)	0.56 (0.09)	0.57 (0.08)
P4	0.61 (0.11)	0.68 (0.08)	0.53 (0.17)	0.61 (0.11)	0.6 (0.13)	0.6 (0.11)
O1	0.57 (0.09)	0.59 (0.11)	0.56 (0.11)	0.58 (0.09)	0.57 (0.09)	0.57 (0.09)
O2	0.52 (0.07)	0.6 (0.16)	0.44 (0.09)	0.52 (0.06)	0.54 (0.1)	0.52 (0.06)
F7	0.49 (0.07)	0.49 (0.07)	0.49 (0.11)	0.5 (0.07)	0.48 (0.07)	0.49 (0.07)
F8	0.56 (0.09)	0.59 (0.07)	0.52 (0.14)	0.57 (0.1)	0.54 (0.09)	0.56 (0.09)
T3	0.51 (0.12)	0.52 (0.15)	0.49 (0.19)	0.52 (0.13)	0.49 (0.13)	0.51 (0.12)
T4	0.55 (0.1)	0.57 (0.13)	0.53 (0.08)	0.55 (0.1)	0.55 (0.1)	0.55 (0.1)
T5	0.52 (0.07)	0.53 (0.11)	0.51 (0.09)	0.52 (0.07)	0.52 (0.08)	0.52 (0.07)
T6	0.55 (0.03)	0.63 (0.08)	0.48 (0.11)	0.55 (0.02)	0.55 (0.05)	0.55 (0.03)
A1	0.56 (0.05)	0.61 (0.11)	0.51 (0.07)	0.56 (0.05)	0.57 (0.07)	0.56 (0.05)
A2	0.48 (0.04)	0.51 (0.07)	0.45 (0.07)	0.49 (0.04)	0.47 (0.04)	0.48 (0.04)
FZ	0.52 (0.05)	0.49 (0.09)	0.54 (0.17)	0.54 (0.07)	0.5 (0.05)	0.52 (0.05)
CZ	0.59 (0.08)	0.57 (0.07)	0.61 (0.11)	0.61 (0.08)	0.58 (0.08)	0.59 (0.08)
PZ	0.49 (0.07)	0.51 (0.07)	0.47 (0.14)	0.5 (0.06)	0.47 (0.08)	0.49 (0.07)

Supplementary Table 16. Control analyses on random segment 3. Classification performance for selected features in 21 channels. Reported are mean (SD) across 5 cross validation folds (80:20 proportion for training and test sets).

Channel	Accuracy mean (SD)	Sensitivity mean (SD)	Specificity mean (SD)	PPV mean (SD)	NPV mean (SD)	AUC mean (SD)
FP1	0.42 (0.05)	0.43 (0.03)	0.41 (0.08)	0.43 (0.05)	0.41 (0.05)	0.42 (0.05)
FP2	0.56 (0.06)	0.61 (0.08)	0.51 (0.16)	0.57 (0.07)	0.55 (0.07)	0.56 (0.06)
F3	0.51 (0.09)	0.52 (0.13)	0.5 (0.11)	0.51 (0.09)	0.5 (0.1)	0.51 (0.09)
F4	0.53 (0.08)	0.57 (0.14)	0.49 (0.04)	0.53 (0.06)	0.54 (0.1)	0.53 (0.08)
C3	0.53 (0.07)	0.53 (0.16)	0.52 (0.15)	0.53 (0.06)	0.52 (0.08)	0.53 (0.07)
C4	0.51 (0.04)	0.55 (0.08)	0.48 (0.09)	0.52 (0.04)	0.51 (0.05)	0.51 (0.04)
P3	0.49 (0.07)	0.55 (0.1)	0.44 (0.11)	0.5 (0.07)	0.49 (0.08)	0.49 (0.07)
P4	0.49 (0.07)	0.52 (0.14)	0.46 (0.07)	0.49 (0.06)	0.49 (0.08)	0.49 (0.07)
O1	0.52 (0.08)	0.55 (0.11)	0.49 (0.1)	0.52 (0.07)	0.52 (0.09)	0.52 (0.08)
O2	0.55 (0.08)	0.53 (0.07)	0.56 (0.14)	0.57 (0.1)	0.53 (0.07)	0.55 (0.08)
F7	0.53 (0.06)	0.53 (0.12)	0.53 (0.14)	0.54 (0.07)	0.52 (0.05)	0.53 (0.06)
F8	0.46 (0.1)	0.48 (0.11)	0.44 (0.13)	0.47 (0.09)	0.45 (0.11)	0.46 (0.1)
T3	0.44 (0.15)	0.48 (0.11)	0.41 (0.23)	0.47 (0.14)	0.4 (0.19)	0.44 (0.15)
T4	0.58 (0.04)	0.61 (0.14)	0.55 (0.07)	0.58 (0.03)	0.59 (0.06)	0.58 (0.04)
T5	0.49 (0.07)	0.48 (0.11)	0.51 (0.17)	0.51 (0.08)	0.48 (0.06)	0.49 (0.07)
T6	0.52 (0.1)	0.59 (0.18)	0.45 (0.11)	0.52 (0.09)	0.53 (0.14)	0.52 (0.1)
A1	0.59 (0.07)	0.6 (0.09)	0.59 (0.14)	0.61 (0.1)	0.59 (0.06)	0.59 (0.07)
A2	0.5 (0.02)	0.56 (0.07)	0.44 (0.05)	0.51 (0.02)	0.49 (0.01)	0.5 (0.02)
FZ	0.49 (0.08)	0.51 (0.16)	0.48 (0.09)	0.49 (0.09)	0.49 (0.09)	0.49 (0.08)
CZ	0.52 (0.1)	0.52 (0.13)	0.52 (0.08)	0.52 (0.1)	0.52 (0.11)	0.52 (0.1)
PZ	0.55 (0.06)	0.63 (0.05)	0.48 (0.08)	0.55 (0.05)	0.55 (0.08)	0.55 (0.06)

Supplementary Table 17. Control analyses on random segment 2; most commonly selected features across different channels and different folds.

Feature	Times selected
SY_LocalGlobal_l10.median	11
EN_Randomize_permute.xc1diff	9
PP_Compare_spline44.swms5_2	8

DN_OutlierInclude_n_001.stdrflrmse	8
EN_mse_1-10_2_015_diff1.sampen_s4	8
SB_TransitionpAlphabet_20_ac.meandiagfexp_r2	7
EN_Randomize_statdist.swss5_1fexpb	7
SB_BinaryStats_iqr.meanstretchdiff	7
PH_ForcePotential_dblwell_2_005_02.ac10	7
FC_LoopLocalSimple_mean.swm_stdn	6
NW_VisibilityGraph_horiz.dpowerk_r2	6
PP_Compare_resample_2_1.swms5_1	6
NL_MS_nlpe_3_ac.ac3n	6
PH_ForcePotential_dblwell_2_005_02.ac50	6
EN_Randomize_dyndist_swss5_1fexprmse	6
SY_SlidingWindow_mom4_ent2_10	6
SY_LocalGlobal_unicg20.kurtosis	6

Supplementary Table 18. Control analyses on random segment 3; most commonly selected features across different channels and different folds.

Feature	Times selected
HT_HypothesisTest_ztest	19
PH_ForcePotential_sine_10_004_10.proppos	10
EN_Randomize_permute.ac1fexprmse	9
SB_BinaryStats_iqr.meanstretchdiff	9
FC_Surprise_T2_100_4_udq_500.tstat	8
SB_BinaryStats_diff.pupstat2	8
FC_Surprise_T2_100_5_udq_500.min	7
WL_scal2frq_db3_max_1.lmax	6
FC_Surprise_T2_100_4_udq_500.median	6
SY_VarRatioTest_24682468_00001111.periodminpValue	6
EN_Randomize_permute.ac1fexpr2	6
MF_armax_1_1_05_1.ac3n	6
EN_Randomize_statdist.ac2diff	6
MF_GP_LocalPrediction_covSEiso_covNoise_10_3_20_randomgap.minstderr_run	6
SY_SpreadRandomLocal_100_100.stdskew	6

PH_ForcePotential_sine_1_1_1.tau	6

Post-hoc analyses:

I. Toy dataset code validation

The code was validated on a toy dataset to rule out human error. Results confirmed that the pipeline implemented was valid as it was possible to identify informative features and reach accurate prediction of two classes in the toy dataset. This analysis also demonstrated that the ability of feature selection methods to identify informative features diminished as a function of dataset complexity (as represented by sample size, number of features and number of informative features present in the dataset). In a toy dataset including 150 subjects from two balanced classes, performance using mRMR deteriorated as the number of features increased (1000 features, 10 informative: 85% accuracy; 6500 features, 10 informative: 77% accuracy) and as the number of informative features increased (6500 features, 30 informative: 64% accuracy; 6500 features, 60 informative: 49% accuracy). If in the context of a complex dataset (6500 features, 60 of which are informative), the sample size was increased (n=500 instead of n=150), performance improved considerably (74% accuracy). Detailed results for this analysis are presented in Supplementary Table 19.

Supplementary Table 19. Post-hoc analyses on toy dataset to ensure the correctness of the code and to explore the classification performance of different feature selection methods with increasing dataset complexity. Within each cell, the tables display how many times each feature was selected across the 5 outer folds. mRMR = maximum Relevance Minimum Redundancy; MI = Mutual Information; RFE = Recursive Feature Elimination.

**Nr subjects / Nr features**	**Informative**	**mRMR**<br>Classification accuracy (total nr of features selected across 5 folds)	**MI**<br>Classification accuracy (total nr of features selected across 5 folds)	**RFE**<br>Classification accuracy (total nr of features selected across 5 folds)
150 / 100	First 10	**82%** (22)<br>FeatureName TimesSelected<br>0 2.0 5<br>1 6.0 5<br>2 4.0 3<br>3 5.0 3<br>4 29.0 1	**84%** (17)<br>FeatureName TimesSelected<br>0 2.0 5<br>1 6.0 5<br>2 5.0 4<br>3 1<br>4 68.0 1	**78%** (18)<br>FeatureName TimesSelected<br>0 2.0 5<br>1 3.0 5<br>2 4.0 5<br>3 5.0 3<br>4 1

<table>
<tr><td>150 /1000</td><td>First 10</td><td>85% (28)<table><tr><th></th><th>FeatureName</th><th>TimesSelected</th></tr><tr><td>0</td><td>2.0</td><td>5</td></tr><tr><td>1</td><td>6.0</td><td>5</td></tr><tr><td>2</td><td>5.0</td><td>4</td></tr><tr><td>3</td><td>154.0</td><td>2</td></tr><tr><td>4</td><td>627.0</td><td>2</td></tr><tr><td>5</td><td>751.0</td><td>2</td></tr><tr><td>6</td><td>730.0</td><td>1</td></tr></table></td><td>82% (38)<table><tr><th></th><th>FeatureName</th><th>TimesSelected</th></tr><tr><td>0</td><td>2.0</td><td>5</td></tr><tr><td>1</td><td>6.0</td><td>5</td></tr><tr><td>2</td><td>5.0</td><td>4</td></tr><tr><td>3</td><td>832.0</td><td>2</td></tr><tr><td>4</td><td>7.0</td><td>1</td></tr><tr><td>5</td><td>445.0</td><td>1</td></tr><tr><td>6</td><td>366.0</td><td>1</td></tr></table></td><td>87% (20)<table><tr><th></th><th>FeatureName</th><th>TimesSelected</th></tr><tr><td>0</td><td>2</td><td>5</td></tr><tr><td>1</td><td>5</td><td>5</td></tr><tr><td>2</td><td>4</td><td>4</td></tr><tr><td>3</td><td>6</td><td>4</td></tr><tr><td>4</td><td>3</td><td>2</td></tr></table></td></tr>
<tr><td>150 / 6500</td><td>First 10</td><td>77% (31)<table><tr><th></th><th>FeatureName</th><th>TimesSelected</th></tr><tr><td>0</td><td>2.0</td><td>5</td></tr><tr><td>1</td><td>6.0</td><td>4</td></tr><tr><td>2</td><td>5.0</td><td>3</td></tr><tr><td>3</td><td>1883.0</td><td>2</td></tr><tr><td>4</td><td>5821.0</td><td>1</td></tr></table></td><td>74% (74)<table><tr><th></th><th>FeatureName</th><th>TimesSelected</th></tr><tr><td>0</td><td>2.0</td><td>5</td></tr><tr><td>1</td><td>6.0</td><td>4</td></tr><tr><td>2</td><td>3380.0</td><td>2</td></tr><tr><td>3</td><td>2883.0</td><td>2</td></tr><tr><td>4</td><td>1544.0</td><td>2</td></tr><tr><td>5</td><td>4491.0</td><td>1</td></tr></table></td><td>83% (19)<table><tr><th></th><th>FeatureName</th><th>TimesSelected</th></tr><tr><td>0</td><td>2.0</td><td>5</td></tr><tr><td>1</td><td>5.0</td><td>5</td></tr><tr><td>2</td><td>4.0</td><td>4</td></tr><tr><td>3</td><td>6.0</td><td>3</td></tr><tr><td>4</td><td>3.0</td><td>2</td></tr><tr><td>5</td><td></td><td>1</td></tr></table></td></tr>
<tr><td>150 / 6500</td><td>First 30</td><td>64% (42)<table><tr><th></th><th>FeatureName</th><th>TimesSelected</th></tr><tr><td>0</td><td>3.0</td><td>5</td></tr><tr><td>1</td><td>22.0</td><td>2</td></tr><tr><td>2</td><td>8.0</td><td>2</td></tr><tr><td>3</td><td>26.0</td><td>2</td></tr><tr><td>4</td><td>11.0</td><td>2</td></tr><tr><td>5</td><td>4005.0</td><td>1</td></tr><tr><td>6</td><td>6377.0</td><td>1</td></tr><tr><td>7</td><td>1613.0</td><td>1</td></tr><tr><td>8</td><td>1575.0</td><td>1</td></tr><tr><td>9</td><td>2961.0</td><td>1</td></tr></table></td><td>59% (235)<table><tr><th></th><th>FeatureName</th><th>TimesSelected</th></tr><tr><td>0</td><td>24.0</td><td>4</td></tr><tr><td>1</td><td>5261.0</td><td>4</td></tr><tr><td>2</td><td>1965.0</td><td>3</td></tr><tr><td>3</td><td>1400.0</td><td>3</td></tr><tr><td>4</td><td>3694.0</td><td>3</td></tr><tr><td>5</td><td>5178.0</td><td>3</td></tr><tr><td>6</td><td>1629.0</td><td>3</td></tr><tr><td>7</td><td>1199.0</td><td>3</td></tr><tr><td>8</td><td>1035.0</td><td>3</td></tr><tr><td>9</td><td>464.0</td><td>3</td></tr><tr><td>10</td><td>22.0</td><td>2</td></tr><tr><td>11</td><td>3653.0</td><td>2</td></tr></table></td><td>81% (29)<table><tr><th></th><th>FeatureName</th><th>TimesSelecte</th></tr><tr><td>0</td><td>3.0</td><td>[illegible]</td></tr><tr><td>1</td><td>1.0</td><td>[illegible]</td></tr><tr><td>2</td><td>8.0</td><td>[illegible]</td></tr><tr><td>3</td><td>19.0</td><td>[illegible]</td></tr><tr><td>4</td><td>11.0</td><td>[illegible]</td></tr><tr><td>5</td><td>22.0</td><td>[illegible]</td></tr><tr><td>6</td><td>26.0</td><td>[illegible]</td></tr><tr><td>7</td><td>6.0</td><td>[illegible]</td></tr><tr><td>8</td><td>23.0</td><td>[illegible]</td></tr><tr><td>9</td><td></td><td>[illegible]</td></tr></table></td></tr>
<tr><td>150 / 6500</td><td>First 60</td><td>49% (55)<table><tr><th></th><th>FeatureName</th><th>TimesSelected</th></tr><tr><td>0</td><td>4418.0</td><td>3</td></tr><tr><td>1</td><td>26.0</td><td>3</td></tr><tr><td>2</td><td>5.0</td><td>2</td></tr><tr><td>3</td><td>2201.0</td><td>2</td></tr><tr><td>4</td><td>1755.0</td><td>1</td></tr><tr><td>5</td><td>1681.0</td><td>1</td></tr><tr><td>6</td><td>647.0</td><td>1</td></tr></table></td><td>52% (220)<table><tr><th></th><th>FeatureName</th><th>TimesSelected</th></tr><tr><td>0</td><td>4075.0</td><td>4</td></tr><tr><td>1</td><td>1153.0</td><td>3</td></tr><tr><td>2</td><td>5635.0</td><td>3</td></tr><tr><td>3</td><td>26.0</td><td>3</td></tr><tr><td>4</td><td>6155.0</td><td>3</td></tr><tr><td>5</td><td>5426.0</td><td>3</td></tr><tr><td>6</td><td>2995.0</td><td>3</td></tr><tr><td>7</td><td>5192.0</td><td>3</td></tr><tr><td>8</td><td>1244.0</td><td>2</td></tr></table></td><td>58% (50)<table><tr><th></th><th>FeatureName</th><th>TimesSelected</th></tr><tr><td>0</td><td>26.0</td><td>4</td></tr><tr><td>1</td><td>37.0</td><td>3</td></tr><tr><td>2</td><td>56.0</td><td>2</td></tr><tr><td>3</td><td>1755.0</td><td>2</td></tr><tr><td>4</td><td>266.0</td><td>2</td></tr><tr><td>5</td><td>2067.0</td><td>2</td></tr><tr><td>6</td><td>268.0</td><td>2</td></tr><tr><td>7</td><td>670.0</td><td>1</td></tr></table></td></tr>
<tr><td>500 / 6500</td><td>First 60</td><td>74% (78)<table><tr><th></th><th>FeatureName</th><th>TimesSelected</th></tr><tr><td>0</td><td>3.0</td><td>5</td></tr><tr><td>1</td><td>42.0</td><td>5</td></tr><tr><td>2</td><td>18.0</td><td>5</td></tr><tr><td>3</td><td>38.0</td><td>4</td></tr><tr><td>4</td><td>26.0</td><td>4</td></tr><tr><td>5</td><td>20.0</td><td>4</td></tr><tr><td>6</td><td>52.0</td><td>4</td></tr><tr><td>7</td><td>22.0</td><td>3</td></tr><tr><td>8</td><td>30.0</td><td>3</td></tr><tr><td>9</td><td>7.0</td><td>3</td></tr><tr><td>10</td><td>33.0</td><td>3</td></tr><tr><td>11</td><td>9.0</td><td>2</td></tr><tr><td>12</td><td>2899.0</td><td>2</td></tr><tr><td>13</td><td>48.0</td><td>2</td></tr></table></td><td>56% (841)<table><tr><th></th><th>FeatureName</th><th>TimesSelected</th></tr><tr><td>0</td><td>22.0</td><td>5</td></tr><tr><td>1</td><td>1370.0</td><td>5</td></tr><tr><td>2</td><td>6282.0</td><td>5</td></tr><tr><td>3</td><td>4932.0</td><td>5</td></tr><tr><td>4</td><td>2748.0</td><td>5</td></tr><tr><td>5</td><td>5449.0</td><td>5</td></tr><tr><td>6</td><td>5659.0</td><td>5</td></tr><tr><td>7</td><td>4527.0</td><td>4</td></tr><tr><td>8</td><td>1525.0</td><td>4</td></tr><tr><td>9</td><td>374.0</td><td>4</td></tr><tr><td>10</td><td>1245.0</td><td>4</td></tr><tr><td>11</td><td>6468.0</td><td>4</td></tr><tr><td>12</td><td>9.0</td><td>4</td></tr></table></td><td>84% (77)<table><tr><th></th><th>FeatureName</th><th>TimesSelected</th></tr><tr><td>0</td><td>0.0</td><td>5</td></tr><tr><td>1</td><td>7.0</td><td>5</td></tr><tr><td>2</td><td>22.0</td><td>5</td></tr><tr><td>3</td><td>26.0</td><td>5</td></tr><tr><td>4</td><td>30.0</td><td>5</td></tr><tr><td>5</td><td>33.0</td><td>5</td></tr><tr><td>6</td><td>39.0</td><td>5</td></tr><tr><td>7</td><td>40.0</td><td>5</td></tr><tr><td>8</td><td>43.0</td><td>5</td></tr><tr><td>9</td><td>20.0</td><td>4</td></tr><tr><td>10</td><td>42.0</td><td>4</td></tr><tr><td>11</td><td>48.0</td><td>4</td></tr><tr><td>12</td><td>3.0</td><td>3</td></tr><tr><td>13</td><td>38.0</td><td>3</td></tr><tr><td>14</td><td>12.0</td><td>2</td></tr><tr><td>15</td><td>14.0</td><td>2</td></tr><tr><td>16</td><td>51.0</td><td>2</td></tr><tr><td>17</td><td>44.0</td><td>2</td></tr><tr><td>18</td><td>45.0</td><td>2</td></tr><tr><td>19</td><td></td><td>1</td></tr></table></td></tr>
</table>

II. Appropriateness of the feature selection method

Different feature selection methods (MI and RFE) were tested on the toy dataset (Supplementary Table 19). These all displayed similar behaviour at increased feature set size and increased informative feature set size, similarly to mRMR as described above. RFE was the most reliable at identifying informative features, although this also selected random noise features in the presence of high feature set size (n=6500), high informative feature set size (n=60), and low sample size (n=150).

SVM analyses on our EEG dataset were repeated implementing RFE. Results showed that this feature selection method did not improve overall performance (Supplementary Table 20). However, performance reached 70% classification accuracy in A1, C3 and T5 for the subgroup of patients with epilepsy-specific abnormalities captured later in the course of the EEG recordings (data not shown). Most of the features that were most commonly selected by mRMR were also commonly selected by RFE (data not shown).

Supplementary Table 20. Post-hoc analyses implementing Recursive Feature Elimination as a feature selection method (instead of mRMR) on the first random segment. Reported are results for the main machine learning pipeline (Support Vector Machine model with 80:20 split proportion).

Channel	Accuracy mean (SD)	Sensitivity mean (SD)	Specificity mean (SD)	PPV mean (SD)	NPV mean (SD)	AUC mean (SD)
FP1	0.55 (0.04)	0.47 (0.14)	0.63 (0.08)	0.56 (0.04)	0.54 (0.04)	0.55 (0.04)
FP2	0.5 (0.07)	0.52 (0.1)	0.48 (0.1)	0.51 (0.07)	0.49 (0.06)	0.5 (0.06)
F3	0.58 (0.09)	0.64 (0.16)	0.52 (0.04)	0.57 (0.09)	0.6 (0.1)	0.58 (0.09)
F4	0.55 (0.05)	0.53 (0.07)	0.58 (0.06)	0.56 (0.04)	0.55 (0.05)	0.55 (0.05)
C3	0.59 (0.07)	0.61 (0.07)	0.58 (0.12)	0.6 (0.08)	0.59 (0.08)	0.6 (0.07)
C4	0.47 (0.04)	0.48 (0.15)	0.46 (0.17)	0.48 (0.06)	0.45 (0.07)	0.47 (0.05)
P3	0.51 (0.1)	0.56 (0.13)	0.46 (0.24)	0.53 (0.12)	0.47 (0.11)	0.51 (0.1)
P4	0.47 (0.06)	0.53 (0.0)	0.4 (0.12)	0.48 (0.06)	0.44 (0.07)	0.47 (0.06)
O1	0.54 (0.09)	0.56 (0.09)	0.52 (0.2)	0.57 (0.1)	0.52 (0.09)	0.54 (0.09)
O2	0.56 (0.08)	0.6 (0.11)	0.52 (0.07)	0.56 (0.07)	0.56 (0.1)	0.56 (0.08)
F7	0.53 (0.07)	0.57 (0.11)	0.48 (0.09)	0.53 (0.07)	0.53 (0.08)	0.53 (0.07)
F8	0.46 (0.04)	0.47 (0.06)	0.45 (0.06)	0.47 (0.04)	0.45 (0.05)	0.46 (0.04)
T3	0.46 (0.07)	0.43 (0.09)	0.5 (0.07)	0.46 (0.08)	0.46 (0.06)	0.46 (0.07)
T4	0.55 (0.11)	0.57 (0.09)	0.53 (0.18)	0.57 (0.11)	0.53 (0.12)	0.55 (0.11)

T5	0.53 (0.09)	0.59 (0.05)	0.47 (0.23)	0.56 (0.13)	0.5 (0.1)	0.53 (0.1)
T6	0.44 (0.08)	0.41 (0.11)	0.47 (0.09)	0.44 (0.1)	0.44 (0.08)	0.44 (0.08)
A1	0.55 (0.03)	0.61 (0.08)	0.49 (0.07)	0.55 (0.02)	0.56 (0.04)	0.55 (0.03)
A2	0.45 (0.08)	0.48 (0.15)	0.43 (0.08)	0.45 (0.09)	0.45 (0.08)	0.45 (0.08)
FZ	0.45 (0.09)	0.41 (0.14)	0.48 (0.1)	0.45 (0.11)	0.45 (0.09)	0.45 (0.09)
CZ	0.55 (0.08)	0.55 (0.11)	0.56 (0.17)	0.57 (0.09)	0.54 (0.09)	0.55 (0.08)
PZ	0.47 (0.12)	0.53 (0.19)	0.41 (0.14)	0.47 (0.11)	0.48 (0.15)	0.47 (0.12)

III. Appropriateness of the number of features selected

When reducing the number of mRMR features to include in the model to the first 5 (as opposite to as many features as needed to explain 75% of the variance in *y*), no performance improvement was noticed; performance was comparable to or slightly worse than the original analyses (Supplementary Table 21). This indicated that the original model was not confounded by an overcomplicated feature set.

Supplementary Table 21. Post-hoc analysis with reduced feature number on the first random segment. Only the first five features as identified by mRMR were included in the main SVM model with 70:30 split proportion, and performance was measured.

Channel	Accuracy mean (SD)	Sensitivity mean (SD)	Specificity mean (SD)	PPV mean (SD)	NPV mean (SD)	AUC mean (SD)
FP1	0.49 (0.08)	0.57 (0.14)	0.41 (0.1)	0.5 (0.07)	0.49 (0.11)	0.49 (0.08)
FP2	0.49 (0.04)	0.51 (0.1)	0.48 (0.06)	0.5 (0.04)	0.49 (0.05)	0.49 (0.04)
F3	0.5 (0.07)	0.57 (0.11)	0.43 (0.15)	0.51 (0.07)	0.49 (0.09)	0.5 (0.07)
F4	0.57 (0.05)	0.68 (0.17)	0.47 (0.18)	0.57 (0.05)	0.6 (0.05)	0.58 (0.05)
C3	0.57 (0.05)	0.68 (0.12)	0.47 (0.08)	0.56 (0.04)	0.6 (0.08)	0.57 (0.05)
C4	0.51 (0.08)	0.59 (0.1)	0.42 (0.12)	0.51 (0.06)	0.5 (0.1)	0.5 (0.08)
P3	0.45 (0.12)	0.55 (0.14)	0.36 (0.17)	0.47 (0.12)	0.43 (0.13)	0.45 (0.12)
P4	0.47 (0.05)	0.55 (0.14)	0.38 (0.13)	0.47 (0.05)	0.44 (0.07)	0.46 (0.05)
O1	0.55 (0.09)	0.51 (0.15)	0.59 (0.16)	0.56 (0.11)	0.53 (0.09)	0.55 (0.1)
O2	0.54 (0.07)	0.61 (0.2)	0.47 (0.11)	0.53 (0.05)	0.58 (0.13)	0.54 (0.07)
F7	0.51 (0.09)	0.51 (0.09)	0.51 (0.11)	0.52 (0.09)	0.5 (0.09)	0.51 (0.09)
F8	0.51 (0.09)	0.52 (0.23)	0.51 (0.13)	0.49 (0.13)	0.54 (0.09)	0.51 (0.08)
T3	0.55 (0.1)	0.57 (0.2)	0.54 (0.06)	0.54 (0.11)	0.57 (0.11)	0.55 (0.1)
T4	0.54 (0.08)	0.45 (0.08)	0.63 (0.11)	0.56 (0.09)	0.52 (0.07)	0.54 (0.08)

T5	0.52 (0.09)	0.52 (0.18)	0.52 (0.08)	0.51 (0.09)	0.53 (0.11)	0.52 (0.09)
T6	0.49 (0.04)	0.55 (0.11)	0.44 (0.14)	0.5 (0.04)	0.48 (0.05)	0.49 (0.04)
A1	0.57 (0.08)	0.56 (0.07)	0.58 (0.16)	0.59 (0.1)	0.55 (0.06)	0.57 (0.08)
A2	0.51 (0.12)	0.6 (0.18)	0.42 (0.06)	0.51 (0.09)	0.53 (0.18)	0.51 (0.12)
FZ	0.47 (0.04)	0.45 (0.11)	0.49 (0.16)	0.48 (0.05)	0.46 (0.05)	0.47 (0.04)
CZ	0.47 (0.08)	0.6 (0.15)	0.34 (0.07)	0.48 (0.06)	0.47 (0.11)	0.47 (0.08)
PZ	0.47 (0.07)	0.61 (0.16)	0.33 (0.11)	0.48 (0.06)	0.46 (0.12)	0.47 (0.07)

IV. Feature variance and feature number at increased training set size

When increasing the size of the training set, the average number of features selected to reach the 75% threshold of explained variance in *y* increased only slightly (Supplementary Table 22). This indicates that the majority of the features selected by mRMR are likely related to the outcome variable, rather than being noise features. As detailed above in the subset analyses section, features selected across different split proportions were consistent, providing further evidence for the selected features to be truly related to the outcome variable rather than being related by chance.

Supplementary Table 22. Number of selected features as a function of training set size.

Training-test split proportion	Number of selected features; mean (SD) across folds and across channels
70:30	12.11 (1.4)
80:20	13.47 (1.71)
90:10	14.72 (1.8)
100:0	15.25 (1.73)

V. Assessing intra-patient feature stability

Intra-patient feature stability was assessed; informative features were identified using the whole data from one EEG segment, and diagnosis was predicted based on those features in two different EEG segments. Results at the whole group level indicated that in all channels, prediction accuracy was not much better than chance (49-57%; Supplementary Table 23); however, prediction accuracy was high (67-83%) for the subgroup of people with epileptiform abnormalities detected later in the course of the recordings. This was observed in all channels, and on average, in three out of the six predictions run for each channel.

Qualitative comparison of the features selected across different segments revealed that variability was quite high (Supplementary Table 23). Within each channel, only one to three features were selected more than once across different EEG segments (out of an average of 75 features identified in each segment across folds). Within each channel, no feature was selected by all three segments. For five of the channels, there was no feature overlap between segments. When examining whether the same features got selected across different channels, a single feature was found (HT_HypothesisTest_ztest) which was selected by two neighbouring channels, O1 and T5 in two of the segments (Supplementary Table 23).

Supplementary Table 23. Post-hoc analysis to assess intra-patient feature stability across different random segments. For each channel, reported are the features that were selected by mRMR in more than one random segment, and the classification accuracy obtained when features identified as informative in one EEG segment were used to predict diagnosis in two different EEG segments. Mean and standard deviations are taken across six predictions (where one segment is iteratively used for feature selection, and the other two for testing). In bold, the only feature that was consistently selected across different channel locations.

Channel	Feature Name<br>(Times selected across 3 EEG segments)	Accuracy mean (SD) for SVM classification based on features selected in different segment
FP1	-	0.49 (0.06)
FP2	-	0.49 (0.04)
F3	MF_StateSpace_n4sid_3_05_1.p3_5 (2)	0.56 (0.04)
F4	ST_LocalExtrema_l50.uord (2)<br>MF_ExpSmoothing_05_best.ac3n (2)	0.51 (0.07)
C3	-	0.53 (0.02)
C4	CO_Embed2_Basic_tau.stdincircle (2)<br>PP_Compare_diff1.swms5_1 (2)	0.55 (0.04)
P3	PH_ForcePotential_dblwell_1_05_02.ac10 (2)<br>PH_Walker_prop_11.sw_taudiff (2)	0.55 (0.06)
P4	EN_Randomize_permute.xc1fexpr2 (2)	0.5 (0.06)
O1	**HT_HypothesisTest_ztest** (2)	0.57 (0.06)
O2	MF_StateSpace_n4sid_2_05_1.normp (2)	0.55 (0.05)
F7	NL_crptool_fnn_10_2_1.pdrop (2)<br>EN_Randomize_statdist.swss5_1fexpb (2)	0.49 (0.02)
F8	EN_Randomize_statdist.ac1diff (2)	0.53 (0.04)
T3	EN_DistributionEntropy_raw_ks__001 (2)	0.51 (0.03)
T4	EN_Randomize_dyndist.swss5_1hp (2)<br>PH_ForcePotential_sine_1_1_1.tau (2)	0.53 (0.03)

T5	**HT_HypothesisTest_ztest** (2)<br>FC_Surprise_dist_50_3_udq_500.mean (2)<br>SY_LocalGlobal_l10.std (2)	0.55 (0.04)
T6	PP_Compare_poly2.kscn_relent (2)	0.53 (0.05)
A1	-	0.55 (0.05)
A2	NL_DVV_3_100_2_50_10_default.numZeroCrossings (2)	0.52 (0.06)
FZ	-	0.54 (0.04)
CZ	WL_dwtcoeff_db3_5.mind_l4 (2)<br>MF_arfit_1_8_sbc.hasInfper (2)<br>EX_MovingThreshold_01_01.stdkickf (2)	0.55 (0.02)
PZ	MF_GP_LocalPrediction_covSEiso_covNoise_10_3_<br>20_randomgap.maxabserr_run (2)	0.52 (0.05)

VI. Ruling out overfitting

In order to rule out overfitting, it was explored whether a simpler model (Linear Discriminant Analysis) would improve results. Results of the LDA model were largely comparable to those observed when implementing SVM (Supplementary Table 24). This suggests that the SVM model was not overfitting the training set (therefore performing poorly on novel observations), as if that was the case using a simpler model with fewer parameters such as LDA would likely lead to an improvement in the classification performance.

Supplementary Table 24. Post-hoc Linear Discriminant Analysis on the first random segment. LDA was implemented instead of SVM as a classification method. The rest of the pipeline remained unchanged.

Channel	Accuracy mean (SD)	Sensitivity mean (SD)	Specificity mean (SD)	PPV mean (SD)	NPV mean (SD)	AUC mean (SD)
FP1	0.54 (0.03)	0.55 (0.17)	0.54 (0.15)	0.55 (0.03)	0.54 (0.04)	0.54 (0.03)
FP2	0.54 (0.08)	0.55 (0.16)	0.54 (0.09)	0.54 (0.07)	0.55 (0.11)	0.54 (0.08)
F3	0.59 (0.07)	0.64 (0.12)	0.55 (0.05)	0.59 (0.05)	0.61 (0.09)	0.59 (0.07)
F4	0.57 (0.06)	0.67 (0.08)	0.47 (0.1)	0.56 (0.06)	0.58 (0.09)	0.57 (0.06)
C3	0.59 (0.04)	0.61 (0.13)	0.56 (0.17)	0.6 (0.07)	0.59 (0.04)	0.59 (0.05)
C4	0.53 (0.07)	0.56 (0.17)	0.49 (0.1)	0.53 (0.07)	0.54 (0.1)	0.53 (0.07)
P3	0.53 (0.07)	0.63 (0.12)	0.43 (0.11)	0.53 (0.07)	0.53 (0.09)	0.53 (0.07)
P4	0.5 (0.06)	0.55 (0.14)	0.45 (0.09)	0.5 (0.06)	0.5 (0.07)	0.5 (0.06)
O1	0.53 (0.07)	0.56 (0.07)	0.51 (0.17)	0.55 (0.07)	0.51 (0.08)	0.53 (0.07)
O2	0.57 (0.04)	0.61 (0.14)	0.52 (0.06)	0.57 (0.03)	0.59 (0.09)	0.57 (0.04)

F7	0.55 (0.04)	0.59 (0.05)	0.52 (0.09)	0.56 (0.04)	0.55 (0.04)	0.55 (0.04)
F8	0.58 (0.06)	0.65 (0.13)	0.51 (0.04)	0.57 (0.05)	0.6 (0.09)	0.58 (0.06)
T3	0.49 (0.12)	0.45 (0.12)	0.52 (0.16)	0.5 (0.12)	0.48 (0.12)	0.49 (0.12)
T4	0.52 (0.1)	0.57 (0.07)	0.47 (0.15)	0.53 (0.1)	0.51 (0.1)	0.52 (0.1)
T5	0.53 (0.06)	0.51 (0.17)	0.55 (0.06)	0.52 (0.06)	0.53 (0.06)	0.53 (0.06)
T6	0.47 (0.05)	0.47 (0.11)	0.48 (0.04)	0.47 (0.05)	0.47 (0.06)	0.47 (0.05)
A1	0.59 (0.12)	0.65 (0.15)	0.52 (0.11)	0.58 (0.11)	0.61 (0.14)	0.59 (0.12)
A2	0.52 (0.04)	0.49 (0.17)	0.55 (0.12)	0.52 (0.04)	0.52 (0.05)	0.52 (0.04)
FZ	0.42 (0.06)	0.48 (0.1)	0.36 (0.2)	0.44 (0.05)	0.37 (0.1)	0.42 (0.06)
CZ	0.59 (0.07)	0.61 (0.15)	0.57 (0.13)	0.6 (0.07)	0.6 (0.07)	0.59 (0.07)
PZ	0.47 (0.09)	0.51 (0.21)	0.42 (0.06)	0.45 (0.09)	0.48 (0.14)	0.47 (0.09)

VII. Appropriateness of the classifier to represent the feature space

To test whether another classifier would be more appropriate to represent the feature space, analyses were repeated implementing a Random Forest classifier. Results were largely comparable to those observed when implementing SVM (Supplementary Table 25), suggesting that the type of classifier implemented is not a major determinant of the poor performance observed.

Supplementary Table 25. Post-hoc Random Forest analysis on the first random segment. Random Forest was implemented instead of SVM as a classification method. The rest of the pipeline remained unchanged.

Channel	Accuracy mean (SD)	Sensitivity mean (SD)	Specificity mean (SD)	PPV mean (SD)	NPV mean (SD)	AUC mean (SD)
FP1	0.55 (0.03)	0.55 (0.18)	0.55 (0.19)	0.57 (0.06)	0.55 (0.04)	0.55 (0.03)
FP2	0.59 (0.09)	0.57 (0.16)	0.62 (0.09)	0.6 (0.11)	0.6 (0.09)	0.6 (0.09)
F3	0.54 (0.1)	0.55 (0.13)	0.53 (0.11)	0.55 (0.09)	0.54 (0.12)	0.54 (0.1)
F4	0.56 (0.12)	0.64 (0.14)	0.48 (0.2)	0.57 (0.12)	0.55 (0.14)	0.56 (0.12)
C3	0.6 (0.09)	0.68 (0.07)	0.51 (0.17)	0.6 (0.1)	0.59 (0.08)	0.59 (0.09)
C4	0.57 (0.07)	0.57 (0.1)	0.56 (0.07)	0.57 (0.07)	0.57 (0.07)	0.57 (0.07)
P3	0.55 (0.08)	0.65 (0.05)	0.45 (0.12)	0.55 (0.07)	0.55 (0.1)	0.55 (0.08)
P4	0.49 (0.07)	0.55 (0.09)	0.44 (0.08)	0.5 (0.07)	0.48 (0.07)	0.49 (0.07)
O1	0.57 (0.08)	0.53 (0.04)	0.6 (0.14)	0.59 (0.1)	0.55 (0.07)	0.57 (0.08)
O2	0.57 (0.04)	0.61 (0.09)	0.52 (0.1)	0.57 (0.04)	0.57 (0.04)	0.57 (0.04)
F7	0.53 (0.03)	0.59 (0.05)	0.48 (0.06)	0.54 (0.04)	0.53 (0.03)	0.53 (0.03)

F8	0.56 (0.09)	0.63 (0.13)	0.49 (0.17)	0.57 (0.08)	0.56 (0.13)	0.56 (0.09)
T3	0.53 (0.1)	0.55 (0.17)	0.51 (0.1)	0.53 (0.09)	0.54 (0.12)	0.53 (0.1)
T4	0.52 (0.05)	0.56 (0.14)	0.48 (0.06)	0.52 (0.05)	0.53 (0.06)	0.52 (0.05)
T5	0.52 (0.05)	0.53 (0.15)	0.51 (0.12)	0.52 (0.06)	0.52 (0.05)	0.52 (0.05)
T6	0.48 (0.06)	0.47 (0.04)	0.49 (0.13)	0.49 (0.07)	0.47 (0.07)	0.48 (0.07)
A1	0.56 (0.1)	0.59 (0.17)	0.54 (0.11)	0.56 (0.1)	0.57 (0.11)	0.56 (0.1)
A2	0.47 (0.06)	0.48 (0.03)	0.47 (0.1)	0.48 (0.05)	0.46 (0.07)	0.47 (0.06)
FZ	0.48 (0.06)	0.57 (0.07)	0.38 (0.15)	0.5 (0.06)	0.45 (0.09)	0.48 (0.07)
CZ	0.57 (0.06)	0.56 (0.13)	0.59 (0.06)	0.58 (0.05)	0.57 (0.07)	0.57 (0.05)
PZ	0.49 (0.06)	0.51 (0.14)	0.47 (0.08)	0.49 (0.05)	0.49 (0.08)	0.49 (0.06)

VIII. Low dimensional visualisation

Projection of the high-dimensional feature space onto a two-dimensional space was performed by means of t-SNE to allow visualisation of neighbours and clusters. Supplementary Figure 12 depicts 2D t-SNE projections for three sample channels. From the graphs, there are no evident clusters corresponding to the diagnoses of epilepsy or PNES (Supplementary Figure 12a). A marginally higher degree of clustering can be seen when labelling observations by EEG outcome; patients that had an overall abnormal EEG investigation tended to group together in more peripheral locations in certain channels (Supplementary Figure 12b).

Supplementary Figure 12. Two-dimensional t-SNE visualisations of the feature space structure for three sample channels. (a) labelled by diagnostic group; (b) labelled by EEG outcome; x axis: first dimension created by t-SNE; y axis: second t-SNE dimension.

(a)

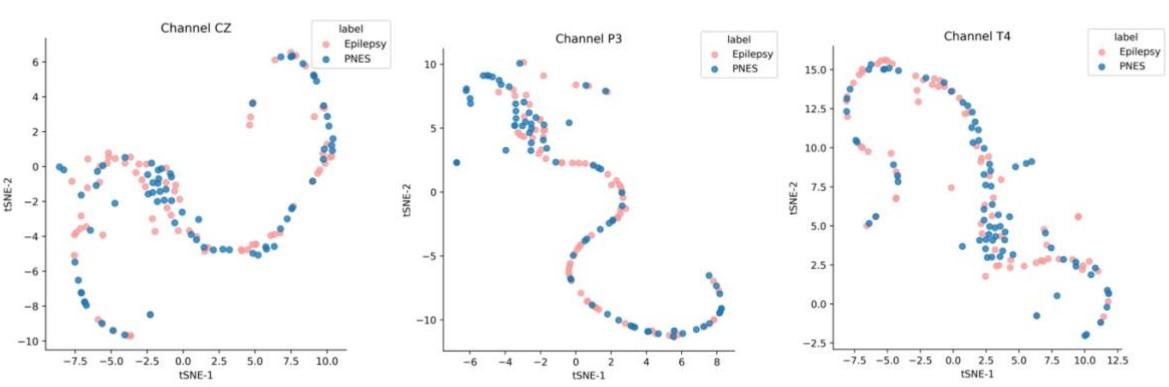

(b)

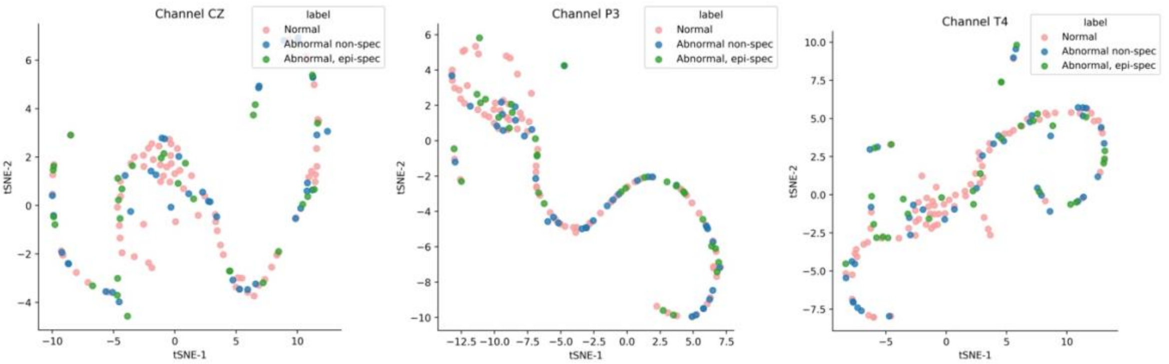

Subset analyses results

Supplementary Table 26. Subset analyses results for selected features in 21 channels for the first randomly sampled EEG segment.

Channel	Subset	Accuracy	Sensitivity	Specificity	PPV	NPV	AUC
**FP1**	vEEG confirmation	0.53	0.53	0.53	0.46	0.61	0.53
	No vEEG confirmation	0.49	0.5	0.47	0.67	0.3	0.48
	Focal epilepsy	0.49	0.42	0.52	0.29	0.67	0.47
	Generalised epilepsy	0.53	0.55	0.52	0.24	0.81	0.54
	Unclassified epilepsy	0.55	0.64	0.52	0.29	0.83	0.58
	EEG Normal	0.54	0.5	0.56	0.31	0.74	0.53
	EEG Abnormal, non-specific	0.41	0.48	0.29	0.55	0.24	0.38
	EEG Abnormal, epilepsy-specific	0.6	0.57	1.0	1.0	0.14	0.79
**FP2**	vEEG confirmation	0.52	0.49	0.55	0.45	0.59	0.52
	No vEEG confirmation	0.6	0.62	0.53	0.74	0.4	0.58
	Focal epilepsy	0.54	0.52	0.55	0.34	0.71	0.53
	Generalised epilepsy	0.54	0.5	0.55	0.23	0.8	0.52
	Unclassified epilepsy	0.57	0.64	0.55	0.3	0.83	0.59
	EEG Normal	0.53	0.5	0.54	0.3	0.74	0.52
	EEG Abnormal, non-specific	0.62	0.6	0.64	0.75	0.47	0.62
	EEG Abnormal, epilepsy-specific	0.5	0.54	0.0	0.88	0.0	0.27
**F3**	vEEG confirmation	0.57	0.63	0.53	0.5	0.66	0.58
	No vEEG confirmation	0.64	0.59	0.73	0.83	0.46	0.66
	Focal epilepsy	0.58	0.58	0.58	0.38	0.75	0.58
	Generalised epilepsy	0.6	0.7	0.58	0.31	0.88	0.64

	Unclassified epilepsy	0.58	0.59	0.58	0.3	0.82	0.58
	EEG Normal	0.61	0.59	0.61	0.37	0.8	0.6
	EEG Abnormal, non-specific	0.54	0.64	0.36	0.64	0.36	0.5
	EEG Abnormal, epilepsy-specific	0.63	0.61	1.0	1.0	0.15	0.8
**F4**	vEEG confirmation	0.53	0.65	0.45	0.47	0.63	0.55
	No vEEG confirmation	0.57	0.66	0.4	0.7	0.35	0.53
	Focal epilepsy	0.48	0.58	0.44	0.32	0.7	0.51
	Generalised epilepsy	0.51	0.75	0.44	0.27	0.86	0.59
	Unclassified epilepsy	0.49	0.68	0.44	0.27	0.82	0.56
	EEG Normal	0.52	0.68	0.46	0.33	0.79	0.57
	EEG Abnormal, non-specific	0.56	0.68	0.36	0.65	0.38	0.52
	EEG Abnormal, epilepsy-specific	0.6	0.61	0.5	0.94	0.08	0.55
**C3**	vEEG confirmation	0.57	0.6	0.55	0.5	0.65	0.58
	No vEEG confirmation	0.66	0.72	0.53	0.77	0.47	0.63
	Focal epilepsy	0.55	0.55	0.55	0.35	0.73	0.55
	Generalised epilepsy	0.57	0.65	0.55	0.28	0.85	0.6
	Unclassified epilepsy	0.61	0.82	0.55	0.35	0.91	0.68
	EEG Normal	0.58	0.68	0.54	0.37	0.82	0.61
	EEG Abnormal, non-specific	0.69	0.76	0.57	0.76	0.57	0.67
	EEG Abnormal, epilepsy-specific	0.53	0.54	0.5	0.94	0.07	0.52
**C4**	vEEG confirmation	0.53	0.51	0.55	0.46	0.6	0.53
	No vEEG confirmation	0.55	0.62	0.4	0.69	0.33	0.51
	Focal epilepsy	0.51	0.48	0.52	0.31	0.69	0.5
	Generalised epilepsy	0.56	0.7	0.52	0.29	0.86	0.61
	Unclassified epilepsy	0.53	0.55	0.52	0.26	0.79	0.53
	EEG Normal	0.53	0.55	0.53	0.31	0.75	0.54
	EEG Abnormal, non-specific	0.62	0.68	0.5	0.71	0.47	0.59
	EEG Abnormal, epilepsy-specific	0.47	0.46	0.5	0.93	0.06	0.48
**P3**	vEEG confirmation	0.54	0.72	0.41	0.48	0.67	0.57
	No vEEG confirmation	0.6	0.59	0.6	0.76	0.41	0.6
	Focal epilepsy	0.53	0.7	0.45	0.37	0.77	0.57
	Generalised epilepsy	0.51	0.7	0.45	0.26	0.85	0.58
	Unclassified epilepsy	0.48	0.59	0.45	0.25	0.79	0.52

	EEG Normal	0.53	0.68	0.47	0.33	0.79	0.58
	EEG Abnormal, non-specific	0.51	0.56	0.43	0.64	0.35	0.49
	EEG Abnormal, epilepsy-specific	0.7	0.75	0.0	0.91	0.0	0.38
**P4**	vEEG confirmation	0.51	0.51	0.52	0.44	0.59	0.51
	No vEEG confirmation	0.47	0.5	0.4	0.64	0.27	0.45
	Focal epilepsy	0.49	0.48	0.49	0.3	0.68	0.49
	Generalised epilepsy	0.52	0.6	0.49	0.24	0.82	0.55
	Unclassified epilepsy	0.48	0.45	0.49	0.21	0.75	0.47
	EEG Normal	0.49	0.59	0.46	0.3	0.74	0.52
	EEG Abnormal, non-specific	0.49	0.4	0.64	0.67	0.38	0.52
	EEG Abnormal, epilepsy-specific	0.53	0.54	0.5	0.94	0.07	0.52
**O1**	vEEG confirmation	0.54	0.58	0.52	0.47	0.62	0.55
	No vEEG confirmation	0.51	0.47	0.6	0.71	0.35	0.53
	Focal epilepsy	0.5	0.42	0.53	0.29	0.67	0.48
	Generalised epilepsy	0.55	0.6	0.53	0.26	0.83	0.57
	Unclassified epilepsy	0.56	0.64	0.53	0.29	0.83	0.59
	EEG Normal	0.54	0.55	0.54	0.32	0.76	0.54
	EEG Abnormal, non-specific	0.49	0.44	0.57	0.65	0.36	0.51
	EEG Abnormal, epilepsy-specific	0.57	0.61	0.0	0.89	0.0	0.3
**O2**	vEEG confirmation	0.56	0.53	0.59	0.49	0.63	0.56
	No vEEG confirmation	0.55	0.59	0.47	0.7	0.35	0.53
	Focal epilepsy	0.58	0.61	0.56	0.38	0.76	0.58
	Generalised epilepsy	0.56	0.55	0.56	0.26	0.82	0.56
	Unclassified epilepsy	0.55	0.5	0.56	0.26	0.79	0.53
	EEG Normal	0.56	0.45	0.6	0.3	0.74	0.53
	EEG Abnormal, non-specific	0.56	0.6	0.5	0.68	0.41	0.55
	EEG Abnormal, epilepsy-specific	0.57	0.61	0.0	0.89	0.0	0.3
**F7**	vEEG confirmation	0.55	0.6	0.52	0.48	0.64	0.56
	No vEEG confirmation	0.51	0.53	0.47	0.68	0.32	0.5
	Focal epilepsy	0.55	0.64	0.51	0.37	0.76	0.57
	Generalised epilepsy	0.52	0.55	0.51	0.23	0.8	0.53
	Unclassified epilepsy	0.51	0.5	0.51	0.23	0.77	0.5
	EEG Normal	0.57	0.64	0.54	0.35	0.79	0.59

	EEG Abnormal, non-specific	0.44	0.48	0.36	0.57	0.28	0.42
	EEG Abnormal, epilepsy-specific	0.6	0.61	0.5	0.94	0.08	0.55
**F8**	vEEG confirmation	0.59	0.74	0.48	0.52	0.72	0.61
	No vEEG confirmation	0.53	0.5	0.6	0.73	0.36	0.55
	Focal epilepsy	0.58	0.76	0.51	0.41	0.82	0.63
	Generalised epilepsy	0.49	0.45	0.51	0.2	0.77	0.48
	Unclassified epilepsy	0.54	0.64	0.51	0.28	0.82	0.57
	EEG Normal	0.56	0.55	0.56	0.32	0.76	0.55
	EEG Abnormal, non-specific	0.49	0.56	0.36	0.61	0.31	0.46
	EEG Abnormal, epilepsy-specific	0.73	0.79	0.0	0.92	0.0	0.39
**T3**	vEEG confirmation	0.5	0.56	0.45	0.43	0.58	0.5
	No vEEG confirmation	0.51	0.41	0.73	0.76	0.37	0.57
	Focal epilepsy	0.51	0.52	0.51	0.32	0.7	0.51
	Generalised epilepsy	0.49	0.45	0.51	0.2	0.77	0.48
	Unclassified epilepsy	0.51	0.5	0.51	0.23	0.77	0.5
	EEG Normal	0.46	0.18	0.56	0.14	0.64	0.37
	EEG Abnormal, non-specific	0.56	0.68	0.36	0.65	0.38	0.52
	EEG Abnormal, epilepsy-specific	0.53	0.57	0.0	0.89	0.0	0.29
**T4**	vEEG confirmation	0.52	0.6	0.47	0.46	0.61	0.54
	No vEEG confirmation	0.6	0.62	0.53	0.74	0.4	0.58
	Focal epilepsy	0.5	0.55	0.48	0.32	0.7	0.51
	Generalised epilepsy	0.54	0.75	0.48	0.28	0.88	0.61
	Unclassified epilepsy	0.51	0.59	0.48	0.25	0.8	0.54
	EEG Normal	0.48	0.45	0.49	0.26	0.7	0.47
	EEG Abnormal, non-specific	0.56	0.68	0.36	0.65	0.38	0.52
	EEG Abnormal, epilepsy-specific	0.7	0.68	1.0	1.0	0.18	0.84
**T5**	vEEG confirmation	0.53	0.56	0.52	0.46	0.61	0.54
	No vEEG confirmation	0.55	0.56	0.53	0.72	0.36	0.55
	Focal epilepsy	0.51	0.48	0.52	0.31	0.69	0.5
	Generalised epilepsy	0.54	0.6	0.52	0.26	0.83	0.56
	Unclassified epilepsy	0.55	0.64	0.52	0.29	0.83	0.58
	EEG Normal	0.49	0.5	0.49	0.28	0.72	0.5
	EEG Abnormal, non-specific	0.59	0.6	0.57	0.71	0.44	0.59

	EEG Abnormal, epilepsy-specific	0.6	0.57	1.0	1.0	0.14	0.79
**T6**	vEEG confirmation	0.45	0.56	0.36	0.39	0.52	0.46
	No vEEG confirmation	0.49	0.41	0.67	0.72	0.34	0.54
	Focal epilepsy	0.46	0.55	0.42	0.3	0.67	0.49
	Generalised epilepsy	0.42	0.4	0.42	0.16	0.72	0.41
	Unclassified epilepsy	0.44	0.5	0.42	0.21	0.74	0.46
	EEG Normal	0.47	0.45	0.47	0.25	0.69	0.46
	EEG Abnormal, non-specific	0.38	0.48	0.21	0.52	0.19	0.35
	EEG Abnormal, epilepsy-specific	0.53	0.54	0.5	0.94	0.07	0.52
**A1**	vEEG confirmation	0.55	0.58	0.53	0.48	0.63	0.56
	No vEEG confirmation	0.7	0.69	0.73	0.85	0.52	0.71
	Focal epilepsy	0.58	0.58	0.58	0.38	0.75	0.58
	Generalised epilepsy	0.62	0.8	0.58	0.34	0.91	0.69
	Unclassified epilepsy	0.57	0.55	0.58	0.28	0.81	0.56
	EEG Normal	0.65	0.73	0.61	0.42	0.85	0.67
	EEG Abnormal, non-specific	0.49	0.52	0.43	0.62	0.33	0.47
	EEG Abnormal, epilepsy-specific	0.63	0.64	0.5	0.95	0.09	0.57
**A2**	vEEG confirmation	0.51	0.56	0.48	0.44	0.6	0.52
	No vEEG confirmation	0.66	0.59	0.8	0.86	0.48	0.7
	Focal epilepsy	0.54	0.52	0.55	0.34	0.71	0.53
	Generalised epilepsy	0.57	0.65	0.55	0.28	0.85	0.6
	Unclassified epilepsy	0.56	0.59	0.55	0.28	0.82	0.57
	EEG Normal	0.56	0.55	0.56	0.32	0.76	0.55
	EEG Abnormal, non-specific	0.62	0.64	0.57	0.73	0.47	0.61
	EEG Abnormal, epilepsy-specific	0.5	0.54	0.0	0.88	0.0	0.27
**FZ**	vEEG confirmation	0.45	0.58	0.34	0.4	0.53	0.46
	No vEEG confirmation	0.45	0.47	0.4	0.62	0.26	0.43
	Focal epilepsy	0.41	0.52	0.36	0.27	0.62	0.44
	Generalised epilepsy	0.4	0.55	0.36	0.19	0.74	0.45
	Unclassified epilepsy	0.4	0.55	0.36	0.2	0.72	0.45
	EEG Normal	0.39	0.41	0.39	0.2	0.63	0.4
	EEG Abnormal, non-specific	0.51	0.64	0.29	0.62	0.31	0.46
	EEG Abnormal, epilepsy-specific	0.5	0.54	0.0	0.88	0.0	0.27

**CZ**	vEEG confirmation	0.6	0.65	0.57	0.53	0.69	0.61
	No vEEG confirmation	0.53	0.56	0.47	0.69	0.33	0.51
	Focal epilepsy	0.54	0.52	0.55	0.34	0.71	0.53
	Generalised epilepsy	0.61	0.85	0.55	0.34	0.93	0.7
	Unclassified epilepsy	0.55	0.55	0.55	0.27	0.8	0.55
	EEG Normal	0.56	0.59	0.54	0.33	0.78	0.57
	EEG Abnormal, non-specific	0.56	0.56	0.57	0.7	0.42	0.57
	EEG Abnormal, epilepsy-specific	0.67	0.68	0.5	0.95	0.1	0.59
**PZ**	vEEG confirmation	0.5	0.67	0.36	0.44	0.6	0.52
	No vEEG confirmation	0.49	0.47	0.53	0.68	0.32	0.5
	Focal epilepsy	0.44	0.55	0.4	0.29	0.66	0.47
	Generalised epilepsy	0.47	0.75	0.4	0.25	0.85	0.57
	Unclassified epilepsy	0.42	0.5	0.4	0.2	0.72	0.45
	EEG Normal	0.42	0.41	0.42	0.21	0.65	0.42
	EEG Abnormal, non-specific	0.44	0.52	0.29	0.57	0.25	0.4
	EEG Abnormal, epilepsy-specific	0.77	0.79	0.5	0.96	0.14	0.64

Section 4: SVM code for Study 1

```
# # STUDY 1 - hypothesis-driven: Power and PAF - SVM analyses

# ## 1. Import dataset

# In[62]:



# Read .csv file (as data frame) – this is the file only containing data on the two groups of interest (E=epilepsy and P=PNES).
import pandas as pd
df = pd.read_csv(r'/Volumes/Ashur DT2/Power analysis/MasterFile_EP.csv')
df



# ## 2. Pick 3 random segments

# ### Create indices to pick 3 random segments

# In[3]:



# Set index (row labels)
df.set_index('SubjID', inplace=True)

# Change SgmtNr for RKS107 to 1 (other segments were not included in the analysis for this patient because they had no detectable alpha peak on a minimum of three channels. See paper's methods section: "EEG segments without a detectable alpha peak on a minimum of three channels (as detected by the restingIAF function) were excluded from further analyses").
df.at['RKS107', 'SgmtNr'] = 1

# Create a new column containing within-subject-permuted segment number (RandomState added so permutation order is reproducible)
import numpy as np
df['Permuted_SgmtNr'] =
df.groupby('SubjID')['SgmtNr'].transform(np.random.RandomState(seed=42).permutation)
```

```
# Create a 2nd new column containing indices for picking permuted segment #2, or segment #1 if #2 does not exist
Permuted2 = list()
groups = df.groupby('SubjNr') # by SubjNr to maintain the order (SubjID gets reorganised alphabetically and messes up the indexing)
for name, group in groups:
    if 2 in group['Permuted_SgmtNr'].values:
        group['Permuted2_SgmtNr'] = np.where(group['Permuted_SgmtNr'] == 2, 1, 0)
    else:
        group['Permuted2_SgmtNr'] = np.where(group['Permuted_SgmtNr'] == 1, 1, 0)
    Permuted2.append(group['Permuted2_SgmtNr'])
concatenated2 = []
for l in Permuted2:
    concatenated2.extend(l)
df['Permuted2_SgmtNr'] = concatenated2

# Create a 3rd new column containing indices for picking permuted segment #3, or segment #2 if #3 does not exist, or #1 if #2 does not exist
Permuted3 = list()
groups = df.groupby('SubjNr')
for name, group in groups:
    if 3 in group['Permuted_SgmtNr'].values:
        group['Permuted3_SgmtNr'] = np.where(group['Permuted_SgmtNr'] == 3, 1, 0)
    elif 2 in group['Permuted_SgmtNr'].values:
        group['Permuted3_SgmtNr'] = np.where(group['Permuted_SgmtNr'] == 2, 1, 0)
    else:
        group['Permuted3_SgmtNr'] = np.where(group['Permuted_SgmtNr'] == 1, 1, 0)
    Permuted3.append(group['Permuted3_SgmtNr'])
concatenated3 = []
for l in Permuted3:
    concatenated3.extend(l)
df['Permuted3_SgmtNr'] = concatenated3


# ### Pick segments and save

# In[4]:


# Pick permuted segment #1
```

```
rs1 = df[df['Permuted_SgmtNr'] == 1]
rs1


# In[257]:


# Pick permuted segment # 2
rs2 = df[df['Permuted2_SgmtNr'] == 1]
rs2


# In[258]:


# Pick permuted segment # 3
rs3 = df[df['Permuted3_SgmtNr'] == 1]
rs3


# In[ ]:


# Save
rs1.to_csv('/Volumes/Ashur DT2/Power analysis/random_segment1/MasterFile_EP_rs1.csv')
rs2.to_csv('/Volumes/Ashur DT2/Power analysis/random_segment2/MasterFile_EP_rs2.csv')
rs3.to_csv('/Volumes/Ashur DT2/Power analysis/random_segment3/MasterFile_EP_rs3.csv')


# ## 3. Define predictor pool and y vector

# In[3]:


# Import dataset
import pandas as pd
rs1 = pd.read_csv(r'/Volumes/Ashur DT2/Power analysis/random_segment1/MasterFile_EP_rs1.csv') # Note that _rs1 is imported here and used as the main EEG segment analysed. The main results reported in the paper relate to this segment. You can choose to import _rs2.csv or _rs3.csv here to run control analyses on the two different randomly selected 20s segments per participant.
```

```
rs1.set_index('SubjID', inplace=True)
rs1


# In[44]:


# Define predictor matrix (X) and predicted variable (y)
import numpy as np
idx = np.r_[26:47, 110:111] # np.r_[26:47, 110:111] = main hypotheis (theta and PAF)
X = rs1.iloc[:,idx]
y = rs1.iloc[:,124] # diagnosis_at_analysis
# Check if correct columns selected
X


#
# ## 4. Split in Kfold, scale (z-transform) the data, run SVM with RBF kernel

# ### SPLIT: Split dataset into K folds stratified by y, so the proportion of y=0 and y=1 is balanced in the test set.
# #### StratifiedKFold takes group information into account to avoid building folds with imbalanced class distributions
# #### Kfold CV guarantees that the results of the model do not depend on the way we picked the train and test set
#
# ### NORMALIZATION:  Use StandardScaler (z-transform) to create scale values based on X_train and retain info to then scale X_test based on same parameters
#
# ### HYPERPARAMETERS ESTIMATION: Use Grid Search and CV to find the best parameters C and γ

# In[45]:


# Import stratified Kfold split function
import numpy as np
from sklearn.model_selection import StratifiedKFold
#Import function to standardize the data
from sklearn import preprocessing
#Import functions for svm and cv
from sklearn import svm
```

```
from sklearn.svm import SVC
from sklearn.model_selection import GridSearchCV
from sklearn.metrics import classification_report
from sklearn.metrics import confusion_matrix
from sklearn import metrics
import seaborn as sns
import matplotlib.pyplot as plt

# Prepare lists where to save results
outerloop_acc = list()
outerloop_sens = list()
outerloop_spec = list()
outerloop_PPV = list()
outerloop_NPV = list()
outerloop_AUC = list()
Results = []
FoldNr = 0

# ------------- Split dataset into training set and test set using stratified Kfold (stratifies by y) ---------

# Run stratified k-fold: provides train/test indices to split data in train/test sets
skf = StratifiedKFold(n_splits=5, shuffle=True, random_state=42) # Randomized CV splitters make the results
identical if random_state set to integer (reproducible split).

# Split X and y based on skf indices
for train_index, test_index in skf.split(X, y):
    FoldNr = FoldNr + 1
    print("TRAIN FoldNr:" + str(FoldNr), train_index, "TEST FoldNr:" + str(FoldNr), test_index)
    X_train, X_test = X.iloc[train_index], X.iloc[test_index]
    y_train, y_test = y.iloc[train_index], y.iloc[test_index]
    print("Train X shape: %s, train y shape: %s" % (X_train.shape, y_train.shape))
    print("Test X shape: %s, test y shape: %s" % (X_test.shape, y_test.shape))
    print()

    # --------------------------------------------  Normalize data  --------------------------------------------

    # Use StandardScaler (z-transform) to create scale values based on X_train and retain info to then scale
X_test based on same parameters
    scaler = preprocessing.StandardScaler().fit(X_train) # create scaler based on training set (scaler fit to train set)
    X_train = scaler.transform(X_train) # scale train set
```

```
X_test = scaler.transform (X_test) # scale test set

# Check that X_train mean = 0
X_train.mean(axis=0)
# Check that X_train sd = 1
X_train.std(axis=0)

# --------------  Find best hyperparameters using grid search with cross-validation (on training set) ------------

# Set the parameters by cross-validation
search_parameters = [
    {'C': [0.01, 0.1, 1, 10, 100, 1000, 10000], 'gamma': [1, 0.1, 0.01, 0.001, 0.0001, 0.0001, 0.00001], 'kernel':
['rbf']},
    ]
scores = ["accuracy"]
for score in scores: # Find optimal parameters for each of the scores specified
    print("# Tuning hyper-parameters for %s" % score)
    print()

    # runs SVM on training set with cv to find parameters that best fit to training set (cv is 5-fold by default, and
StratifiedKFold is used by default to stratify by y)
    # GridSearchCV: refitbool default = True -> After cv, refits a final model on the entire training dataset using
the best hyperparameters found during the search
    clf = GridSearchCV(SVC(), search_parameters, scoring="%s" % score)
    clf.fit(X_train, y_train) # fits classifier to training set

    # Parameter settings that gave the best results on the training set following cv
    print("Best parameters set found on training set: %s with a score of %0.2f" % (clf.best_params_,
clf.best_score_))
    print()
    # Print means and sd of classification performance for each parameter combination
    means = clf.cv_results_["mean_test_score"]
    stds = clf.cv_results_["std_test_score"]
    print("Grid scores on training set:")
    print()
    for mean, std, params in zip(means, stds, clf.cv_results_["params"]):
        print("%0.3f (+/-%0.03f) for %r" % (mean, std * 2, params))

    # ---------------------------------  Measure performance on test set  ---------------------------
```

```
    # performance of the selected parameters and trained model is measured on test set
    y_true, y_pred = y_test, clf.predict(X_test)
    cm = confusion_matrix(y_true, y_pred, labels=[3, 4])
    sens = cm[0,0]/(cm[0,0]+cm[0,1]) # tp / (tp+fn)
    spec = cm[1,1]/(cm[1,0]+cm[1,1]) # tn / (fp+tn)
    acc = metrics.accuracy_score(y_true, y_pred)
    PPV = cm[0,0]/(cm[0,0]+cm[1,0]) # tp / (tp+fp)
    NPV = cm[1,1]/(cm[1,1]+cm[0,1]+1e-20) # tn / (tn+fn) + 1e-20 to avoid dividing by zero
    AUC = metrics.roc_auc_score(y_true, y_pred)
    print()
    print('Sensitivity : ', sens)
    print('Specificity : ', spec)
    print('Accuracy: ', acc)
    print("CONFUSION MATRIX (Performance measured on the test set) 0=3(epilepsy); 1=4(PNES):")
    ax = sns.heatmap(cm, annot=True, cmap='Blues')
    ax.set_xlabel('\nPredicted Values')
    ax.set_ylabel('Actual Values ');
    plt.show()
    print()

    # Append results
    outerloop_sens.append(sens)
    outerloop_spec.append(spec)
    outerloop_acc.append(acc)
    outerloop_PPV.append(PPV)
    outerloop_NPV.append(NPV)
    outerloop_AUC.append(AUC)

    # Save column with predicted values for control analyses
    a = pd.DataFrame(y_true).reset_index()
    b = pd.Series(y_pred, name = 'diagnosis_pred')
    globals()['ObsPred_df' + str(FoldNr)] = a.join(b)
    globals()['ObsPred_df' + str(FoldNr)].set_index('SubjID', inplace=True)

# ----------------------------------  Average performance results across K outer folds  ---------------------------

ObsPred_Concat = pd.concat([ObsPred_df1, ObsPred_df2, ObsPred_df3, ObsPred_df4, ObsPred_df5]) # merge y_pred from all 5 folds
rs1['diagnosis_pred'] = ObsPred_Concat['diagnosis_pred'] # map y_pred values onto original matrix for control analyses
```

```
print('Mean accuracy score across ' + str(FoldNr) + ' outer Folds: %.3f (%.3f); Minimum: %.3f; Maximum: %.3f' % (np.mean(outerloop_acc), np.std(outerloop_acc), np.min(outerloop_acc), (np.max(outerloop_acc))))
print ('Mean sensitivity score across ' + str(FoldNr) + ' outer Folds: %.3f (%.3f); Minimum: %.3f; Maximum: %.3f' % (np.mean(outerloop_sens), np.std(outerloop_sens), np.min(outerloop_sens), np.max(outerloop_sens)))
print ('Mean specificity score across ' + str(FoldNr) + ' outer Folds: %.3f (%.3f); Minimum: %.3f; Maximum: %.3f' % (np.mean(outerloop_spec), np.std(outerloop_spec), np.min(outerloop_spec), np.max(outerloop_spec)))
print ('Mean PPV score across ' + str(FoldNr) + ' outer Folds: %.3f (%.3f); Minimum: %.3f; Maximum: %.3f' % (np.mean(outerloop_PPV), np.std(outerloop_PPV), np.min(outerloop_PPV), np.max(outerloop_PPV)))
print ('Mean NPV score across ' + str(FoldNr) + ' outer Folds: %.3f (%.3f); Minimum: %.3f; Maximum: %.3f' % (np.mean(outerloop_NPV), np.std(outerloop_NPV), np.min(outerloop_NPV), np.max(outerloop_NPV)))
print ('Mean AUC score across ' + str(FoldNr) + ' outer Folds: %.3f (%.3f); Minimum: %.3f; Maximum: %.3f' % (np.mean(outerloop_AUC), np.std(outerloop_AUC), np.min(outerloop_AUC), np.max(outerloop_AUC)))

# --------------------------------- Format for export -----------------------
Results.append(
   {
      'Accuracy_Mean': np.mean(outerloop_acc),
      'Accuracy_SD':  np.std(outerloop_acc),
      'Sensitivity_Mean':  np.mean(outerloop_sens),
      'Sensitivity_SD':  np.std(outerloop_sens),
      'Specificity_Mean':   np.mean(outerloop_spec),
      'Specificity_SD':  np.std(outerloop_spec),
      'PPV_Mean':   np.mean(outerloop_PPV),
      'PPV_SD':  np.std(outerloop_PPV),
      'NPV_Mean':   np.mean(outerloop_NPV),
      'NPV_SD':  np.std(outerloop_NPV),
      'AUC_Mean':   np.mean(outerloop_AUC),
      'AUC_SD':  np.std(outerloop_AUC),
   }
)
```

Section 5: SVM code for Study 2

```
#!/usr/bin/env python
# coding: utf-8

# In[ ]:


from platform import python_version
python_version()


# # STUDY 2 - hctsa - SVM analyses on hctsa dataset


# ## 1. Define predictor pool and y vector

# In[2]:


# Import dataset
import pandas as pd
# channels = ['FP1','FP2','F3','F4','C3','C4','P3','P4','O1','O2','F7','F8','T3','T4','T5','T6','A1','A2','FZ','CZ','PZ']
channels = ['Cz'] # SELECT ONE CHANNEL AT A TIME
for ch in channels:
    rs1 = pd.read_csv(r'/Volumes/Ashur DT2/hctsa 
analysis/random_segment1/hctsa_results/RS1_'+ch+'_hctsa_MasterFile_EP.csv')
    rs1.set_index('SubjID', inplace=True)
rs1


# In[105]:


# Define predictor matrix (X) and predicted variable (y)
import numpy as np
idx = np.r_[19:6444] # select all predictors
X = rs1.iloc[:,idx]
y = rs1.iloc[:,9] # diagnosis_at_analysis
```

```
X # Check if correct columns selected


# ## 2. SVM with FEATURE SELECTION

# In[106]:


# Import stratified Kfold split function
import numpy as np
from sklearn.model_selection import StratifiedKFold
#Import function to standardize the data
from sklearn import preprocessing
#Import functions for svm and cv
from sklearn import svm
from sklearn.svm import SVC
from sklearn.model_selection import GridSearchCV
from sklearn.metrics import classification_report
from sklearn.metrics import confusion_matrix
from sklearn import metrics
import seaborn as sns
import matplotlib.pyplot as plt
# Import feature selection functions
from mrmr import mrmr_classif
import pingouin as pg
import numpy as np
# Import functions for comparing features
from collections import Counter

# Prepare lists where to save results
mrmr_features = list()
mrmr_features_names = list()
outerloop_acc = list()
outerloop_sens = list()
outerloop_spec = list()
outerloop_PPV = list()
outerloop_NPV = list()
outerloop_AUC = list()
Results = []
FoldNr = 0
```

```
# ------------- Split dataset into training set and test set using stratified Kfold (stratifies by y) ---------

# Run stratified k-fold: provides train/test indices to split data in train/test sets
skf = StratifiedKFold(n_splits=5, shuffle=True, random_state=42) # Randomized CV splitters make the results identical if random_state set to integer (reproducible split).

# Split X and y based on skf indices
for train_index, test_index in skf.split(X, y):
    FoldNr = FoldNr + 1
    print("TRAIN FoldNr:" + str(FoldNr), train_index, "TEST FoldNr:" + str(FoldNr), test_index)
    X_train, X_test = X.iloc[train_index], X.iloc[test_index]
    y_train, y_test = y.iloc[train_index], y.iloc[test_index]
    print("Train X shape: %s, train y shape: %s" % (X_train.shape, y_train.shape))
    print("Test X shape: %s, test y shape: %s" % (X_test.shape, y_test.shape))
    print()

    # ------------------------------  Feature Selection  (on training set) ---------------------------------------

    # Find K-best features based on minimum Redundancy - Maximum Relevance
    mrmr_feat = mrmr_classif(X_train, y_train, K = 25) # start with best 25 features

    # Based on r squared, find nr of features that explain at least 75% of the variance in y
    r2_list = []
    for f in list(range(0,25)): # for each feature
        corr = pg.corr(X_train[mrmr_feat[f]], y_train) # compute correlation r
        r2 = corr['r']*corr['r'] # compute r squared
        r2_list.append(r2)
    r2_cumsum = np.cumsum(r2_list) # compute cumulative sum of explained variance
    more075 = np.where(r2_cumsum > 0.75)
    idx075 = [item[0] for item in more075] # identify number of features needed to explaing at least 75% of the variance in y.

    # Generate list of selected features to use as model's predictors
    mrmr_feat_idx = [X_train.columns.get_loc(c) for c in mrmr_feat[0:idx075[0]+1] if c in X] # +1 to correct for zero indexing
    mrmr_features.append(mrmr_feat_idx) # Store indices of selected features
    mrmr_features_names.append(mrmr_feat[0:idx075[0]+1]) # # Store names of selected features
    print('Features selected in FoldNr '+ str(FoldNr) +': '+ str(idx075[0]+1))
```

```
# Reduce feature sets to the selected features only
X_train = X_train.iloc[:,mrmr_feat_idx]
X_test = X_test.iloc[:,mrmr_feat_idx]
print("Train X shape after feature selection: %s, train y shape: %s" % (X_train.shape, y_train.shape))
print("Test X shape after feature selection: %s, test y shape: %s" % (X_test.shape, y_test.shape))
print()

# --------------------------------------------  Normalize data  --------------------------------------------

# Use StandardScaler (z-transform) to create scale values based on X_train and retain info to then scale
X_test based on same parameters
scaler = preprocessing.StandardScaler().fit(X_train) # create scaler based on training set (scaler fit to train set)
X_train = scaler.transform(X_train) # scale train set
X_test = scaler.transform (X_test) # scale test set

# Check that X_train mean = 0
X_train.mean(axis=0)
# Check that X_train sd = 1
X_train.std(axis=0)

# -------------  Find best hyperparameters using grid search with cross-validation (on training set) ------------

# Set the parameters by cross-validation
search_parameters = [
    {'C': [0.01, 0.1, 1, 10, 100, 1000, 10000], 'gamma': [1, 0.1, 0.01, 0.001, 0.0001, 0.0001, 0.00001], 'kernel':
['rbf']},
    ]
scores = ["accuracy"]
for score in scores: # Find optimal parameters for each of the scores specified
    print("# Tuning hyper-parameters for %s" % score)
    print()

    # runs SVM on training set with cv to find parameters that best fit to training set (cv is 5-fold by default)
    # GridSearchCV: refitbool default = True -> After cv, refits a final model on the entire training dataset using
the best hyperparameters found during the search
    clf = GridSearchCV(SVC(), search_parameters, scoring="%s" % score)
    clf.fit(X_train, y_train) # fits classifier to training set

    # Parameter settings that gave the best results on the training set following cv
```

```
    print("Best parameters set found on training set: %s with a score of %0.2f" % (clf.best_params_,
clf.best_score_))
    print()
    # Print means and sd of classification performance for each parameter combination
    means = clf.cv_results_["mean_test_score"]
    stds = clf.cv_results_["std_test_score"]
    print("Grid scores on training set:")
    print()
    for mean, std, params in zip(means, stds, clf.cv_results_["params"]):
        print("%0.3f (+/-%0.03f) for %r" % (mean, std * 2, params))

    # ----------------------------------  Measure performance on test set  ----------------------------

    # performance of the selected parameters and trained model is measured on test set
    y_true, y_pred = y_test, clf.predict(X_test)
    cm = confusion_matrix(y_true, y_pred, labels=[3, 4])
    sens = cm[0,0]/(cm[0,0]+cm[0,1]) # tp / (tp+fn)
    spec = cm[1,1]/(cm[1,0]+cm[1,1]) # tn / (fp+tn)
    acc = metrics.accuracy_score(y_true, y_pred)
    PPV = cm[0,0]/(cm[0,0]+cm[1,0]) # tp / (tp+fp)
    NPV = cm[1,1]/(cm[1,1]+cm[0,1]+1e-20) # tn / (tn+fn) + 1e-20 to avoid dividing by zero
    AUC = metrics.roc_auc_score(y_true, y_pred)
    print()
    print('Sensitivity : ', sens)
    print('Specificity : ', spec)
    print('Accuracy: ', acc)
    print("CONFUSION MATRIX (Performance measured on the test set) 0=3(epilepsy); 1=4(PNES):")
    ax = sns.heatmap(cm, annot=True, cmap='Blues')
    ax.set_xlabel('\nPredicted Values')
    ax.set_ylabel('Actual Values ');
    plt.show()
    print()

    # Append results
    outerloop_sens.append(sens)
    outerloop_spec.append(spec)
    outerloop_acc.append(acc)
    outerloop_PPV.append(PPV)
    outerloop_NPV.append(NPV)
    outerloop_AUC.append(AUC)
```

```
        # Save column with predicted values for control analyses
        a = pd.DataFrame(y_true).reset_index()
        b = pd.Series(y_pred, name = 'diagnosis_pred')
        globals()['ObsPred_df' + str(FoldNr)] = a.join(b)
        globals()['ObsPred_df' + str(FoldNr)].set_index('SubjID', inplace=True)

# ---------------------------------  Average performance results across K outer folds  ---------------------------

ObsPred_Concat = pd.concat([ObsPred_df1, ObsPred_df2, ObsPred_df3, ObsPred_df4, ObsPred_df5]) # merge y_pred from all 5 folds
rs1['diagnosis_pred'] = ObsPred_Concat['diagnosis_pred'] # map y_pred values onto original matrix for control analyses
print ('Mean accuracy score across ' + str(FoldNr) + ' outer Folds: %.3f (%.3f); Minimum: %.3f; Maximum: %.3f' % (np.mean(outerloop_acc), np.std(outerloop_acc), np.min(outerloop_acc), (np.max(outerloop_acc))))
print ('Mean sensitivity score across ' + str(FoldNr) + ' outer Folds: %.3f (%.3f); Minimum: %.3f; Maximum: %.3f' % (np.mean(outerloop_sens), np.std(outerloop_sens), np.min(outerloop_sens), np.max(outerloop_sens)))
print ('Mean specificity score across ' + str(FoldNr) + ' outer Folds: %.3f (%.3f); Minimum: %.3f; Maximum: %.3f' % (np.mean(outerloop_spec), np.std(outerloop_spec), np.min(outerloop_spec), np.max(outerloop_spec)))
print ('Mean PPV score across ' + str(FoldNr) + ' outer Folds: %.3f (%.3f); Minimum: %.3f; Maximum: %.3f' % (np.mean(outerloop_PPV), np.std(outerloop_PPV), np.min(outerloop_PPV), np.max(outerloop_PPV)))
print ('Mean NPV score across ' + str(FoldNr) + ' outer Folds: %.3f (%.3f); Minimum: %.3f; Maximum: %.3f' % (np.mean(outerloop_NPV), np.std(outerloop_NPV), np.min(outerloop_NPV), np.max(outerloop_NPV)))
print ('Mean AUC score across ' + str(FoldNr) + ' outer Folds: %.3f (%.3f); Minimum: %.3f; Maximum: %.3f' % (np.mean(outerloop_AUC), np.std(outerloop_AUC), np.min(outerloop_AUC), np.max(outerloop_AUC)))

# --------------------------------- Format for export -----------------------
Results.append(
    {
        'Accuracy_Mean': np.mean(outerloop_acc),
        'Accuracy_SD':  np.std(outerloop_acc),
        'Sensitivity_Mean':  np.mean(outerloop_sens),
        'Sensitivity_SD':  np.std(outerloop_sens),
        'Specificity_Mean':   np.mean(outerloop_spec),
        'Specificity_SD':  np.std(outerloop_spec),
        'PPV_Mean':   np.mean(outerloop_PPV),
        'PPV_SD':  np.std(outerloop_PPV),
        'NPV_Mean':   np.mean(outerloop_NPV),
        'NPV_SD':  np.std(outerloop_NPV),
        'AUC_Mean':   np.mean(outerloop_AUC),
```

```
        'AUC_SD':  np.std(outerloop_AUC),
    }
)

# --------- Find number of times same feature was selected across folds --------
fn = pd.DataFrame(mrmr_features_names)
flat_fn = fn.values.flatten()
count_fn = Counter(flat_fn).most_common()

f = pd.DataFrame(mrmr_features)
flat_f = f.values.flatten()
count_f = Counter(flat_f).most_common()
seen = set()
dupes = [x for x in flat_f if x in seen or seen.add(x)] # Generates list of duplicates (NB: reports values more than once if these appear more than twice)
print('Feature (indices) that were selected more than once: %s', dupes)


# In[107]:


# SAVE OUTPUTS

# Save Outerloop Results to csv
Results = pd.DataFrame(Results)
Results['Channel'] = ch
Results.set_index('Channel', inplace=True)
Results.to_csv('/Volumes/Ashur DT2/hctsa analysis/random_segment1/SVM_results_on_hctsa_features/'+ch+'/RS1_'+ch+'_hctsa_Main_OuterloopResults.csv')
# Save list of Features selected by each of the 5 folds
fn.to_csv('/Volumes/Ashur DT2/hctsa analysis/random_segment1/SVM_results_on_hctsa_features/'+ch+'/RS1_'+ch+'_hctsa_Main_SelectedFeatures_PerFold.csv')
# Save Count of how many times each feature was selected across the 5 folds
count_fn = pd.DataFrame(count_fn)
count_fn.columns =['FeatureName', 'TimesSelected']
count_fn.to_csv('/Volumes/Ashur DT2/hctsa analysis/random_segment1/SVM_results_on_hctsa_features/'+ch+'/RS1_'+ch+'_hctsa_Main_SelectedFeatures_CountAcrossFolds.csv')
```

Section 6: Supplementary Material References

1. Noble WS. What is a support vector machine? *Nature biotechnology*. 2006;24(12):1565-1567. doi:10.1038/nbt1206-1565

2. Pedregosa F, Varoquaux G, Gramfort A, et al. Scikit-learn: Machine learning in Python. *the Journal of machine Learning research*. 2011;12:2825-2830. doi:10.48550/arXiv.1201.0490

3. Hsu CW, Chang CC, Lin CJ. A practical guide to support vector classification. Published online 2003.

4. Fulcher BD, Jones NS. hctsa: A computational framework for automated time-series phenotyping using massive feature extraction. *Cell systems*. 2017;5(5):527-531.

5. Fulcher BD, Little MA, Jones NS. Highly comparative time-series analysis: the empirical structure of time series and their methods. *Journal of the Royal Society Interface*. 2013;10(83):20130048. doi:10.1098/rsif.2013.0048

6. Hegger R, Kantz H, Schreiber T. Practical implementation of nonlinear time series methods: The TISEAN package. *Chaos: An Interdisciplinary Journal of Nonlinear Science*. 1999;9(2):413-435.

7. Larracy R, Phinyomark A, Scheme E. Machine learning model validation for early stage studies with small sample sizes. In: IEEE; 2021:2314-2319. doi:10.1109/EMBC46164.2021.9629697

8. Ding C, Peng H. Minimum redundancy feature selection from microarray gene expression data. *Journal of bioinformatics and computational biology*. 2005;3(02):185-205. doi:10.1142/S0219720005001004

9. Miles J. R-squared, adjusted R-squared. *Encyclopedia of statistics in behavioral science*. Published online 2005. doi:10.1002/0470013192.bsa526

10. Rossi F, Lendasse A, François D, Wertz V, Verleysen M. Mutual information for the selection of relevant variables in spectrometric nonlinear modelling. *Chemometrics and intelligent laboratory systems*. 2006;80(2):215-226. doi:10.1016/j.chemolab.2005.06.010

11. Guyon I, Weston J, Barnhill S, Vapnik V. Gene selection for cancer classification using support vector machines. *Machine learning*. 2002;46(1):389-422. doi:10.1023/A:1012487302797

12. Altman N, Krzywinski M. The curse (s) of dimensionality. *Nat Methods*. 2018;15(6):399-400. doi:10.1038/s41592-018-0019-x

13. Fisher RA. The use of multiple measurements in taxonomic problems. *Annals of eugenics*. 1936;7(2):179-188. doi:10.1111/j.1469-1809.1936.tb02137.x

14. Breiman L. Random forests. *Machine learning*. 2001;45(1):5-32.

15. Qi Y. Random forest for bioinformatics. In: *Ensemble Machine Learning*. Springer; 2012:307-323. 10.1007/978-1-4419-9326-7_11

16. Van der Maaten L, Hinton G. Visualizing data using t-SNE. *Journal of machine learning research*. 2008;9(11).
